# Prompting Strategies for Readability Alignment Between User-Generated Ophthalmology Questions and Large Language Model Answers: Comparative Evaluation Study

**DOI:** 10.2196/97408

**Published:** 2026-11-05

**Authors:** Mário César Bulla, Daniel Lavinsky, Alexandre Antonio Marques Rosa, Pedro Carlos Carricondo

**Affiliations:** 1 Faculdade de Medicina, Universidade de São Paulo, São Paulo, Brazil, 55 51-98118-5252; 2 Clínica Bulla, Rua São Joaquim 611, Suite 1203São Leopoldo, 93010-190, Brazil, 55 51-98118-5252; 3 Faculdade de Medicina, Universidade Federal do Rio Grande do Sul, Porto Alegre, Rio Grande do Sul, Brazil; 4 Faculdade de Medicina, Universidade Federal do Pará, Belém, Pará, Brazil

**Keywords:** readability, health communication, health literacy, ophthalmology, artificial intelligence, natural language processing

## Abstract

**Background:**

Accessible health communication is essential for effective patient education, yet most medical information remains written at a level above the general population’s recommended reading level. Large language models (LLMs) can simplify complex medical information, but it remains unclear whether responses can be automatically adapted to match the linguistic complexity of user-generated questions seeking medical information.

**Objective:**

To evaluate whether the LLM-generated responses to user-generated ophthalmology questions align with their readability and to compare different prompting strategies for readability control across multiple LLM architectures, including their impact on response quality as well as computational cost and efficiency.

**Methods:**

A total of 400 user-generated ophthalmology questions collected from public YouTube comments were categorized into 3 readability tiers based on the Flesch Reading Ease Score. A controlled comparative evaluation of responses generated by 4 LLMs (GPT 5.2, Gemini 3, Claude Opus 4.5, and OSS-20b) was performed under 4 prompting conditions: Baseline (no readability control), Formula-Informed Readability Prompt, Semantic Readability Inference, and External Calculator–Guided Prompt. Agreement between question and response readability was assessed using quadratic-weighted Cohen kappa. Response quality was evaluated using BERTScore and LLM-as-a-judge assessments. Computational efficiency was evaluated through token usage, estimated cost per response, and latency.

**Results:**

The external calculator–guided strategy produced the strongest alignment in readability across models, achieving the highest agreement (Gemini 3: κ=0.94; GPT 5.2: κ=0.90), while maintaining favorable computational efficiency. Formula-informed prompting showed intermediate performance, whereas semantic inference demonstrated poor agreement due to unreliable readability estimation. High-performing models (GPT 5.2 and Gemini 3) maintained very high factual accuracy (>98%) across strategies, indicating that readability control did not substantially degrade correctness. In contrast, the open-source OSS-20b model showed lower accuracy (83.75%‐90%) and greater sensitivity to readability constraints. Semantic similarity remained high across models (Bidirectional Encoder Representations from Transformers Score [BERTScore] *F*_1_>0.87). Longer questions were associated with slightly lower accuracy, particularly in lower-performing configurations.

**Conclusions:**

Aligning LLM responses to the readability of user-generated questions is feasible and can be reliably achieved using externally guided readability signals. Hybrid pipelines that combine traditional readability metrics with LLM-generated content may therefore represent a practical approach for scalable, readability-adaptive health communication systems.

## Introduction

Accessible health communication is essential for patient education, yet much of the health information available to patients remains above the recommended reading level [1]. As patients increasingly use digital resources and large language models (LLMs) to obtain information about diseases, treatments, and postoperative care, the usefulness of these tools depends on whether their responses are understandable to the intended audience [2-9]. Across medical specialties, unconstrained LLM responses frequently exceed the reading levels recommended by organizations such as the American Medical Association and the National Institutes of Health [10,11]. Although plain-language or grade-specific prompts can improve readability, these improvements may be inconsistent or come at the expense of completeness and accuracy [9,12-21].

Patients with retinal disease, glaucoma, or uveitis often face complex decisions involving invasive procedures and long-term treatments, making readable and accurate patient education particularly important. Recent studies indicate that LLMs can improve the readability of ophthalmic educational materials [20-23]. However, most have relied on broad plain-language instructions or fixed targets, such as a sixth-grade reading level. Such uniform targets may not reflect the variation in patients’ vocabulary and linguistic complexity. Using the readability of the patient’s question as a reference could help avoid both under-explanation and cognitive overload, consistent with evidence that communication tailored to patient literacy and language style may improve comprehension, engagement, and health outcomes [24-27].

However, to our knowledge, no previous study has systematically measured the readability of real user questions and used it as a reference to align the readability of LLM-generated answers. Although recent work, including RephQA (Readability of LLMs in Public Health Question-Answering) and related studies on readability-aware health question answering, has emphasized the importance of evaluating communicative effectiveness and language adaptation, it has primarily focused on benchmarking, simulated reader conditions, or linguistic perturbations rather than explicitly enforcing question-answer readability matching during generation [12,19,20,28-30]. This gap is clinically and conceptually important. A patient-centered communication system should ideally adapt the complexity of its answer to the user’s linguistic profile, rather than applying a uniform readability target to all users. Readability formulas such as Flesch Reading Ease Score (FRES) and Flesch-Kincaid Grade Level (FKGL), despite their limitations, provide simple, reproducible proxies for textual complexity.

To address this gap, this study evaluated whether the readability of LLM-generated responses can be automatically aligned with that of user questions. We compared 3 readability-alignment strategies across 3 commercial LLMs and 1 open-source model, evaluating readability matching, response quality (accuracy, completeness, and empathy), and computational efficiency. This formative, predeployment evaluation aimed to establish technical feasibility and identify which prompting architecture reliably controls question-answer readability alignment, as a necessary step before summative evaluation of patient-level comprehension outcomes.

## Methods

### Overview of Study Design

This was a controlled comparative evaluation study of 3 commercial LLMs and 1 open-source model. Using a standardized dataset of user questions, we generated responses under 3 readability-control prompting strategies. The primary outcome was question-answer readability alignment across a baseline condition and 3 readability-control strategies. Secondary outcomes included response quality, specifically accuracy, completeness, and empathy; the effects of prompting strategy and question characteristics, including readability and length, on accuracy; and computational efficiency, assessed through token use, latency, and estimated cost.

A study protocol was prepared but not publicly registered; the relevant section is available from the corresponding author upon reasonable request.

### Question Collection and Selection

Questions were collected via the YouTube (Google LLC) API between December 1 and 10, 2025, from public comments on ophthalmology-related videos posted by ophthalmologist-led and institutional channels. Comments were initially screened for clinical relevance and semantic coherence. Entries without meaningful ophthalmic content, such as greetings, emojis, unrelated remarks, or ambiguous statements lacking a clear medical question, were excluded. Only questions written in English were retained.

This process yielded a larger source pool of ophthalmic questions spanning multiple topics, including retinal diseases, ophthalmic symptoms, and surgical interventions such as retinal detachment, vitreoretinal surgery, age-related macular degeneration, cataract, and refractive surgery. Stratified random sampling was used to select 400 questions, with predefined quotas to ensure representation across readability (basic, intermediate, or higher, based on the FRES) and question length (<50, 50‐98, or ≥99 words). All questions were preserved in their original, unedited form, including spelling, syntax, and vocabulary, without grammatical correction or simplification, in order to preserve ecological validity and reflect real-world patient inquiries. Additionally, all questions were classified by an ophthalmologist according to their thematic domain and question type (factual or judgment-based, depending on whether they primarily sought objective information or decision-making guidance).

### LLMs

Each question was submitted independently to 4 LLMs: GPT 5.2 (gpt-5.2; OpenAI), Claude Opus 4.5 (claude-opus-4-5-20251101; Anthropic), Gemini 3 (gemini-3-pro-preview; Google), and GPT-OSS-20b (openai/gpt-oss-20b; OpenAI). The inclusion of both proprietary and open-source models was intended to evaluate whether readability alignment strategies generalize across different model architectures and deployment paradigms. According to information reported by the model developers, the knowledge cutoffs were August 31, 2025, for GPT 5.2; January 2025 for the Gemini 3 series; May 2025 for Claude Opus 4.5; and June 1, 2024, for OSS-20b. Detailed proprietary training data and model architectures were not publicly available.

All inferences were performed programmatically via Python using the respective APIs between December 23, 2025, and January 15, 2026. GPT 5.2, Claude Opus 4.5, and Gemini 3 were accessed through their respective developers’ official APIs, whereas GPT-OSS-20b was accessed through OpenRouter (OpenRouter, Inc). No provider-specific routing configuration was specified for GPT-OSS-20b; OpenRouter’s default routing was used. Each question-response pair was generated in isolation, with no conversational memory or contextual carryover between questions. The Python script also logged response latency, token usage (input and output tokens), and, when applicable to the specific strategy, the readability score inferred directly by the model. Models were tested with a temperature of 0.2 and, where supported, top-k of 40; top-k was not available for GPT 5.2.

### Readability Assessment and Categorization

Readability of both questions and LLM-generated responses was evaluated using the Textstat Python library, with FRES selected as the primary readability metric. FRES was calculated using Textstat version 0.7.11. Because FRES is conventionally interpreted on a 0 to 100 scale, although the formula itself is unbounded, negative outputs were clipped to 0. This lower boundary occurred in 26/400 (6.5%) questions and 153/6400 (2.4%) generated responses; no values exceeded 100, so no upper-bound clipping was required. FRES was chosen primarily because of its widespread use in health communication research, which facilitates comparison with previous studies, and because its continuous score provides greater granularity than formulas expressed as discrete grade-level estimates. This was particularly relevant for our dataset of real user-generated questions, many of which were short, as FRES was considered less susceptible to instability related to text length than exact grade-level measures such as the FKGL. Its continuous and readily interpretable scale also supported the classification of questions into broad readability categories and enabled the same standardized external signal to be applied to question stratification, prompt guidance, and question-response readability comparisons.

Because readability formulas vary across metrics and computational implementations [31], we classified questions into 3 broad readability levels rather than relying on exact grade-level estimates: basic (FRES>69), intermediate (FRES 30‐69), or higher (FRES<30) [24]. For the basic and higher categories, prompts were calibrated to the lowest grade level within each category to avoid overestimating the required educational level. For the intermediate category, the target was set at the midpoint of its defined grade range to reduce misclassification caused by threshold-adjacent scores.

In addition, secondary readability indices (FKGL, Gunning Fog Index, and Dale-Chall Readability Formula) were calculated, and question-response agreement was recalculated with each index as a robustness analysis.

### Readability Alignment Conditions

#### Overview

Each question was submitted to the LLMs under 4 prompting conditions: a Baseline condition without readability control and 3 readability-alignment strategies designed to control the relationship between question readability and response readability. The full prompt templates are provided in Multimedia Appendix 1. The prompt templates were defined a priori based on established best practices and experience from a previous study, while maintaining consistent safety, length, and formatting instructions across conditions. The evaluation dataset was not used to refine the prompts.

#### Baseline (No Readability Control)

In the Baseline condition, the model was asked to answer the patient’s question without any explicit instruction regarding readability level, educational complexity, or linguistic adaptation. This condition served as the reference for comparison with the readability-alignment strategies.

#### Strategy 1: Formula-Informed Readability Prompt (FRES Prompt)

This approach relied on explicit disclosure of the readability metric. The model was provided with the full name and formula of the FRES and instructed to first estimate the FRES of the input question, assign it to 1 of 3 predefined readability tiers, and then generate a response at the corresponding level.

#### Strategy 2: Semantic Readability Inference (Semantic)

This strategy adopted a more implicit and human-like approach. Instead of readability formulas or numerical indices, the model received a brief instruction to infer the educational and linguistic complexity of the question directly from its wording, sentence structure, and vocabulary, and to adjust the response accordingly. This approach was motivated by prior work showing that LLMs can modulate linguistic complexity when conditioned on reader profiles or educational levels, using semantic and linguistic cues rather than explicit readability formulas [30]. However, unlike previous studies based on predefined or simulated reader categories, the current strategy evaluated whether such adaptation could be achieved directly from real patient-generated input, without external specification of the target readability level [29,30].

#### Strategy 3: External Calculator–Guided Prompt (Textstat)

In the third strategy, the FRES of each question was first calculated externally by a Python script using the Textstat library, and a brief instruction specifying the corresponding educational level was added to the prompt. Readability estimation was thus handled entirely outside the model, leaving the LLM responsible only for generating a response at the externally defined level. Unlike strategy 1, the model received no information about the underlying readability formula.

### Response Quality Evaluation

#### Ground Truth Generation

For each of the 400 questions, a board-certified ophthalmologist (MCB) with extensive clinical experience prepared a reference answer. To standardize written structure and formatting across the dataset, the final text of these reference answers was refined with the assistance of GLM 4.7 (Z.ai, LLM) under ophthalmologist supervision. All reference answers were reviewed and approved by the ophthalmologist, who was responsible for their factual accuracy, completeness, and clinical appropriateness.

#### Automated Similarity Assessment

Semantic similarity between model-generated responses and the ground truth was quantified using Bidirectional Encoder Representations from Transformers Score (BERTScore) [32] with the Robustly Optimized BERT Pretraining Approach (RoBERTa)-large backbone, implemented via the bert-score Python library and Hugging Face Transformers (v4.57.3). We report the standard BERTScore *F*_1_ metric. Inverse document frequency weighting was not applied, and baseline rescaling was disabled. The evaluation was conducted in English, using the default layer configuration for RoBERTa-large in bert-score (17 layers) and a batch size of 16. This metric was used to assess content overlap while accounting for semantic equivalence rather than surface-level lexical similarity.

#### LLM-as-a-Judge Evaluation

Response quality was further assessed using an LLM-as-a-judge framework [33]. Generated responses were compared against the ground truth using a best-of-3 voting strategy, in which 2 independent LLMs initially evaluated each response (Qwen3-235B-A22B-Instruct-2507, Qwen team/Alibaba Cloud; DeepSeek V3, DeepSeek AI). In cases of disagreement, a third LLM served as a tiebreaker (Llama-3.3-70B-Instruct, Meta Platforms, Inc). This approach was chosen to mitigate individual model bias and is consistent with prior work demonstrating that multi-LLM evaluation frameworks improve reliability compared to single-judge systems [34].

Factual accuracy was evaluated using binary judgments (“1” for accurate and “0” for inaccurate), as earlier studies have demonstrated good agreement between binary LLM-as-a-judge assessments and human evaluations, supporting its reliability for structured correctness assessment [29,33,35-39]. A score of 1 indicated that the response did not contradict the reference answer and contained no clinically incorrect information. The response could include additional information or omit details from the reference answer without being classified as inaccurate, provided that the information presented remained clinically correct. A score of 0 indicated the presence of clinically incorrect information or a contradiction of the reference answer.

However, a binary scheme was considered insufficient to capture gradations in informational coverage and empathic communication. Therefore, completeness and empathy were evaluated using separate 3-level ordinal scales [36]. For completeness, a score of 1 indicated that the response did not address any of the explicit medically relevant questions contained in the user’s message. A score of 2 indicated that the response addressed some, but not all, of the explicit medically relevant questions. A score of 3 indicated that all explicit medically relevant questions were addressed. Nonmedical, nonsubstantive, or conversational remarks were not considered items requiring a response for the completeness assessment. For empathy, a score of 1 denoted dismissive, hostile, blaming, shaming, or clearly insensitive communication. A score of 2 denoted neutral and respectful communication without explicit emotional acknowledgment; such a response could be fully appropriate for primarily factual questions containing no emotional cue and was not considered poor or unacceptable. A score of 3 denoted explicit emotional acknowledgment or support in addition to the substantive response.

#### Human Validation

To validate the LLM-as-a-judge framework, a senior ophthalmologist (MCB) and an ophthalmology resident independently rated the same 640 question-response pairs using identical prespecified criteria; the sample was enriched for LLM-rated errors to approximately balance accurate and inaccurate ratings. Agreement was assessed between each evaluator and the LLM judge and between evaluators. Because of this enrichment, the sample does not reflect error prevalence in the full dataset.

#### Computational Cost Analysis

To address scalability and sustainability concerns, we quantified the additional computational cost associated with each readability-alignment strategy. Token usage (input and output tokens), inference time, and estimated API cost were recorded for all responses. Cost estimates were calculated by multiplying provider-reported pricing per million tokens by the observed token usage for each response, separately accounting for input and output tokens.

### Statistical Analysis

Agreement between question and response readability categories was assessed by pairwise comparisons of quadratic-weighted Cohen κ across all model-strategy combinations, using paired permutation tests based on 5000 valid permutations and the Holm-Bonferroni correction for multiple testing. For each configuration, 95% CIs for κ were estimated by bootstrap resampling using 1000 valid samples. Additional agreement analyses included observed agreement, mean absolute error (MAE), root mean square error (RMSE), concordance correlation coefficient (CCC), and Pearson and Spearman correlations, as appropriate.

Semantic similarity and ordinal quality outcomes (completeness and empathy) were compared using Friedman tests followed by Wilcoxon signed-rank tests with correction for multiple comparisons. Binary accuracy was compared using exact McNemar tests with Holm correction; 95% CIs were calculated using Wilson and bootstrap methods. Associations between accuracy and question characteristics, including readability and length, were examined using logistic regression. Relationships involving categorical readability levels were analyzed using contingency-table methods and tests for ordinal trends, as appropriate. Agreement between LLM-based and human evaluations was quantified using Cohen kappa, with weighted kappa for ordinal outcomes. Mean evaluator ratings were compared using 2-sided paired permutation tests, with paired-bootstrap 95% CIs and Holm correction across the 9 comparisons.

Token usage, latency, and estimated cost were summarized using median (IQR) and compared using Friedman and Wilcoxon signed-rank tests. Paired differences between configurations are reported as Hodges-Lehmann estimates with exact distribution-free 95% CIs. All tests were 2-sided, and *P*<.05 was considered statistically significant after adjustment. All analyses were performed in Python (version 3.11).

### Ethical Considerations

This study used publicly available information and did not involve human participants or identifiable personal information; therefore, in accordance with Brazilian regulations (National Health Council Resolution No. 510/2016), ethics committee approval was not required. No account metadata were collected, and no attempt was made to identify individual users. Example questions reproduced in the multimedia appendices are paraphrased to reduce traceability; analyses used the original texts.

## Results

### Question Characteristics

Of 3011 user-generated ophthalmic questions collected from public YouTube comments (median FRES 69.99, IQR 57.18‐79.94), 1577/3011 (52.37%) had basic readability, 1294/3011 (42.98%) intermediate, and 140/3011 (4.65%) higher. From this pool, 400 questions were randomly selected, with at least 100 per readability and question length tier.

The final sample had a median FRES of 63.62 (IQR 28.37‐75.26). The questions covered a broad range of ophthalmologic topics, predominantly related to retinal disease, vitreous symptoms, and common surgical interventions (Table 1). According to the predefined criteria, 160 questions (40%) were classified as basic readability, 127 (31.75%) as intermediate, and 113 (28.25%) as higher readability.

**Table 1.** Thematic distribution, intent classification, and readability level of user-generated questions (N=400).

Variables	Value, n (%)
Primary themes	
Vitreous symptoms/posterior vitreous detachment/floaters	111 (27.75)
Cataract surgery (intraocular lens, light-adjustable lens, multifocal lenses, and postoperative care)	63 (15.75)
Macular and retinal vascular diseases (age-related macular degeneration, retinal vein occlusion, and macular edema)	47 (11.75)
Refractive surgery (Laser-Assisted in Situ Keratomileusis, photorefractive keratectomy, Implantable Collamer Lens)	38 (9.50)
Retinal detachment/retinal tears/retinopexy	32 (8)
Ocular surface disease/dry eye/cornea	19 (4.75)
Clinical treatments/medications/supplements	19 (4.75)
Neurologic symptoms/diplopia/strabismus	16 (4)
Refraction/glasses/prescriptions	16 (4)
Other ophthalmic surgeries	15 (3.75)
Retinal surgery (vitrectomy, epiretinal membrane, and macular pucker)	10 (2.50)
Glaucoma	8 (2)
Ocular trauma	4 (1)
Uveitis/intraocular inflammation	2 (0.50)
Question intent	
Factual	232 (58)
Judgment-based	168 (42)
Readability level	
Basic	160 (40)
Intermediate	127 (31.75)
Higher	113 (28.25)

### Readability Alignment Performance

Overall, the Textstat strategy yielded the highest question-response readability agreement across all 4 LLMs (κ=0.6911‐0.9444), followed by the FRES Prompt strategy in all models except GPT 5.2, whereas Baseline and Semantic prompting showed consistently low agreement (Figure 1 and Tables 2 and 3). Gemini 3 Textstat achieved the highest agreement (κ=0.9444), followed by GPT 5.2 Textstat (κ=0.9029), with no significant difference between them (adjusted *P*=.46). Complete pairwise comparisons, including unadjusted and Holm-adjusted P values, are provided in Multimedia Appendix 2.

**Figure 1.**
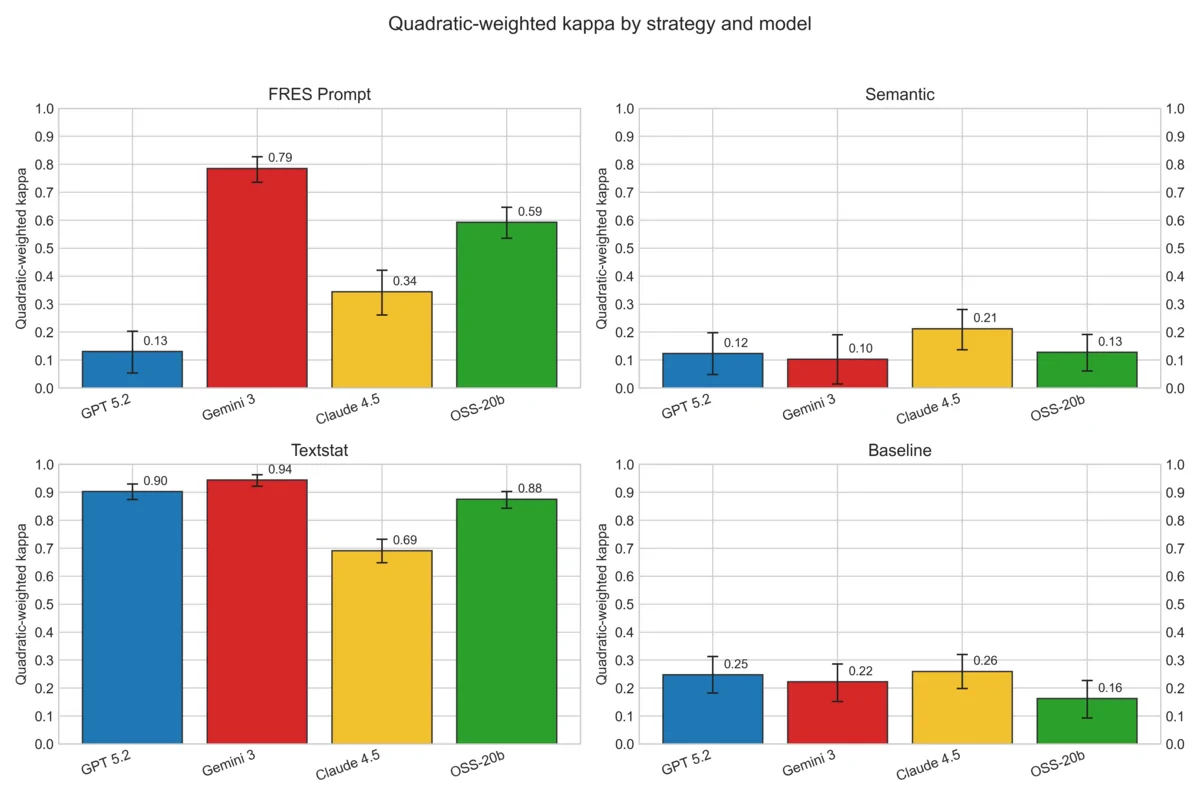
Quadratic-weighted Cohen kappa (κ) for question–response readability alignment by model and prompting strategy. Each panel represents one prompting condition (Baseline, Semantic, FRES Prompt, and Textstat), and the x-axis shows the 4 evaluated language models. Bars represent κ and error bars denote bootstrap 95% CIs, with exact values shown above each bar. κ quantifies agreement between the readability tier assigned to the original question (basic, intermediate, or higher) and the tier of the corresponding generated response; quadratic weighting penalizes 2-tier discrepancies more heavily than 1-tier discrepancies, so higher values indicate closer alignment and 0 indicates agreement no better than chance. Across all 4 models, the Textstat condition achieved the highest agreement and the Semantic condition the lowest. Pairwise comparisons with Holm-adjusted *P* values are reported in Multimedia Appendix 2. FRES: Flesch Reading Ease Score.

**Table 2.** Agreement between question and response readability categories across models, strategies, and readability formulas^a^.

Strategy and model	FRES^b^ (κ)	FKGL^c^ (κ)	Gunning fog (κ)	Dale-Chall (κ)
Textstat				
Gemini 3	0.9444	0.6969	0.7335	0.5784
GPT 5.2	0.9029	0.6680	0.7385	0.5004
OSS-20b	0.8756	0.6128	0.6814	0.5111
Claude 4.5	0.6911	0.5680	0.6339	0.4594
FRES Prompt				
Gemini 3	0.7855	0.5719	0.6358	0.5233
OSS-20b	0.5931	0.4051	0.3543	0.3171
Claude 4.5	0.3445	0.3158	0.3442	0.1950
GPT 5.2	0.1302	0.2509	0.2782	0.1484
Baseline				
Gemini 3	0.2224	0.1068	0.0434	0.0829
GPT 5.2	0.2474	0.1227	0.0393	0.0257
Claude 4.5	0.2596	0.1298	0.0259	0.0323
OSS-20b	0.1627	0.1202	0.0604	0.0652
Semantic				
Gemini 3	0.1032	0.1703	0.1627	0.0993
GPT 5.2	0.1233	0.1405	0.1347	0.0116
Claude 4.5	0.2123	0.1538	0.1859	0.0395
OSS-20b	0.1280	0.1526	0.1288	0.0409

a Values are quadratic-weighted Cohen κ for agreement between the readability category of the original question and that of the generated response (N=400 question-response pairs per model-strategy configuration); for each formula, questions and responses were categorized using that same formula.

b FRES: Flesch Reading Ease Score.

c FKGL: Flesch-Kincaid Grade Level.

**Table 3.** Summary of readability alignment (κ) and response quality across models and prompting strategies.

Strategy and model	κ^a^ (95% CI)	Accuracy, %^b^ (95% CI)	Completeness^c^ (95% CI)	Empathy^c^ (95% CI)
Textstat				
Gemini 3	0.9444 (0.9229‐0.9616)	98.25 (96.43‐99.15)	2.80 (2.75‐2.83)	2.62 (2.57‐2.67)
GPT 5.2	0.9029 (0.8743‐0.9284)	99.75 (98.6‐99.96)	2.80 (2.76‐2.84)	2.13 (2.09‐2.16)
OSS-20b	0.8756 (0.8410‐0.9034)	85.5 (81.71‐88.61)	2.63 (2.58‐2.67)	2.30 (2.25‐2.34)
Claude 4.5	0.6911 (0.6471‐0.7334)	95.75 (93.3‐97.33)	2.90 (2.87‐2.93)	2.79 (2.75‐2.83)
FRES Prompt				
Gemini 3	0.7855 (0.7371‐0.8283)	98.5 (96.77‐99.31)	2.82 (2.78‐2.85)	2.65 (2.60‐2.69)
OSS-20b	0.5931 (0.5301‐0.6490)	83.75 (79.82‐87.04)	2.62 (2.57‐2.67)	2.37 (2.32‐2.41)
Claude 4.5	0.3445 (0.2680‐0.4227)	97 (94.83‐98.28)	2.96 (2.94‐2.98)	2.74 (2.69‐2.78)
GPT 5.2	0.1302 (0.0539‐0.2033)	99.5 (98.2‐99.86)	2.89 (2.86‐2.92)	2.11 (2.08‐2.14)
Baseline				
Gemini 3	0.2224 (0.1523‐0.2864)	98.75 (97.11‐99.46)	2.91 (2.88‐2.94)	2.72 (2.68‐2.77)
GPT 5.2	0.2474 (0.1827‐0.3127)	100 (99.05‐100)	2.91 (2.88‐2.94)	2.10 (2.07‐2.13)
Claude 4.5	0.2596 (0.1982‐0.3204)	97 (94.83‐98.28)	2.95 (2.93‐2.97)	2.72 (2.67‐2.76)
OSS-20b	0.1627 (0.0930‐0.2269)	90 (86.67‐92.57)	2.76 (2.72‐2.80)	2.44 (2.39‐2.49)
Semantic				
Gemini 3	0.1032 (0.0146‐0.1905)	99 (97.46‐99.61)	2.92 (2.89‐2.94)	2.60 (2.55‐2.65)
GPT 5.2	0.1233 (0.0486‐0.1980)	99.75 (98.6‐99.96)	2.94 (2.92‐2.96)	2.13 (2.10‐2.17)
Claude 4.5	0.2123 (0.1373‐0.2805)	97.25 (95.14‐98.46)	2.96 (2.94‐2.98)	2.60 (2.55‐2.65)
OSS-20b	0.1280 (0.0611‐0.1911)	86.25 (82.53‐89.28)	2.67 (2.62‐2.72)	2.27 (2.23‐2.32)

a κ: quadratic-weighted Cohen kappa for agreement between question and answer readability categories (basic, intermediate, and higher), with bootstrap 95% CIs

b Accuracy: proportion of factually correct responses (LLM-as-a-judge), with Wilson 95% CIs.

c Completeness and empathy: mean scores on a 3-point scale, with t-distribution 95% CIs.

The distribution of FRES scores similarly showed the clearest separation between the original question-readability tiers under the Textstat strategy, with less overlap and greater preservation of the expected tier ordering across models (Figure 2). The FRES Prompt showed intermediate performance, whereas Baseline and Semantic prompting tended to compress response readability toward the middle range. Most pairwise differences between readability tiers remained significant after Holm-Bonferroni correction. Detailed tier-level analyses are provided in Multimedia Appendices 3 and 4, and representative question-response examples are provided in Multimedia Appendices 5 and 6.

**Figure 2.**
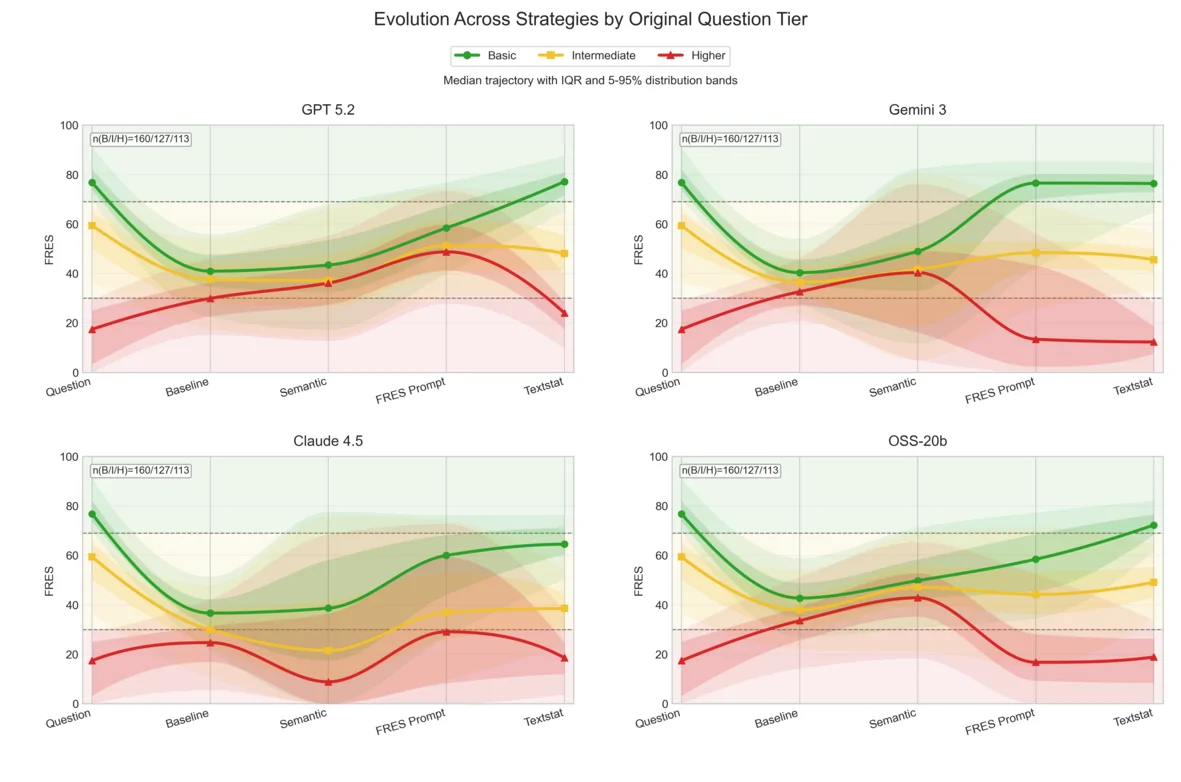
Distribution of Flesch Reading Ease Scores (FRES) across models and prompting strategies, stratified by the readability tier of the original question. Each panel represents one language model, and the x-axis shows the original question and responses generated under the Baseline, Semantic, Flesch Reading Ease Score (FRES) Prompt, and Textstat conditions. Colors indicate the original question-readability tier: Basic (green), Intermediate (yellow), and Higher (red). Higher FRES values indicate easier-to-read text. Markers represent median FRES values, with connecting lines shown only as visual guides. Inner shaded bands indicate the IQR (Q25-Q75), and outer shaded bands indicate the 5th-95th percentile range; wider bands therefore reflect greater response variability. Dashed horizontal lines mark the predefined readability thresholds: basic (>69), intermediate (30-69), and higher (<30). The notation n(B/I/H) indicates the number of questions in each original tier: 160 basic, 127 intermediate, and 113 higher.

### Stage-Wise Analysis of Semantic Strategy Performance

To further examine the Semantic strategy, we performed a staged agreement analysis, separating readability inference from response generation. Quadratic-weighted Cohen kappa was calculated for 2 comparisons: model-inferred question readability versus the formula-derived reference level, and response readability versus the model-inferred level.

Agreement between model-inferred and reference readability was low across models (κ=−0.0397 to 0.1308), indicating limited ability to accurately infer question readability. In contrast, agreement between response readability and the model-inferred level was substantially higher (Gemini 3: κ=0.9358; Claude 4.5: κ=0.8246; OSS-20b: κ=0.5456; GPT 5.2: κ=0.5399), suggesting that once a readability level was inferred, models generally adhered to it during generation.

### Stage-Wise Analysis of FRES Prompt Strategy Performance

Across 400 samples per model, variability was observed in the LLMs’ ability to infer FRES relative to the external calculator reference (Figure 3). Gemini 3 showed the lowest MAE (7.82) and RMSE (12.13), along with a high CCC (0.893) and strong correlations with the reference (*r*=0.898; ρ=0.894). OSS-20b also showed relatively low error (MAE=10.27; RMSE=14.02) and high agreement metrics (CCC=0.861; *r*=0.881; ρ=0.830). Claude 4.5 presented higher error values (MAE=18.85; RMSE=26.14; CCC=0.330; *r*=0.423; ρ=0.436), whereas GPT 5.2 showed the highest error and lowest agreement metrics (MAE=23.66; RMSE=30.94; CCC=0.060; *r*=0.085; ρ=0.127).

**Figure 3.**
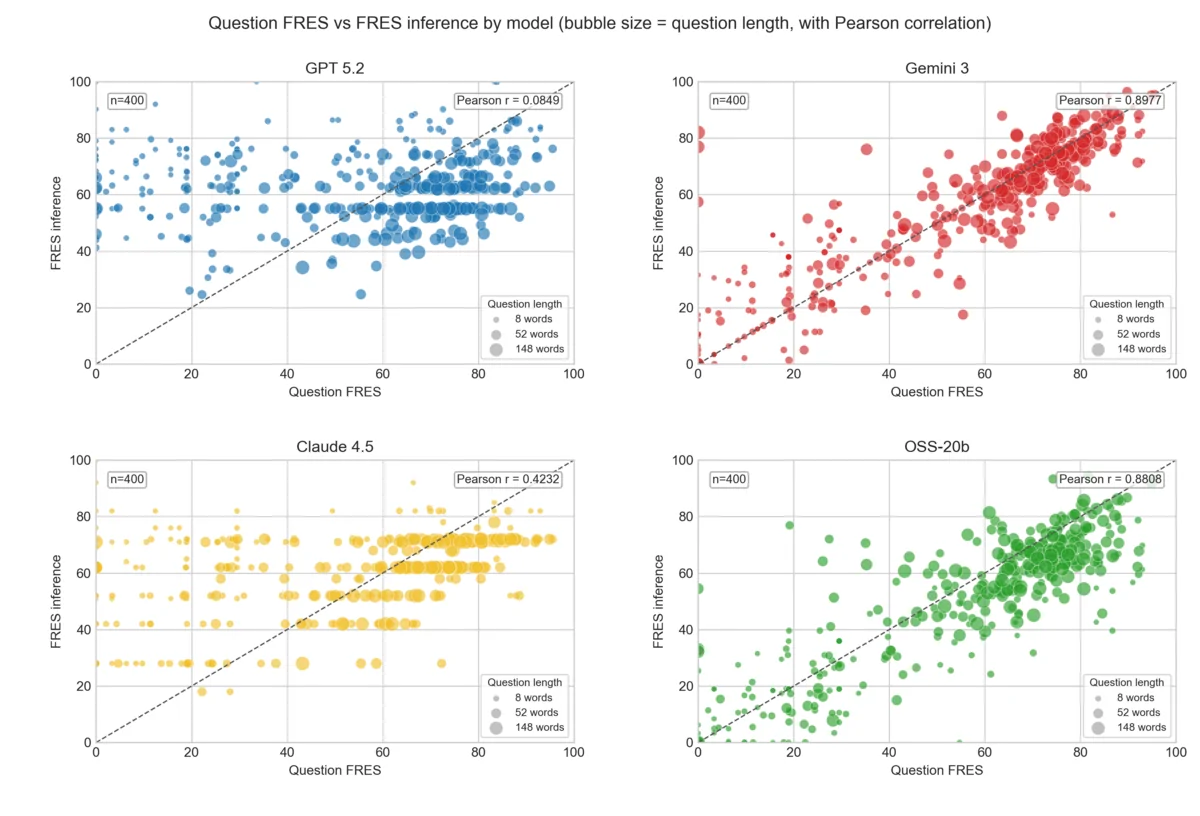
Relationship between formula-derived and model-inferred Flesch Reading Ease Score (FRES) across LLMs. Each panel represents one language model. The x-axis shows the formula-derived FRES of each original user question, whereas the y-axis shows the FRES inferred by the corresponding model. Higher FRES values indicate easier-to-read text. Each bubble represents one question and its corresponding model-inferred FRES value (n=400 per model), with bubble size proportional to question length in words. The dashed diagonal line represents perfect agreement between the formula-derived and model-inferred scores (y=x). Points above the line indicate that the model rated the question as easier to read than indicated by the formula-derived FRES, whereas points below the line indicate that the model rated it as more difficult to read. Pearson correlation coefficients (*r*) are displayed for each model and indicate the strength of the linear association between formula-derived and model-inferred FRES values; they should not be interpreted as measures of exact agreement.

### Effect of Question Length on FRES Inference Accuracy

We compared the MAE in FRES estimation between short (n=194) and long (n=103) questions, with intermediate-length questions excluded from this analysis, using Welch t tests (Figure 3). For Claude 4.5, MAE was lower for long questions than for short questions (12.21 [SD 13.54] vs 25.61 [SD 20.81]; *t*_283.02_=6.69; *P*<.001). Similarly, GPT 5.2 showed lower MAE for long questions (16.99 [SD 12.53] vs 31.73 [SD 23.33]; *t*_294.95_=7.08; *P*<.001). In contrast, Gemini 3 (7.02 [SD 11.91] vs 8.40 [SD 8.08]; *t*_153.11_=1.06; *P*=.29) and OSS-20b (9.31 [SD 7.34] vs 10.64 [SD 10.74]; *t*_276.72_=1.26; *P*=.21) showed similar MAE for long and short questions.

### Tier-Level Agreement Analysis

Tier agreement was evaluated using quadratic-weighted Cohen kappa coefficient (κ), comparing (1) the generated FRES tier versus the inferred tier and (2) the original question tier versus the inferred tier. For the generated versus inferred tier, Gemini 3 showed near-perfect agreement (κ=0.960), followed by OSS-20b with substantial agreement (κ=0.736). GPT 5.2 demonstrated moderate agreement (κ=0.441), whereas Claude 4.5 showed fair agreement (κ=0.315). When comparing the original question tier to the inferred tier, κ values were lower across all models. Gemini 3 maintained substantial agreement (κ=0.663), OSS-20b showed moderate agreement (κ=0.485), Claude 4.5 showed fair agreement (κ=0.294), and GPT 5.2 showed slight agreement (κ=0.053).

### Agreement Across Alternative Readability Formulas

Agreement was generally lower with FKGL, Gunning Fog, and Dale-Chall than with FRES, but the relative pattern was preserved: Textstat showed the highest agreement, followed by FRES Prompt, with consistently low agreement for Semantic and Baseline (Table 2).

### Quality Analysis

#### Semantic Similarity (BERTScore)

Mean *F*_1_ scores were high across all configurations (*F*_1_>0.87) and varied little across strategies within each model. For Baseline, Semantic, FRES Prompt, and Textstat, respectively, *F*_1_-score was 0.884, 0.873, 0.883, and 0.878 for Claude 4.5 and 0.903, 0.894, 0.893, and 0.893 for Gemini 3; GPT 5.2 ranged from 0.875 to 0.877, and OSS-20b from 0.872 (Textstat) to 0.878 (FRES Prompt). Although the Friedman test indicated a global difference across configurations (*χ*²_15_=2565.3; *P*<.001), within-model differences were approximately 0.01, and no pairwise difference between strategies was significant for GPT 5.2 (all Holm-adjusted *P*>.05). Between models, Gemini 3 had higher *F*_1_-score than all other models across all strategies (all adjusted *P*<.001), whereas differences between GPT 5.2 and OSS-20b were small and inconsistent. Overall, between-model differences (up to ~0.03) exceeded within-model differences across readability strategies (~0.01).

#### Accuracy (Binary LLM-as-a-Judge Evaluation)

Substantial differences were observed between models, whereas within-model strategy effects were generally small (Figure 4 and Table 3). GPT 5.2 achieved the highest accuracy (99.5%‐100% across strategies), followed closely by Gemini 3 (98.25‐99%). Claude 4.5 was intermediate (95.75%‐97.25%), whereas OSS-20b was consistently lower (83.75%‐90%; 335-360/400).

**Figure 4.**
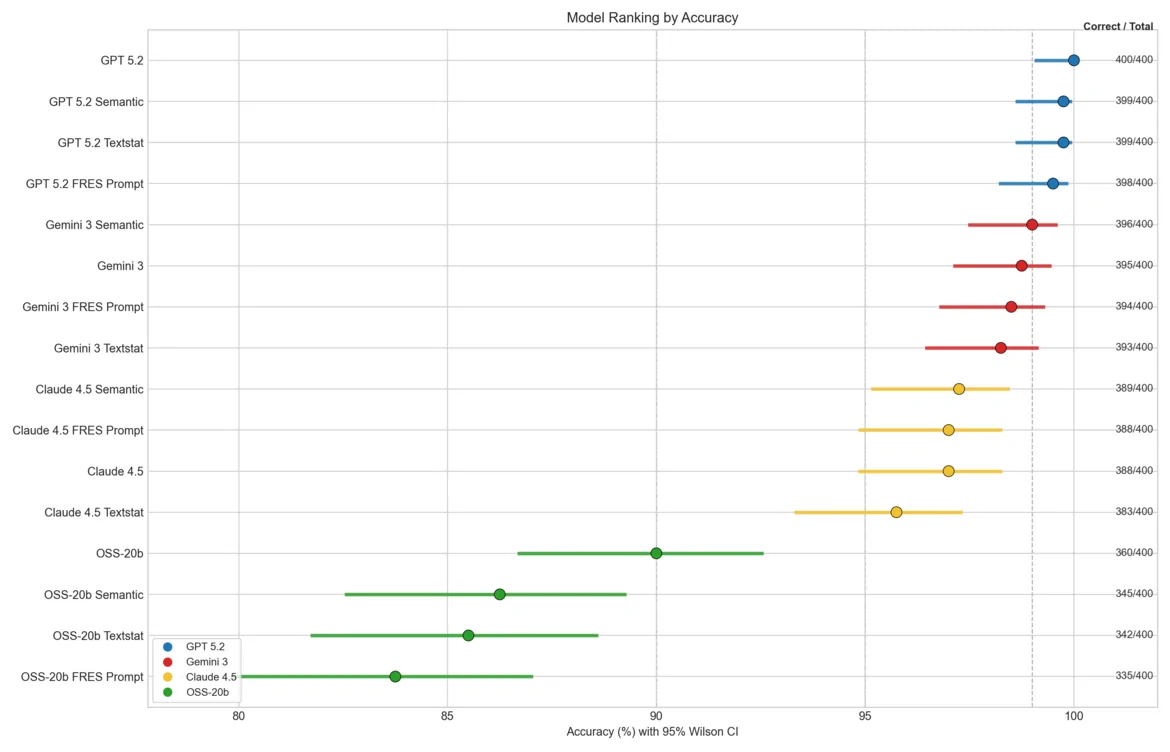
Accuracy of models across prompting strategies. Points represent accuracy (%) for each model-strategy combination, and horizontal lines indicate 95% Wilson CIs. Colors denote model families (GPT 5.2=blue, Gemini 3=red, Claude 4.5=yellow, OSS-20b=green). Values on the right indicate the number of correct responses out of 400 items. The vertical dashed line indicates the 99% accuracy threshold.

#### Between-Model Comparisons

Pairwise exact McNemar tests with Holm-Bonferroni correction showed that all GPT 5.2 and Gemini 3 configurations outperformed all OSS-20b configurations by 8.25‐16.25 percentage points (all adjusted *P*<.001). Most Claude 4.5 configurations also outperformed OSS-20b by 7.00‐13.50 percentage points (all adjusted *P*≤.001), except Claude 4.5 Textstat versus OSS-20b baseline (5.75 percentage points; adjusted *P*=.054). Among GPT 5.2, Gemini 3, and Claude 4.5, most differences were small and nonsignificant after correction; significant contrasts were limited to GPT 5.2 baseline versus Claude 4.5 baseline and FRES Prompt (3.00 points; both adjusted *P*=.03) and the 4 GPT 5.2 configurations versus Claude 4.5 Textstat (3.75‐4.25 points; adjusted *P*=.001-.049; Figure 5). Complete pairwise results are provided in Multimedia Appendix 7.

**Figure 5.**
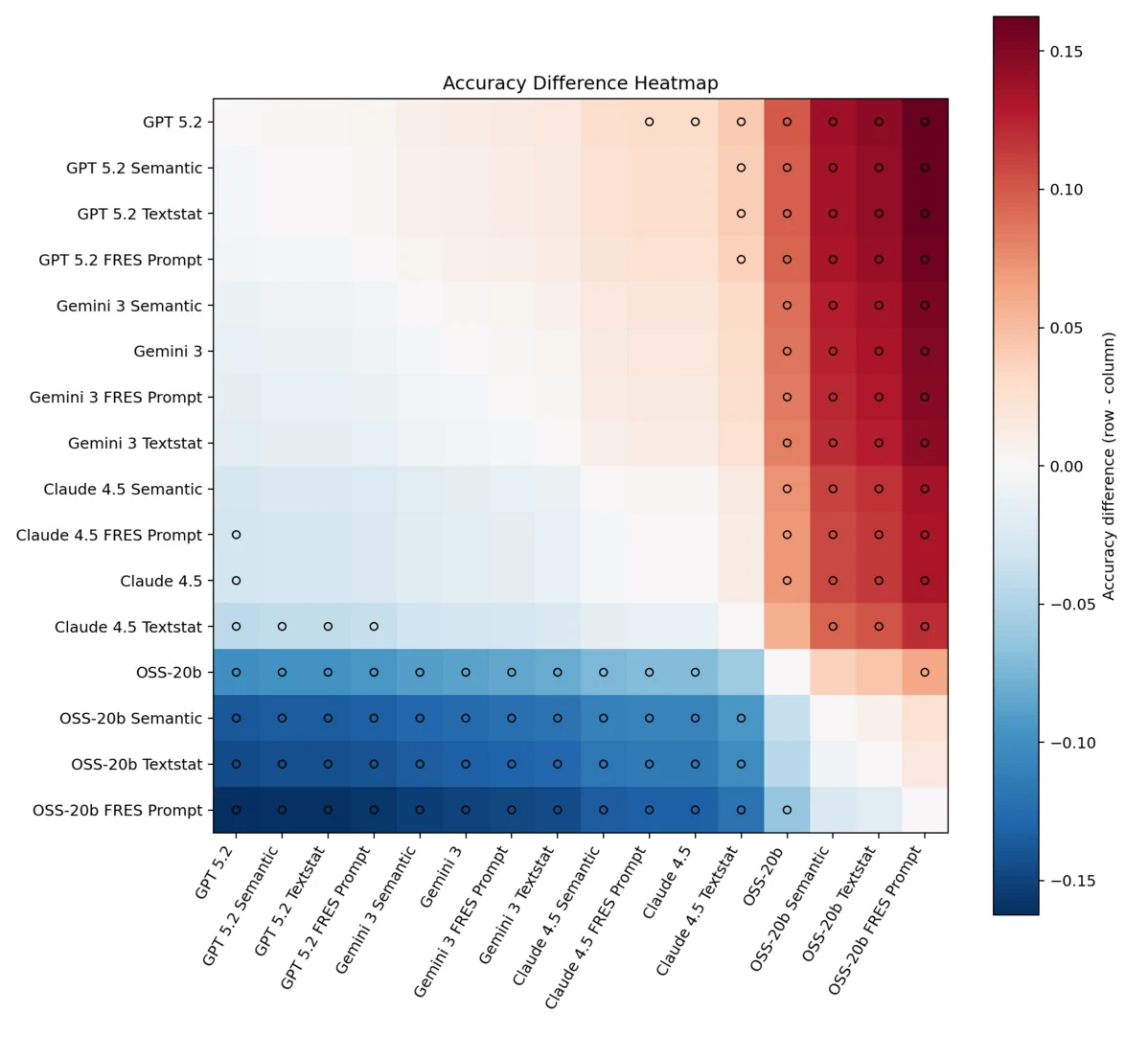
Pairwise differences in accuracy between model-strategy configurations. Rows and columns represent the evaluated combinations of language models and prompting strategies; model names shown without a strategy label represent the baseline configurations. Each cell displays the absolute difference in accuracy between 2 configurations, calculated as the accuracy of the row configuration minus that of the column configuration. Positive differences are shown in red and indicate higher accuracy for the row configuration, whereas negative differences are shown in blue and indicate higher accuracy for the column configuration. For example, a value of 0.10 corresponds to a difference of 10 percentage points. Greater color intensity indicates a larger absolute difference. The diagonal represents comparisons of each configuration with itself and therefore has a value of zero. Open circles indicate statistically significant pairwise differences according to exact McNemar tests after Holm-Bonferroni correction (adjusted *P*<.05). FRES: Flesch Reading Ease Score.

#### Within-Model Strategy Effects

Within individual models, prompting strategy had limited impact on accuracy. For GPT 5.2, Gemini 3, and Claude 4.5, no within-model comparison remained significant after Holm correction (all adjusted *P*>.99). For OSS-20b, Baseline accuracy exceeded FRES Prompt accuracy by 6.25 percentage points, the only significant within-model difference after correction (adjusted *P*=.02); all other OSS-20b comparisons were nonsignificant (all adjusted *P*≥.84).

### Association Between Readability, Question Characteristics, and Accuracy

No significant association between question readability (FRES) and accuracy was observed for GPT 5.2, Gemini 3, or Claude 4.5, whereas OSS-20b showed lower accuracy for easier questions across Baseline, Textstat, and FRES Prompt. Response readability was likewise unrelated to accuracy for GPT 5.2 and Gemini 3, but showed negative associations for Claude 4.5 Textstat and OSS-20b Textstat and FRES Prompt. Higher target response complexity was associated with slightly higher overall accuracy, an effect minimal in high-performing models but more pronounced in OSS-20b and Claude 4.5 Textstat. Question length also showed a small inverse association with accuracy, particularly for OSS-20b and the Textstat and FRES Prompt strategies, with limited practical impact in higher-performing models. Complete results are provided in Multimedia Appendix 7.

### Response Completeness Across Models and Prompting Strategies

All 16 configurations had a median completeness score of 3, although differences across configurations were significant (Friedman *χ*²_15_=723.5; *P*<.001; Kendall W=0.12). Mean scores were highest for Claude 4.5, followed by GPT 5.2 and Gemini 3, and lowest for OSS-20b (Table 3). In pairwise Wilcoxon tests with Holm-Bonferroni correction, Claude 4.5 FRES Prompt exceeded the OSS-20b configurations by 0.20 to 0.34 points, and Claude 4.5 Semantic exceeded Gemini 3 and GPT 5.2 Textstat by 0.16 (all adjusted *P*<.001). Complete pairwise results are provided in Multimedia Appendix 8.

### Response Empathy Across Models and Prompting Strategies

All Claude 4.5 and Gemini 3 configurations had a median empathy score of 3, whereas all GPT 5.2 and OSS-20b configurations had a median of 2 (Friedman *χ*²_15_=1874.6; *P*<.001; Kendall W=0.31). Mean scores were highest for Claude 4.5, followed by Gemini 3, OSS-20b, and GPT 5.2 (Table 3). In pairwise Wilcoxon tests with Holm-Bonferroni correction, all Claude 4.5 and Gemini 3 configurations outperformed all GPT 5.2 and OSS-20b configurations; for example, Claude 4.5 Textstat exceeded GPT 5.2 Baseline by 0.69 and OSS-20b Semantic by 0.52 (all adjusted *P*<.001). Complete pairwise results are provided in Multimedia Appendix 9.

### Agreement Between LLM-as-a-Judge and Human Evaluation

In the human-validation sample (n=640), agreement among the LLM judge, senior ophthalmologist, and ophthalmology resident was substantial for accuracy and empathy but moderate for completeness. For accuracy, LLM–ophthalmologist agreement was κ=0.788 (89.5% concordance), LLM–resident κ=0.745 (87.5%), and ophthalmologist–resident κ=0.664 (83.8%). For empathy, corresponding values were κ=0.791 (weighted κ=0.796; 89.7%), κ=0.736 (weighted κ=0.736; 86.7%), and κ=0.650 (weighted κ=0.649; 82.2%). Completeness showed lower agreement: LLM–ophthalmologist κ=0.436 (weighted κ=0.450; 73.3%), LLM–resident κ=0.471 (weighted κ=0.500; 76.7%), and ophthalmologist–resident κ=0.572 (weighted κ=0.575; 79.5%). Mean evaluator ratings are shown in Multimedia Appendix 10, with pairwise comparisons in Multimedia Appendix 11. The senior ophthalmologist assigned lower mean scores than the resident for accuracy, completeness, and empathy (all Holm-adjusted *P*≤.008), and lower completeness (*P*<.001) and empathy (*P*=.03) scores than the LLM judge; the accuracy difference between the ophthalmologist and LLM judge was not significant (*P*=.08).

### Computational Efficiency

Token consumption, latency, and cost all differed substantially across the 16 model-strategy combinations (Friedman *χ*²_15_=5604.6, 4268.7, and 5879.4, respectively; all *P*<.001). Descriptive statistics are provided in Table S1 and all pairwise comparisons in Tables S2-S4 in Multimedia Appendix 12, with distributions shown in Figure 6. GPT 5.2 and Claude 4.5 were the most token-efficient families and Gemini 3 the least, with its highest consumption under the FRES Prompt (median 2598 tokens). Latency distributions were skewed. Among the stable configurations, GPT 5.2 was fastest (medians 4.38‐5.60 seconds), Claude 4.5 intermediate (6.68‐7.49 seconds), and Gemini 3 slowest, particularly under the FRES Prompt (25.95 seconds). OSS-20b was by far the least expensive family but the most variable, with extreme maxima under the FRES Prompt (22,309 tokens; 461.83 seconds). Total cost for the 400 questions ranged from US $0.03 (OSS-20b) to US $12.93 (Gemini 3, FRES Prompt), and the FRES Prompt increased cost relative to Baseline in all 4 families. Most paired Wilcoxon comparisons remained significant after Holm correction; the clearest exception was OSS-20b Baseline versus Textstat for cost (adjusted *P*=.70).

**Figure 6.**
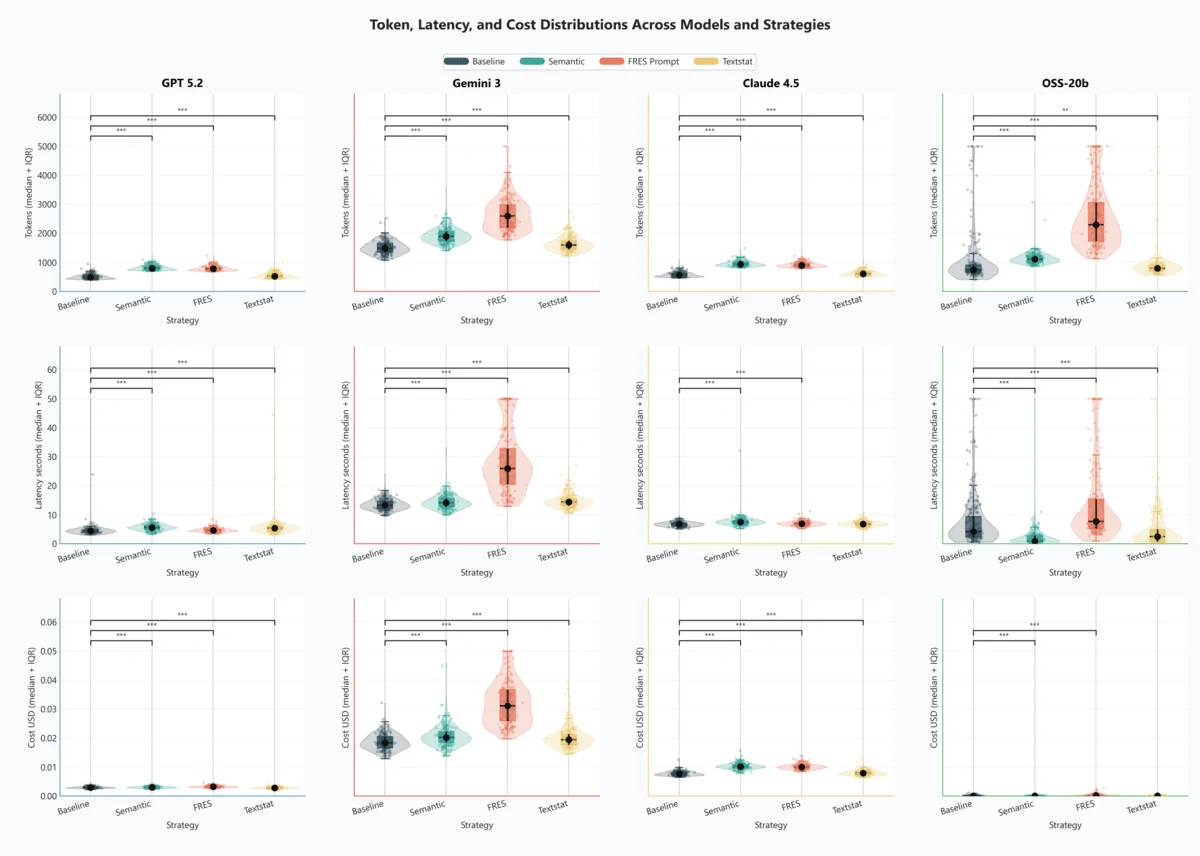
Token usage, latency, and cost across models and prompting strategies. Raincloud plots (violin, boxplot, and jittered points) show the distributions of total tokens, latency, and estimated cost per response (US $) across strategies (Baseline, Semantic, FRES Prompt, Textstat) for each model (GPT 5.2, Gemini 3, Claude 4.5, OSS-20b). Central tendency and dispersion are summarized by the median (IQR). To preserve cross-model comparability, a shared global visual cap was applied within each metric (tokens=5000, latency=50 s, cost=US $0.05); clipping affected only visualization, while all statistical tests used full, untrimmed data. Asterisks indicate statistically significant within-model comparisons versus Baseline based on paired Wilcoxon signed-rank tests. For each efficiency outcome, *P* values were adjusted using the Holm procedure across all pairwise comparisons among the 16 model–strategy configurations (*P<.05, **P<.01, ***P<.001). FRES: Flesch Reading Ease Score.

## Discussion

### Principal Results

Earlier work has shown that LLMs can generate text at predefined readability levels or estimate readability, but these capabilities have generally been studied separately rather than integrated within patient-level question answering [29,40]. To our knowledge, this study is the first to use the linguistic complexity of the user’s own question as the anchor for controlling response readability, rather than a single predefined readability target.

Without an explicit readability target, LLMs did not reliably align response complexity with that of the question, consistent with prior reports [18,41,42]. Baseline and Semantic prompting showed low agreement, whereas externally guided prompting improved alignment substantially. Textstat consistently outperformed Baseline and Semantic strategies across models, while the FRES Prompt produced intermediate and heterogeneous results, indicating that current LLMs follow explicit external readability targets more reliably than they infer them autonomously.

We therefore recommend the Textstat strategy when question-response readability matching is a design goal. Because readability is computed deterministically from the question, its performance is less dependent on model-specific estimation ability. Figure 7 presents this workflow as a reusable template based on a readability library, 3 threshold-mapped style instructions, and specialty-specific adaptation of the base prompt.

**Figure 7.**
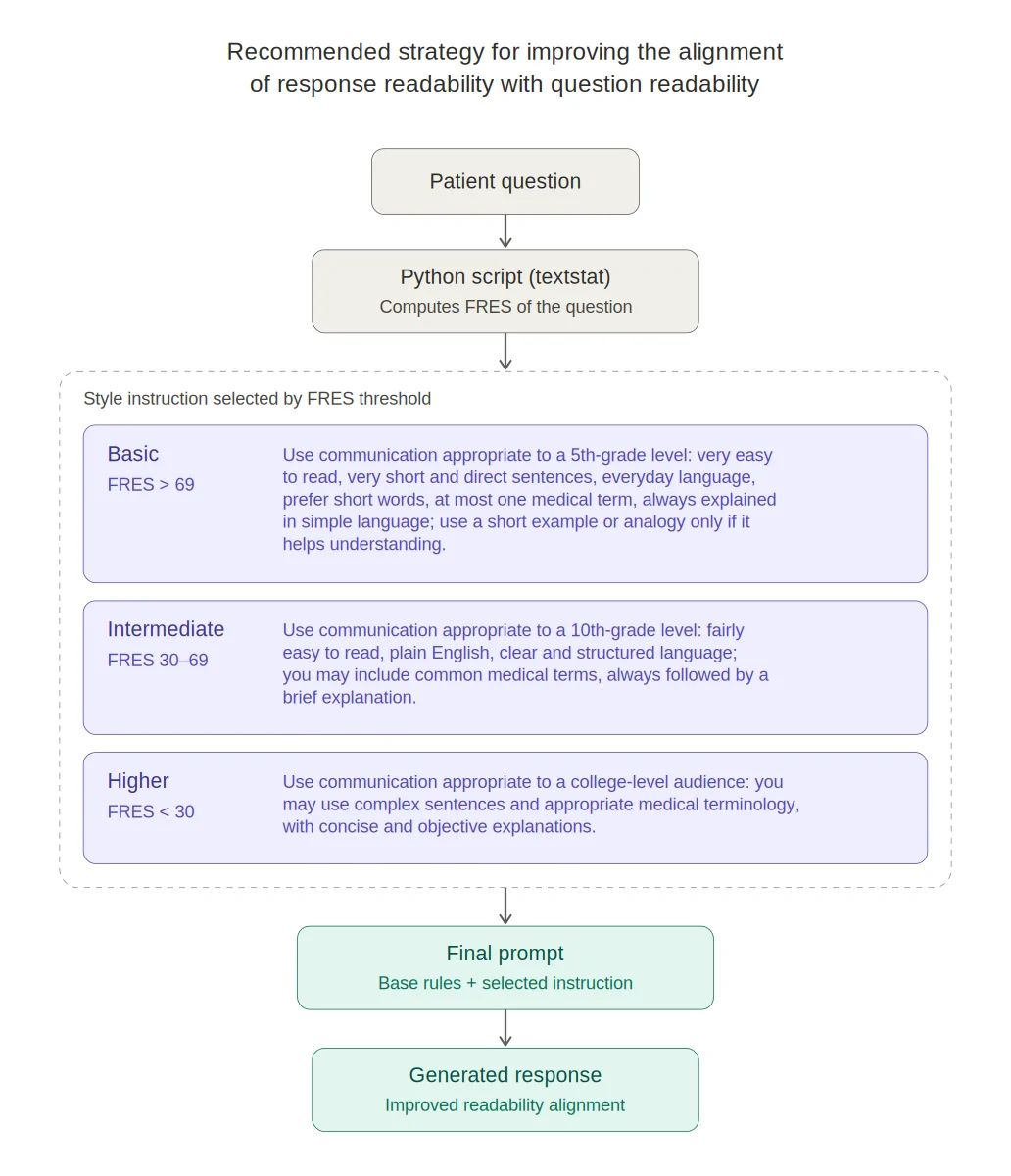
Recommended prompting workflow (Textstat strategy). A Python script computes the Flesch Reading Ease Score (FRES) of the patient’s question before any model call, and the score selects 1 of 3 style instructions (shown in full) that is appended to the base prompt. Because classification is deterministic, the template applies to any specialty by changing the specialty designation. For deployment, the experimental instruction not to disclose AI involvement (Multimedia Appendix 1) should be removed.

### Comparison With Prior Work

Our findings are consistent with studies in orthopedic trauma, gynecology, hand surgery, mixed specialties, and cardiology showing that LLMs can improve the readability of patient-facing materials [9,15,16,19,43]. However, prior work has largely focused on rewriting existing content toward fixed targets, typically a sixth-grade reading level, which models have not always achieved reliably [15,43].

Accuracy was influenced more by model architecture than by readability-control strategy. GPT 5.2 and Gemini 3 maintained very high factual accuracy across conditions, whereas OSS-20b performed consistently worse, in line with previous medical question-answering studies [18,42-47]. Readability control had little effect on accuracy in stronger models, and BERTScore remained high across strategies, although semantic similarity should complement rather than replace direct factual accuracy assessment [48]. Multidimensional evaluation further showed distinct model profiles: GPT 5.2 achieved the highest accuracy but the lowest empathy, Claude 4.5 ranked highest for completeness and empathy despite slightly lower accuracy, and Gemini 3 combined near-ceiling accuracy with the highest semantic similarity and empathy close to Claude 4.5. OSS-20b underperformed across most quality dimensions, particularly under explicit readability constraints, suggesting that controllable patient-oriented communication may be more demanding for smaller models.

Furthermore, in OSS-20b, accuracy was lower under explicit readability constraints and when responses targeted the basic level, suggesting that weaker models may face trade-offs between stylistic control and factual reliability. However, the literature does not support a universally antagonistic relationship between simplification and accuracy; simpler patient-oriented explanations can remain equally accurate, or even be preferred, when simplification improves conceptual clarity without omitting essential information [49,50]. Overall, these findings indicate that readability adaptation is not inherently detrimental to quality, but rather depends on how simplification is implemented and on the model’s capability. Although several secondary comparisons reached statistical significance, their absolute magnitudes were often small, particularly among the higher-performing commercial models. In contrast, the differences in readability alignment across prompting strategies and the lower accuracy of OSS-20b were more substantial and practically relevant.

Question characteristics also influenced LLM performance. Longer questions were associated with slightly lower accuracy, particularly in OSS-20b and in the 2 strategies whose readability target was derived from the FRES (Textstat and FRES Prompt), but not under Baseline or Semantic prompting. A possible explanation is that adhering to a FRES-based readability target while addressing the additional conditions, contextual details, or multiple sub-questions of longer questions increases task demands [51-53]. However, the practical magnitude of this effect was small in the strongest systems.

The LLM-as-a-judge framework showed encouraging agreement with human evaluation: substantial for accuracy and empathy, and moderate for completeness. The lower agreement for completeness likely reflects differences in how evaluators interpreted which portions of a comment constituted an explicit question rather than context without direct interrogative intent. This is consistent with reports that LLM-based evaluators can approach expert-level agreement when prompts, rubrics, and aggregation strategies are carefully designed, particularly under categorical rather than unconstrained scalar scoring [29,33,35-39,54-57]. Nevertheless, the approach remains recent, and the optimal models, rubrics, and scoring strategies are not yet established; its output is therefore better suited to comparative analysis than to absolute measurement of quality. Notably, agreement between our 2 human reviewers was lower than LLM–human agreement for accuracy and empathy, with the senior ophthalmologist assigning stricter scores than the resident across all 3 outcomes. LLM-as-a-judge is thus best positioned as a scalable complement to, not a substitute for, human oversight.

These findings may have translational implications for patient education, where automatic readability alignment could help LLMs provide individualized explanations without relying on a single fixed reading target, particularly in populations with heterogeneous health literacy. Because the approach anchors response complexity in the user’s own language, it could also be explored in other domains that communicate with diverse audiences, such as education, public health messaging, and government services, although this was not evaluated here.

This approach is intended to support clinician-facing tools that generate readability-adapted responses for digital patient communication, with transparency regarding AI involvement, clinician responsibility, and mandatory clinician review before delivery; the experimental prompts reported in Multimedia Appendix 1 are not proposed unchanged for patient-facing deployment.

### Strengths and Limitations

This study has several strengths. Analysis across alternative readability formulas strengthened the robustness of our findings. Although agreement was highest with FRES, which served as the main control variable, the external calculator-guided strategy remained superior across FKGL, Gunning Fog, and even Dale-Chall, which relies on word familiarity rather than sentence and syllable length, suggesting that its advantage was not an artifact of a single formula. At the same time, lower kappa values with alternative indices reinforce that readability formulas capture different dimensions of textual complexity and that no single metric represents a gold standard for patient comprehension [2].

More broadly, our findings do not support relying on unconstrained model-based interpretation alone to control readability. Although natural language processing and machine learning approaches may complement traditional formulas, formula-free Semantic prompting performed substantially worse than externally guided prompting and, in some cases, worse than Baseline generation [30]. Traditional readability formulas, despite their limitations, remain transparent, reproducible, and operationally stable. Our findings therefore support a hybrid approach in which readability formulas serve as explicit control signals for LLM-generated patient communication.

Another strength was the use of real, unedited user questions rather than curated benchmarks or rewritten prompts, which better reflects real-world deployment and provides a stricter test of readability adaptation. The final sample was intentionally balanced across readability levels and question length to support robust comparisons, although basic and intermediate questions predominated in the original corpus.

This study also has several limitations. Readability formulas are indirect proxies for textual complexity and do not directly measure comprehension or broader health literacy, nor do they capture conceptual clarity, prior knowledge, discourse organization, or empathy [58,59]. Although preferred over grade-level formulas, FRES may be less stable for short or grammatically irregular text, in which sentence length and syllable counts dominate the score. Readability, understandability, and actionability are also distinct constructs [60]. Because individual health literacy, comprehension, and preferred explanation level were not assessed, question-response readability matching should be viewed as an operational objective rather than evidence that a matched response is optimal for a given patient. Evidence that readability adjustment alone improves comprehension is mixed [61-63]. Thus, our findings demonstrate structural readability control rather than improved comprehension or health literacy. Future studies should directly assess comprehension, recall, actionability, and decision-related outcomes.

The analysis was restricted to English-language questions, and the readability indices were originally developed for English; therefore, findings may not generalize fully to other languages. The questions were also unevenly distributed across ophthalmology topics, which may affect generalizability. Because the study was limited to ophthalmology, despite evidence that LLMs can simplify patient-facing materials in other specialties [9,15,16,19,43], these findings do not establish that question-response readability matching generalizes beyond ophthalmology. Further studies should evaluate other clinical domains and whether readability alignment can be achieved without compromising factual accuracy.

The use of YouTube comments also involves a trade-off because other public sources, including health forums and social media platforms, may represent different populations and question types. However, unedited YouTube comments preserve spontaneous language and resemble questions received by physicians on social media, a potential application of this pipeline. Recent, idiosyncratically worded comments may also be less likely to have appeared in model training, although this cannot be verified. Future studies should compare multiple question sources.

Future studies could prospectively recruit patients with diverse educational and health-literacy backgrounds, ask them to formulate questions in their own words, and obtain direct ratings of response readability, completeness, empathy, usefulness, and overall preference. Additional work should include objective measures of comprehension and recall, comparisons with fixed-target and general plain-language approaches, reproducibility across repeated generations, and generalizability across languages and clinical domains. Models explicitly trained or fine-tuned for readability estimation and controllable generation may also improve inference of question complexity and response alignment.

### Conclusions

The findings of this study suggest that LLM-generated responses can be aligned with the formula-derived readability of users’ own questions, particularly when externally calculated readability signals are used. Importantly, the findings underscore the roles of model architecture, readability-control strategy, and question characteristics in determining the readability and accuracy of LLM-generated responses.

## Supplementary material

10.2196/97408 Multimedia Appendix 1 Prompts used in each prompting strategy.

10.2196/97408 Multimedia Appendix 2 Pairwise comparisons of quadratic-weighted Cohen kappa with multiplicity adjustments.

10.2196/97408 Multimedia Appendix 3 Pairwise readability-tier contrasts with Holm-Bonferroni adjustment.

10.2196/97408 Multimedia Appendix 4 Stage-wise omnibus comparisons across question readability tiers for each model and pooled across models.

10.2196/97408 Multimedia Appendix 5 Examples of best and worst readability alignment by model and prompting strategy.

10.2196/97408 Multimedia Appendix 6 Examples of category-aligned question-response pairs across readability levels and models using the External Calculator–Guided Prompt strategy.

10.2196/97408 Multimedia Appendix 7 Accuracy analyses: pairwise comparisons across model-strategy configurations and associations of readability and question length with accuracy.

10.2196/97408 Multimedia Appendix 8 Pairwise comparisons of completeness scores with Holm adjustment.

10.2196/97408 Multimedia Appendix 9 Pairwise comparisons of empathy scores with Holm adjustment.

10.2196/97408 Multimedia Appendix 10 Mean evaluator ratings for accuracy, completeness, and empathy in the human-validation sample.

10.2196/97408 Multimedia Appendix 11 Pairwise comparisons of accuracy, completeness, and empathy scores among evaluators with Holm adjustment.

10.2196/97408 Multimedia Appendix 12 Computational efficiency of the readability-alignment strategies: descriptive statistics and pairwise comparisons.

10.2196/97408 Checklist 1 TRIPOD-LLM checklist.

## Abbreviations

BERTScore: Bidirectional Encoder Representations from Transformers Score
CCC: concordance correlation coefficient
FKGL: Flesch-Kincaid Grade Level
FRES: Flesch Reading Ease Score
LLM: large language model
MAE: mean absolute error
RMSE: root mean square error
RoBERTa: Robustly Optimized Bidirectional Encoder Representations from Transformers Pretraining Approach
