# Supplementary material for: Prompting Strategies for Readability Alignment Between User-Generated Ophthalmology Questions and Large Language Model Answers: Comparative Evaluation Study
Source: JMIR Form Res. 2026 Nov 5;10:e97408. doi: 10.2196/97408 (PMC13739205; doi:10.2196/97408)
Supplement: Supplementary file 2 [file formative-v10-e97408-s002.docx]

**Pairwise comparisons of quadratic-weighted Cohen kappa with multiplicity adjustments.**

| **Condition A** | **Condition B** | **κ A** | **κ B** | **Difference (κ A−κ B)** | ***P*raw** | ***P*Holm** | **Holm sig.** |
| --- | --- | --- | --- | --- | --- | --- | --- |
| Gemini 3 Textstat | GPT 5.2 Textstat | 0.9444 | 0.9029 | 0.0414 | .01 | .46 | No |
| Gemini 3 Textstat | OSS-20b Textstat | 0.9444 | 0.8756 | 0.0688 | <.001 | .02 | Yes |
| Gemini 3 Textstat | Gemini 3 FRES Prompt | 0.9444 | 0.7855 | 0.1589 | <.001 | .02 | Yes |
| Gemini 3 Textstat | Claude 4.5 Textstat | 0.9444 | 0.6911 | 0.2532 | <.001 | .02 | Yes |
| Gemini 3 Textstat | OSS-20b FRES Prompt | 0.9444 | 0.5931 | 0.3513 | <.001 | .02 | Yes |
| Gemini 3 Textstat | Claude 4.5 FRES Prompt | 0.9444 | 0.3445 | 0.5999 | <.001 | .02 | Yes |
| Gemini 3 Textstat | Claude 4.5 | 0.9444 | 0.2596 | 0.6848 | <.001 | .02 | Yes |
| Gemini 3 Textstat | GPT 5.2 | 0.9444 | 0.2474 | 0.6970 | <.001 | .02 | Yes |
| Gemini 3 Textstat | Gemini 3 | 0.9444 | 0.2224 | 0.7220 | <.001 | .02 | Yes |
| Gemini 3 Textstat | Claude 4.5 Semantic | 0.9444 | 0.2123 | 0.7321 | <.001 | .02 | Yes |
| Gemini 3 Textstat | OSS-20b | 0.9444 | 0.1627 | 0.7816 | <.001 | .02 | Yes |
| Gemini 3 Textstat | GPT 5.2 FRES Prompt | 0.9444 | 0.1302 | 0.8141 | <.001 | .02 | Yes |
| Gemini 3 Textstat | OSS-20b Semantic | 0.9444 | 0.1280 | 0.8163 | <.001 | .02 | Yes |
| Gemini 3 Textstat | GPT 5.2 Semantic | 0.9444 | 0.1233 | 0.8211 | <.001 | .02 | Yes |
| Gemini 3 Textstat | Gemini 3 Semantic | 0.9444 | 0.1032 | 0.8412 | <.001 | .02 | Yes |
| GPT 5.2 Textstat | OSS-20b Textstat | 0.9029 | 0.8756 | 0.0274 | .17 | >.99 | No |
| GPT 5.2 Textstat | Gemini 3 FRES Prompt | 0.9029 | 0.7855 | 0.1175 | <.001 | .02 | Yes |
| GPT 5.2 Textstat | Claude 4.5 Textstat | 0.9029 | 0.6911 | 0.2118 | <.001 | .02 | Yes |
| GPT 5.2 Textstat | OSS-20b FRES Prompt | 0.9029 | 0.5931 | 0.3099 | <.001 | .02 | Yes |
| GPT 5.2 Textstat | Claude 4.5 FRES Prompt | 0.9029 | 0.3445 | 0.5585 | <.001 | .02 | Yes |
| GPT 5.2 Textstat | Claude 4.5 | 0.9029 | 0.2596 | 0.6434 | <.001 | .02 | Yes |
| GPT 5.2 Textstat | GPT 5.2 | 0.9029 | 0.2474 | 0.6555 | <.001 | .02 | Yes |
| GPT 5.2 Textstat | Gemini 3 | 0.9029 | 0.2224 | 0.6805 | <.001 | .02 | Yes |
| GPT 5.2 Textstat | Claude 4.5 Semantic | 0.9029 | 0.2123 | 0.6906 | <.001 | .02 | Yes |
| GPT 5.2 Textstat | OSS-20b | 0.9029 | 0.1627 | 0.7402 | <.001 | .02 | Yes |
| GPT 5.2 Textstat | GPT 5.2 FRES Prompt | 0.9029 | 0.1302 | 0.7727 | <.001 | .02 | Yes |
| GPT 5.2 Textstat | OSS-20b Semantic | 0.9029 | 0.1280 | 0.7749 | <.001 | .02 | Yes |
| GPT 5.2 Textstat | GPT 5.2 Semantic | 0.9029 | 0.1233 | 0.7796 | <.001 | .02 | Yes |
| GPT 5.2 Textstat | Gemini 3 Semantic | 0.9029 | 0.1032 | 0.7997 | <.001 | .02 | Yes |
| OSS-20b Textstat | Gemini 3 FRES Prompt | 0.8756 | 0.7855 | 0.0901 | .001 | .053 | No |
| OSS-20b Textstat | Claude 4.5 Textstat | 0.8756 | 0.6911 | 0.1844 | <.001 | .02 | Yes |
| OSS-20b Textstat | OSS-20b FRES Prompt | 0.8756 | 0.5931 | 0.2825 | <.001 | .02 | Yes |
| OSS-20b Textstat | Claude 4.5 FRES Prompt | 0.8756 | 0.3445 | 0.5311 | <.001 | .02 | Yes |
| OSS-20b Textstat | Claude 4.5 | 0.8756 | 0.2596 | 0.6160 | <.001 | .02 | Yes |
| OSS-20b Textstat | GPT 5.2 | 0.8756 | 0.2474 | 0.6282 | <.001 | .02 | Yes |
| OSS-20b Textstat | Gemini 3 | 0.8756 | 0.2224 | 0.6532 | <.001 | .02 | Yes |
| OSS-20b Textstat | Claude 4.5 Semantic | 0.8756 | 0.2123 | 0.6633 | <.001 | .02 | Yes |
| OSS-20b Textstat | OSS-20b | 0.8756 | 0.1627 | 0.7128 | <.001 | .02 | Yes |
| OSS-20b Textstat | GPT 5.2 FRES Prompt | 0.8756 | 0.1302 | 0.7453 | <.001 | .02 | Yes |
| OSS-20b Textstat | OSS-20b Semantic | 0.8756 | 0.1280 | 0.7475 | <.001 | .02 | Yes |
| OSS-20b Textstat | GPT 5.2 Semantic | 0.8756 | 0.1233 | 0.7522 | <.001 | .02 | Yes |
| OSS-20b Textstat | Gemini 3 Semantic | 0.8756 | 0.1032 | 0.7723 | <.001 | .02 | Yes |
| Gemini 3 FRES Prompt | Claude 4.5 Textstat | 0.7855 | 0.6911 | 0.0943 | .004 | .17 | No |
| Gemini 3 FRES Prompt | OSS-20b FRES Prompt | 0.7855 | 0.5931 | 0.1924 | <.001 | .02 | Yes |
| Gemini 3 FRES Prompt | Claude 4.5 FRES Prompt | 0.7855 | 0.3445 | 0.4410 | <.001 | .02 | Yes |
| Gemini 3 FRES Prompt | Claude 4.5 | 0.7855 | 0.2596 | 0.5259 | <.001 | .02 | Yes |
| Gemini 3 FRES Prompt | GPT 5.2 | 0.7855 | 0.2474 | 0.5381 | <.001 | .02 | Yes |
| Gemini 3 FRES Prompt | Gemini 3 | 0.7855 | 0.2224 | 0.5631 | <.001 | .02 | Yes |
| Gemini 3 FRES Prompt | Claude 4.5 Semantic | 0.7855 | 0.2123 | 0.5732 | <.001 | .02 | Yes |
| Gemini 3 FRES Prompt | OSS-20b | 0.7855 | 0.1627 | 0.6227 | <.001 | .02 | Yes |
| Gemini 3 FRES Prompt | GPT 5.2 FRES Prompt | 0.7855 | 0.1302 | 0.6552 | <.001 | .02 | Yes |
| Gemini 3 FRES Prompt | OSS-20b Semantic | 0.7855 | 0.1280 | 0.6574 | <.001 | .02 | Yes |
| Gemini 3 FRES Prompt | GPT 5.2 Semantic | 0.7855 | 0.1233 | 0.6622 | <.001 | .02 | Yes |
| Gemini 3 FRES Prompt | Gemini 3 Semantic | 0.7855 | 0.1032 | 0.6822 | <.001 | .02 | Yes |
| Claude 4.5 Textstat | OSS-20b FRES Prompt | 0.6911 | 0.5931 | 0.0981 | <.001 | .02 | Yes |
| Claude 4.5 Textstat | Claude 4.5 FRES Prompt | 0.6911 | 0.3445 | 0.3467 | <.001 | .02 | Yes |
| Claude 4.5 Textstat | Claude 4.5 | 0.6911 | 0.2596 | 0.4316 | <.001 | .02 | Yes |
| Claude 4.5 Textstat | GPT 5.2 | 0.6911 | 0.2474 | 0.4437 | <.001 | .02 | Yes |
| Claude 4.5 Textstat | Gemini 3 | 0.6911 | 0.2224 | 0.4687 | <.001 | .02 | Yes |
| Claude 4.5 Textstat | Claude 4.5 Semantic | 0.6911 | 0.2123 | 0.4788 | <.001 | .02 | Yes |
| Claude 4.5 Textstat | OSS-20b | 0.6911 | 0.1627 | 0.5284 | <.001 | .02 | Yes |
| Claude 4.5 Textstat | GPT 5.2 FRES Prompt | 0.6911 | 0.1302 | 0.5609 | <.001 | .02 | Yes |
| Claude 4.5 Textstat | OSS-20b Semantic | 0.6911 | 0.1280 | 0.5631 | <.001 | .02 | Yes |
| Claude 4.5 Textstat | GPT 5.2 Semantic | 0.6911 | 0.1233 | 0.5678 | <.001 | .02 | Yes |
| Claude 4.5 Textstat | Gemini 3 Semantic | 0.6911 | 0.1032 | 0.5879 | <.001 | .02 | Yes |
| OSS-20b FRES Prompt | Claude 4.5 FRES Prompt | 0.5931 | 0.3445 | 0.2486 | <.001 | .02 | Yes |
| OSS-20b FRES Prompt | Claude 4.5 | 0.5931 | 0.2596 | 0.3335 | <.001 | .02 | Yes |
| OSS-20b FRES Prompt | GPT 5.2 | 0.5931 | 0.2474 | 0.3457 | <.001 | .02 | Yes |
| OSS-20b FRES Prompt | Gemini 3 | 0.5931 | 0.2224 | 0.3707 | <.001 | .02 | Yes |
| OSS-20b FRES Prompt | Claude 4.5 Semantic | 0.5931 | 0.2123 | 0.3808 | <.001 | .02 | Yes |
| OSS-20b FRES Prompt | OSS-20b | 0.5931 | 0.1627 | 0.4303 | <.001 | .02 | Yes |
| OSS-20b FRES Prompt | GPT 5.2 FRES Prompt | 0.5931 | 0.1302 | 0.4628 | <.001 | .02 | Yes |
| OSS-20b FRES Prompt | OSS-20b Semantic | 0.5931 | 0.1280 | 0.4650 | <.001 | .02 | Yes |
| OSS-20b FRES Prompt | GPT 5.2 Semantic | 0.5931 | 0.1233 | 0.4698 | <.001 | .02 | Yes |
| OSS-20b FRES Prompt | Gemini 3 Semantic | 0.5931 | 0.1032 | 0.4898 | <.001 | .02 | Yes |
| Claude 4.5 FRES Prompt | Claude 4.5 | 0.3445 | 0.2596 | 0.0849 | .07 | >.99 | No |
| Claude 4.5 FRES Prompt | GPT 5.2 | 0.3445 | 0.2474 | 0.0971 | .04 | >.99 | No |
| Claude 4.5 FRES Prompt | Gemini 3 | 0.3445 | 0.2224 | 0.1221 | .01 | .38 | No |
| Claude 4.5 FRES Prompt | Claude 4.5 Semantic | 0.3445 | 0.2123 | 0.1322 | .002 | .10 | No |
| Claude 4.5 FRES Prompt | OSS-20b | 0.3445 | 0.1627 | 0.1817 | <.001 | .04 | Yes |
| Claude 4.5 FRES Prompt | GPT 5.2 FRES Prompt | 0.3445 | 0.1302 | 0.2142 | <.001 | .02 | Yes |
| Claude 4.5 FRES Prompt | OSS-20b Semantic | 0.3445 | 0.1280 | 0.2164 | <.001 | .02 | Yes |
| Claude 4.5 FRES Prompt | GPT 5.2 Semantic | 0.3445 | 0.1233 | 0.2211 | <.001 | .02 | Yes |
| Claude 4.5 FRES Prompt | Gemini 3 Semantic | 0.3445 | 0.1032 | 0.2412 | <.001 | .02 | Yes |
| Claude 4.5 | GPT 5.2 | 0.2596 | 0.2474 | 0.0122 | .76 | >.99 | No |
| Claude 4.5 | Gemini 3 | 0.2596 | 0.2224 | 0.0372 | .39 | >.99 | No |
| Claude 4.5 | Claude 4.5 Semantic | 0.2596 | 0.2123 | 0.0473 | .24 | >.99 | No |
| Claude 4.5 | OSS-20b | 0.2596 | 0.1627 | 0.0968 | .03 | .78 | No |
| Claude 4.5 | GPT 5.2 FRES Prompt | 0.2596 | 0.1302 | 0.1293 | .04 | >.99 | No |
| Claude 4.5 | OSS-20b Semantic | 0.2596 | 0.1280 | 0.1315 | .01 | .38 | No |
| Claude 4.5 | GPT 5.2 Semantic | 0.2596 | 0.1233 | 0.1362 | .004 | .15 | No |
| Claude 4.5 | Gemini 3 Semantic | 0.2596 | 0.1032 | 0.1563 | .002 | .09 | No |
| GPT 5.2 | Gemini 3 | 0.2474 | 0.2224 | 0.0250 | .53 | >.99 | No |
| GPT 5.2 | Claude 4.5 Semantic | 0.2474 | 0.2123 | 0.0351 | .40 | >.99 | No |
| GPT 5.2 | OSS-20b | 0.2474 | 0.1627 | 0.0846 | .05 | >.99 | No |
| GPT 5.2 | GPT 5.2 FRES Prompt | 0.2474 | 0.1302 | 0.1171 | .04 | >.99 | No |
| GPT 5.2 | OSS-20b Semantic | 0.2474 | 0.1280 | 0.1194 | .007 | .25 | No |
| GPT 5.2 | GPT 5.2 Semantic | 0.2474 | 0.1233 | 0.1241 | .002 | .08 | No |
| GPT 5.2 | Gemini 3 Semantic | 0.2474 | 0.1032 | 0.1442 | .003 | .11 | No |
| Gemini 3 | Claude 4.5 Semantic | 0.2224 | 0.2123 | 0.0101 | .84 | >.99 | No |
| Gemini 3 | OSS-20b | 0.2224 | 0.1627 | 0.0596 | .15 | >.99 | No |
| Gemini 3 | GPT 5.2 FRES Prompt | 0.2224 | 0.1302 | 0.0921 | .11 | >.99 | No |
| Gemini 3 | OSS-20b Semantic | 0.2224 | 0.1280 | 0.0943 | .04 | >.99 | No |
| Gemini 3 | GPT 5.2 Semantic | 0.2224 | 0.1233 | 0.0991 | .02 | .69 | No |
| Gemini 3 | Gemini 3 Semantic | 0.2224 | 0.1032 | 0.1192 | .03 | .79 | No |
| Claude 4.5 Semantic | OSS-20b | 0.2123 | 0.1627 | 0.0495 | .32 | >.99 | No |
| Claude 4.5 Semantic | GPT 5.2 FRES Prompt | 0.2123 | 0.1302 | 0.0821 | .17 | >.99 | No |
| Claude 4.5 Semantic | OSS-20b Semantic | 0.2123 | 0.1280 | 0.0843 | .11 | >.99 | No |
| Claude 4.5 Semantic | GPT 5.2 Semantic | 0.2123 | 0.1233 | 0.0890 | .045 | >.99 | No |
| Claude 4.5 Semantic | Gemini 3 Semantic | 0.2123 | 0.1032 | 0.1091 | .006 | .24 | No |
| OSS-20b | GPT 5.2 FRES Prompt | 0.1627 | 0.1302 | 0.0325 | .56 | >.99 | No |
| OSS-20b | OSS-20b Semantic | 0.1627 | 0.1280 | 0.0347 | .44 | >.99 | No |
| OSS-20b | GPT 5.2 Semantic | 0.1627 | 0.1233 | 0.0394 | .37 | >.99 | No |
| OSS-20b | Gemini 3 Semantic | 0.1627 | 0.1032 | 0.0595 | .27 | >.99 | No |
| GPT 5.2 FRES Prompt | OSS-20b Semantic | 0.1302 | 0.1280 | 0.0022 | .96 | >.99 | No |
| GPT 5.2 FRES Prompt | GPT 5.2 Semantic | 0.1302 | 0.1233 | 0.0069 | .90 | >.99 | No |
| GPT 5.2 FRES Prompt | Gemini 3 Semantic | 0.1302 | 0.1032 | 0.0270 | .64 | >.99 | No |
| OSS-20b Semantic | GPT 5.2 Semantic | 0.1280 | 0.1233 | 0.0047 | .91 | >.99 | No |
| OSS-20b Semantic | Gemini 3 Semantic | 0.1280 | 0.1032 | 0.0248 | .63 | >.99 | No |
| GPT 5.2 Semantic | Gemini 3 Semantic | 0.1233 | 0.1032 | 0.0201 | .66 | >.99 | No |

For each model–strategy configuration, quadratic-weighted Cohen kappa (κ) quantified agreement between the readability category of the original question and that of the generated response across 400 paired question–response observations. Differences were calculated as κA−κB using unrounded κ values. Pairwise comparisons used 2-sided paired permutation tests with 5000 valid permutations. P values were adjusted across all 120 comparisons using the Holm procedure (Holm-Bonferroni correction). P values are reported to 2 decimal places, except when P<.01 or when rounding would alter the interpretation of statistical significance. Values below .001 are reported as P<.001, and values above .99 as P>.99. Kappa coefficients and differences are reported to 4 decimal places. Statistical significance was defined as Holm-adjusted P<.05. Model names shown without a strategy label represent the Baseline condition. Praw indicates the unadjusted P value; PHolm, the Holm-adjusted P value; and Holm sig., statistical significance after Holm correction.
