# Supplementary material for: Prompting Strategies for Readability Alignment Between User-Generated Ophthalmology Questions and Large Language Model Answers: Comparative Evaluation Study
Source: JMIR Form Res. 2026 Nov 5;10:e97408. doi: 10.2196/97408 (PMC13739205; doi:10.2196/97408)
Supplement: Supplementary file 3 [file formative-v10-e97408-s003.docx]

**Pairwise tier contrasts with Holm-Bonferroni adjustment.**

| **Model/Question** | **Strategy** | **Comparison** | **Test** | **t(df)** | ***P*_raw_** | ***P*_Holm_** | **Hedges g** | **Holm sig.** |
| --- | --- | --- | --- | --- | --- | --- | --- | --- |
| **Question** | - | Basic vs Intermediate | Welch t | 21.47 (204.2) | <.001 | <.001 | 2.671 | Yes |
|  |  | Basic vs Higher |  | 55.83 (166.2) | <.001 | <.001 | 7.445 | Yes |
|  |  | Intermediate vs Higher |  | 30.93 (228.5) | <.001 | <.001 | 4.007 | Yes |
| **GPT 5.2** | Baseline | Basic vs Intermediate | Student t | 3.18 (285.0) | .002 | .002 | 0.377 | Yes |
|  |  | Basic vs Higher |  | 8.84 (271.0) | <.001 | <.001 | 1.083 | Yes |
|  |  | Intermediate vs Higher |  | 4.83 (238.0) | <.001 | <.001 | 0.622 | Yes |
|  | Semantic | Basic vs Intermediate | Student t | 3.21 (285.0) | .001 | .003 | 0.381 | Yes |
|  |  | Basic vs Higher |  | 4.73 (271.0) | <.001 | <.001 | 0.580 | Yes |
|  |  | Intermediate vs Higher |  | 1.25 (238.0) | .21 | .21 | 0.161 | No |
|  | FRES Prompt | Basic vs Intermediate | Student t | 4.39 (285.0) | <.001 | <.001 | 0.520 | Yes |
|  |  | Basic vs Higher |  | 5.25 (271.0) | <.001 | <.001 | 0.643 | Yes |
|  |  | Intermediate vs Higher |  | 0.79 (238.0) | .43 | .43 | 0.102 | No |
|  | Textstat | Basic vs Intermediate | Welch t | 26.13 (202.4) | <.001 | <.001 | 3.255 | Yes |
|  |  | Basic vs Higher |  | 51.93 (194.7) | <.001 | <.001 | 6.695 | Yes |
|  |  | Intermediate vs Higher |  | 18.84 (237.6) | <.001 | <.001 | 2.406 | Yes |
| **Gemini 3** | Baseline | Basic vs Intermediate | Student t | 4.00 (285.0) | <.001 | <.001 | 0.474 | Yes |
|  |  | Basic vs Higher |  | 7.87 (271.0) | <.001 | <.001 | 0.965 | Yes |
|  |  | Intermediate vs Higher |  | 3.67 (238.0) | <.001 | <.001 | 0.473 | Yes |
|  | Semantic | Basic vs Intermediate | Student t | 3.24 (285.0) | .001 | .003 | 0.384 | Yes |
|  |  | Basic vs Higher |  | 3.93 (271.0) | <.001 | <.001 | 0.481 | Yes |
|  |  | Intermediate vs Higher |  | 0.59 (238.0) | .56 | .56 | 0.076 | No |
|  | FRES Prompt | Basic vs Intermediate | Welch t | 15.70 (273.4) | <.001 | <.001 | 1.855 | Yes |
|  |  | Basic vs Higher |  | 21.53 (170.3) | <.001 | <.001 | 2.856 | Yes |
|  |  | Intermediate vs Higher |  | 10.71 (177.8) | <.001 | <.001 | 1.420 | Yes |
|  | Textstat | Basic vs Intermediate | Welch t | 38.67 (238.7) | <.001 | <.001 | 4.698 | Yes |
|  |  | Basic vs Higher |  | 71.34 (197.4) | <.001 | <.001 | 9.170 | Yes |
|  |  | Intermediate vs Higher |  | 31.78 (230.3) | <.001 | <.001 | 4.112 | Yes |
| **Claude 4.5** | Baseline | Basic vs Intermediate | Welch t | 5.06 (242.3) | <.001 | <.001 | 0.613 | Yes |
|  |  | Basic vs Higher |  | 9.75 (210.0) | <.001 | <.001 | 1.236 | Yes |
|  |  | Intermediate vs Higher |  | 4.33 (234.5) | <.001 | <.001 | 0.559 | Yes |
|  | Semantic | Basic vs Intermediate | Student t | 3.61 (285.0) | <.001 | <.001 | 0.428 | Yes |
|  |  | Basic vs Higher |  | 5.99 (271.0) | <.001 | <.001 | 0.734 | Yes |
|  |  | Intermediate vs Higher |  | 2.47 (238.0) | .01 | .01 | 0.319 | Yes |
|  | FRES Prompt | Basic vs Intermediate | Welch t | 7.27 (237.6) | <.001 | <.001 | 0.884 | Yes |
|  |  | Basic vs Higher |  | 8.16 (169.8) | <.001 | <.001 | 1.083 | Yes |
|  |  | Intermediate vs Higher |  | 2.25 (207.9) | .03 | .03 | 0.294 | Yes |
|  | Textstat | Basic vs Intermediate | Student t | 24.28 (285.0) | <.001 | <.001 | 2.877 | Yes |
|  |  | Basic vs Higher |  | 40.04 (271.0) | <.001 | <.001 | 4.907 | Yes |
|  |  | Intermediate vs Higher |  | 14.72 (238.0) | <.001 | <.001 | 1.897 | Yes |
| **OSS-20b** | Baseline | Basic vs Intermediate | Student t | 3.67 (285.0) | <.001 | <.001 | 0.434 | Yes |
|  |  | Basic vs Higher |  | 7.17 (271.0) | <.001 | <.001 | 0.879 | Yes |
|  |  | Intermediate vs Higher |  | 3.48 (238.0) | <.001 | <.001 | 0.449 | Yes |
|  | Semantic | Basic vs Intermediate | Student t | 1.83 (285.0) | .07 | .07 | 0.216 | No |
|  |  | Basic vs Higher |  | 4.43 (271.0) | <.001 | <.001 | 0.543 | Yes |
|  |  | Intermediate vs Higher |  | 2.40 (238.0) | .02 | .03 | 0.309 | Yes |
|  | FRES Prompt | Basic vs Intermediate | Student t | 7.25 (285.0) | <.001 | <.001 | 0.859 | Yes |
|  |  | Basic vs Higher |  | 20.65 (271.0) | <.001 | <.001 | 2.531 | Yes |
|  |  | Intermediate vs Higher |  | 12.07 (238.0) | <.001 | <.001 | 1.556 | Yes |
|  | Textstat | Basic vs Intermediate | Welch t | 24.16 (219.3) | <.001 | <.001 | 2.975 | Yes |
|  |  | Basic vs Higher |  | 46.13 (163.6) | <.001 | <.001 | 6.171 | Yes |
|  |  | Intermediate vs Higher |  | 23.45 (215.0) | <.001 | <.001 | 3.061 | Yes |
| **All models (pooled)** | Baseline | Basic vs Intermediate | Welch t | 7.59 (1022.9) | <.001 | <.001 | 0.457 | Yes |
|  |  | Basic vs Higher |  | 16.02 (907.5) | <.001 | <.001 | 1.001 | Yes |
|  |  | Intermediate vs Higher |  | 7.82 (948.3) | <.001 | <.001 | 0.505 | Yes |
|  | Semantic | Basic vs Intermediate | Welch t | 5.77 (1049.0) | <.001 | <.001 | 0.346 | Yes |
|  |  | Basic vs Higher |  | 8.88 (933.0) | <.001 | <.001 | 0.551 | Yes |
|  |  | Intermediate vs Higher |  | 3.02 (947.5) | .003 | .003 | 0.195 | Yes |
|  | FRES Prompt | Basic vs Intermediate | Welch t | 15.80 (1052.3) | <.001 | <.001 | 0.945 | Yes |
|  |  | Basic vs Higher |  | 23.59 (722.4) | <.001 | <.001 | 1.550 | Yes |
|  |  | Intermediate vs Higher |  | 11.06 (799.5) | <.001 | <.001 | 0.729 | Yes |
|  | Textstat | Basic vs Intermediate | Welch t | 48.45 (972.5) | <.001 | <.001 | 2.943 | Yes |
|  |  | Basic vs Higher |  | 91.00 (837.1) | <.001 | <.001 | 5.793 | Yes |
|  |  | Intermediate vs Higher |  | 39.71 (941.7) | <.001 | <.001 | 2.568 | Yes |

Hedges g represents the pairwise standardized mean difference. The Student t test was used when the equal-variance assumption was met; otherwise, the Welch t test was used. P values are shown exactly unless P<.001. Statistical significance was defined as Holm-adjusted P<.05. Praw indicates the unadjusted P value; PHolm, the Holm-adjusted P value; and Holm sig., statistical significance after Holm correction. Abbreviation: df, degrees of freedom. P values were adjusted separately for the 3 pairwise readability-tier contrasts within each model–strategy configuration and for the original questions using the Holm procedure.
