# Supplementary material for: Prompting Strategies for Readability Alignment Between User-Generated Ophthalmology Questions and Large Language Model Answers: Comparative Evaluation Study
Source: JMIR Form Res. 2026 Nov 5;10:e97408. doi: 10.2196/97408 (PMC13739205; doi:10.2196/97408)
Supplement: Supplementary file 4 [file formative-v10-e97408-s004.docx]

**Stage-wise comparisons using omnibus one-way ANOVA across question tiers (Basic, Intermediate, Higher) for each model and pooled across models.**

| **Model/Question** | **Strategy** | **n (B/I/H)** | **Basic mean±SD** | **Intermediate mean±SD** | **Higher mean±SD** | **F(df1, df2)** | ***P*** | **ω^2^** |
| --- | --- | --- | --- | --- | --- | --- | --- | --- |
| **Question** | - | 160/127/113 | 78.21±6.37 | 56.38±9.95 | 14.63±10.86 | 1664.76 (2, 397) | <.001 | 0.893 |
| **GPT 5.2** | Baseline | 160/127/113 | 40.82±10.29 | 36.68±11.75 | 29.93±9.64 | 34.97 (2, 397) | <.001 | 0.145 |
|  | Semantic | 160/127/113 | 43.47±15.32 | 37.42±16.50 | 34.97±13.57 | 11.50 (2, 397) | <.001 | 0.050 |
|  | FRES Prompt | 160/127/113 | 58.41±12.01 | 51.75±13.69 | 50.38±13.09 | 15.83 (2, 397) | <.001 | 0.069 |
|  | Textstat | 160/127/113 | 76.74±6.82 | 48.02±10.79 | 23.68±9.23 | 1195.92 (2, 397) | <.001 | 0.857 |
| **Gemini 3** | Baseline | 160/127/113 | 40.60±7.96 | 36.74±8.31 | 32.84±8.09 | 30.56 (2, 397) | <.001 | 0.129 |
|  | Semantic | 160/127/113 | 48.00±21.29 | 39.38±23.72 | 37.66±21.63 | 8.85 (2, 397) | <.001 | 0.038 |
|  | FRES Prompt | 160/127/113 | 71.90±13.27 | 47.51±12.92 | 22.39±21.75 | 318.49 (2, 397) | <.001 | 0.614 |
|  | Textstat | 160/127/113 | 76.09±5.97 | 44.75±7.43 | 13.13±7.93 | 2676.10 (2, 397) | <.001 | 0.930 |
| **Claude 4.5** | Baseline | 160/127/113 | 36.59±8.99 | 30.51±10.94 | 24.37±10.98 | 47.85 (2, 397) | <.001 | 0.190 |
|  | Semantic | 160/127/113 | 38.47±24.54 | 28.12±23.59 | 20.45±24.42 | 18.95 (2, 397) | <.001 | 0.082 |
|  | FRES Prompt | 160/127/113 | 55.89±15.91 | 40.13±19.92 | 33.31±26.20 | 44.33 (2, 397) | <.001 | 0.178 |
|  | Textstat | 160/127/113 | 64.85±8.52 | 38.27±10.02 | 18.83±10.42 | 792.55 (2, 397) | <.001 | 0.798 |
| **OSS-20b** | Baseline | 160/127/113 | 42.32±9.94 | 37.93±10.24 | 33.14±11.05 | 26.13 (2, 397) | <.001 | 0.112 |
|  | Semantic | 160/127/113 | 49.90±12.59 | 47.02±14.06 | 42.60±14.49 | 9.51 (2, 397) | <.001 | 0.041 |
|  | FRES Prompt | 160/127/113 | 57.21±14.06 | 44.40±15.85 | 19.78±15.67 | 204.62 (2, 397) | <.001 | 0.504 |
|  | Textstat | 160/127/113 | 72.15±6.44 | 49.16±9.06 | 18.01±11.24 | 1239.72 (2, 397) | <.001 | 0.861 |
| **All models (pooled)** | Baseline | 640/508/452 | 40.08±9.56 | 35.46±10.76 | 30.07±10.59 | 126.92 (2, 1597) | <.001 | 0.136 |
|  | Semantic | 640/508/452 | 44.96±19.49 | 37.99±20.98 | 33.92±20.74 | 41.47 (2, 1597) | <.001 | 0.048 |
|  | FRES Prompt | 640/508/452 | 60.85±15.28 | 45.95±16.35 | 31.47±23.17 | 350.78 (2, 1597) | <.001 | 0.304 |
|  | Textstat | 640/508/452 | 72.46±8.44 | 45.05±10.30 | 18.41±10.45 | 4208.48 (2, 1597) | <.001 | 0.840 |

One-way ANOVA was used as the omnibus test. Variance heterogeneity was addressed in pairwise analyses using Welch’s t test when appropriate. P values are shown exactly unless P<.001. Omega squared (ω²) represents the omnibus effect size. Abbreviations: B, Basic; H, Higher; I, Intermediate; SD, standard deviation.
