# Supplementary material for: Prompting Strategies for Readability Alignment Between User-Generated Ophthalmology Questions and Large Language Model Answers: Comparative Evaluation Study
Source: JMIR Form Res. 2026 Nov 5;10:e97408. doi: 10.2196/97408 (PMC13739205; doi:10.2196/97408)
Supplement: Supplementary file 6 [file formative-v10-e97408-s006.docx]

**Examples of Category-Aligned Question–Response Pairs Across Readability Levels and Large Language Models Using the External Calculator–Guided Prompt Strategy**

# Claude 4.5

**Basic level**

| **Question** | What should I do if one of my own hairs has gotten into my eye? There is a hair in my right eye. A doctor said it was only a floater, but I know what my floaters look like, and this is obviously a hair in my line of vision. What are my options? |
| --- | --- |
| **Original Question Readability** | FRES: 89.78; Question category: Basic |
| **Generated Response** | I understand how frustrating this must be! Seeing something that looks like a hair in your vision can be really bothersome.  Here's the thing: a loose hair sitting on the surface of your eye would cause irritation, redness, and tearing. Your eye would try to wash it out naturally. You'd feel it when you blink.  But if you see something hair-like that moves when your eye moves and doesn't cause pain or irritation, it's likely inside the eye itself. These are floaters, which are tiny strands floating in the gel inside your eye. They can look exactly like a hair or thread.  **What you can do:**  - If your eye feels irritated, try rinsing with clean water or artificial tears  - If there's no irritation but you still see it, a second opinion from a retina specialist might help clarify things  This doesn't replace a full eye exam, but I hope this helps explain what might be happening! |
| **Response Readability** | FRES: 71.56; Category: Basic |
| **Factual Accuracy (LLM-as-a-Judge)** | 1 (Accurate) |

**Intermediate level**

| **Question** | I have very high myopia and cannot tolerate contact lenses. After an eye evaluation, clear lens exchange was suggested as an option. Is this considered an excessive procedure, and does replacing the natural lens affect the focusing muscles or create other long-term risks? |
| --- | --- |
| **Original Question Readability** | FRES: 51.18; Question category: Intermediate |
| **Generated Response** | Thank you for reaching out with such a thoughtful question.  I understand your concern about whether CLE (Clear Lens Exchange) seems extreme. Given your high myopia of -16 diopters and contact lens intolerance, CLE is actually a well-established option when laser procedures aren't suitable. Many patients in similar situations have undergone this successfully.  Regarding the muscles you mentioned, I believe you're referring to the ciliary muscle, which controls focusing (accommodation). After lens replacement, this muscle becomes less active since the artificial lens typically has a fixed focus. This is a normal, expected outcome, not a complication. Most patients adapt well, though you may need reading glasses depending on the lens type chosen.  This isn't necessarily extreme; it's about finding the right solution for your specific eyes.  I'd encourage you to discuss all your concerns thoroughly with your surgeon, including lens options like multifocal or extended-depth lenses that might reduce glasses dependence.  Is there anything specific about the procedure you'd like me to clarify? |
| **Response Readability** | FRES: 42.84; Category: Intermediate |
| **Factual Accuracy (LLM-as-a-Judge)** | 1 (Accurate) |

**Higher level**

| **Question** | Are there any nonsurgical ways to manage keratoconus, such as vitamin supplements or other natural approaches? |
| --- | --- |
| **Original Question Readability** | FRES: 15.40; Question category: Higher |
| **Generated Response** | Thank you for reaching out about keratoconus management.  I understand the desire to explore non-surgical options. However, I must be transparent with you: keratoconus is a progressive corneal condition where the cornea thins and bulges, and currently there are no vitamins or natural treatments scientifically proven to halt or reverse its progression.  **What can help manage the condition:**  - **Rigid gas permeable (RGP) contact lenses** or **scleral lenses** are the primary non-surgical approach to improve vision by creating a smooth optical surface over the irregular cornea.  - **Hybrid lenses** may also be an option for comfort and clarity.  **Regarding supplements:** While general eye health benefits from nutrients like vitamin A, C, E, and omega-3 fatty acids, none have demonstrated effectiveness specifically for keratoconus.  **Collagen cross-linking** is a minimally invasive procedure (not traditional surgery) that can slow progression and is worth discussing with your ophthalmologist.  I strongly recommend scheduling a consultation with a cornea specialist to discuss your specific stage and appropriate management options. |
| **Response Readability** | FRES: 18.78; Category: Higher |
| **Factual Accuracy (LLM-as-a-Judge)** | 1 (Accurate) |

# Gemini 3

**Basic level**

| **Question** | What should I do if one of my own hairs has gotten into my eye? There is a hair in my right eye. A doctor said it was only a floater, but I know what my floaters look like, and this is obviously a hair in my line of vision. What are my options? |
| --- | --- |
| **Original Question Readability** | FRES: 89.78; Question category: Basic |
| **Generated Response** | I understand how annoying and frustrating this is for you. It can be very hard to tell the difference between a real hair and a floater.  Floaters are tiny clumps in the jelly inside your eye. When light hits them, they make a shadow. To you, this shadow looks exactly like a hair or a thread floating in water. If you see the shape moving but your eye does not hurt or feel scratchy, it is likely inside the eye.  If your eye feels like something is scratching the surface, try using plain artificial tears (drops) to rinse it. If the shape changes, you see flashing lights, or it keeps bothering you, please schedule a visit with an eye doctor to be sure.  This message does not replace a real medical consultation. |
| **Response Readability** | FRES: 77.87; Category: Basic |
| **Factual Accuracy (LLM-as-a-Judge)** | 1 (Accurate) |

**Intermediate level**

| **Question** | I have very high myopia and cannot tolerate contact lenses. After an eye evaluation, clear lens exchange was suggested as an option. Is this considered an excessive procedure, and does replacing the natural lens affect the focusing muscles or create other long-term risks? |
| --- | --- |
| **Original Question Readability** | FRES: 51.18; Question category: Intermediate |
| **Generated Response** | Hello. It is completely normal to have questions before a major procedure. Clear Lens Exchange (CLE) involves replacing your natural lens with an artificial one. While it is an invasive surgery, it uses the same proven techniques as cataract surgery, which is performed very frequently. For someone with a -16 prescription, it is often considered when laser vision correction is not safe.  Regarding the muscles, the ciliary muscles inside your eye do not stop working or atrophy. However, the artificial lens cannot change shape like your natural lens does. This means you lose the natural ability to focus up close (accommodation), often requiring reading glasses or multifocal implants. This loss of focus is expected, not a complication that damages the eye.  Because you have high myopia, your retina is more fragile. Intraocular surgery does increase the risk of retinal detachment, so discussing this specific risk with your surgeon is vital.  Please remember this information does not replace a medical consultation. |
| **Response Readability** | FRES: 42.44; Category: Intermediate |
| **Factual Accuracy (LLM-as-a-Judge)** | 1 (Accurate) |

**Higher level**

| **Question** | Are there any nonsurgical ways to manage keratoconus, such as vitamin supplements or other natural approaches? |
| --- | --- |
| **Original Question Readability** | FRES: 15.40; Question category: Higher |
| **Generated Response** | It is understandable that you wish to manage keratoconus conservatively. However, it is important to clarify that there are currently no scientifically proven natural treatments or vitamin regimens capable of reversing the corneal thinning and ectasia characteristic of this disease. While systemic nutrition supports general ocular health, it cannot structurally reinforce the cornea.  The most critical lifestyle modification you must implement is the absolute cessation of eye rubbing, as mechanical trauma significantly accelerates progression. Non-surgical management primarily focuses on visual rehabilitation using specialty contact lenses, such as rigid gas permeable or scleral lenses. While these improve vision, they do not halt disease progression. Corneal cross-linking is often required to stabilize the cornea and prevent the need for future transplantation. Please consult your ophthalmologist to monitor your corneal topography closely.  This information is for educational purposes and does not replace a professional medical consultation. |
| **Response Readability** | FRES: 6.13; Category: Higher |
| **Factual Accuracy (LLM-as-a-Judge)** | 1 (Accurate) |

# GPT 5.2

**Basic level**

| **Question** | What should I do if one of my own hairs has gotten into my eye? There is a hair in my right eye. A doctor said it was only a floater, but I know what my floaters look like, and this is obviously a hair in my line of vision. What are my options? |
| --- | --- |
| **Original Question Readability** | FRES: 89.78; Question category: Basic |
| **Generated Response** | A real hair cannot usually get inside the eyeball. It can sit on the surface (like on the clear front window of the eye) or under the lid. That can feel scratchy, watery, and red. But it usually does not look like a sharp “hair” in your vision all the time.  A floater is a tiny strand inside the eye gel. It can look like a hair or thread and moves when you move your eye, like seaweed in water.  What you can do now: wash hands, do not rub, blink a lot, and rinse the eye with clean saline or clean water. You can gently pull the upper lid down over the lower lid and blink.  If it still feels stuck or you still see it, book an eye exam. This does not replace a medical visit. |
| **Response Readability** | FRES: 88.61; Category: Basic |
| **Factual Accuracy (LLM-as-a-Judge)** | 1 (Accurate) |

**Intermediate level**

| **Question** | I have very high myopia and cannot tolerate contact lenses. After an eye evaluation, clear lens exchange was suggested as an option. Is this considered an excessive procedure, and does replacing the natural lens affect the focusing muscles or create other long-term risks? |
| --- | --- |
| **Original Question Readability** | FRES: 51.18; Question category: Intermediate |
| **Generated Response** | Clear lens extraction (CLE), also called refractive lens exchange, can be an option for very high myopia like -16 when laser surgery is not suitable. It is a bigger step than corneal laser because it removes your natural lens, so it is usually considered “more invasive,” especially in younger people.  About “muscles not being active”: the eye muscles that move the eye still work normally. What you lose is accommodation, meaning the lens’ ability to change focus for near vision. After CLE, you will likely need reading glasses (or special lens choices may reduce this, but not perfectly).  Key risks to discuss include retinal detachment (higher in high myopia), glare/halos, infection, and needing future procedures.  This does not replace an in-person consultation; a retina risk discussion is important before deciding. |
| **Response Readability** | FRES: 42.86; Category: Intermediate |
| **Factual Accuracy (LLM-as-a-Judge)** | 1 (Accurate) |

**Higher level**

| **Question** | Are there any nonsurgical ways to manage keratoconus, such as vitamin supplements or other natural approaches? |
| --- | --- |
| **Original Question Readability** | FRES: 15.40; Question category: Higher |
| **Generated Response** | Keratoconus is a progressive thinning and bulging of the cornea. Unfortunately, vitamins or “natural” therapies have not been shown to stop or reverse it. A generally healthy diet and avoiding smoking support overall eye health, but they do not correct the corneal shape.  The most helpful non-surgical steps are protective and mechanical: avoid eye rubbing (it can accelerate progression), treat allergies and dry eye to reduce rubbing triggers, and use appropriate vision correction such as glasses early on or specialty contact lenses (rigid gas permeable, hybrid, or scleral lenses) to improve vision.  Some people ask about antioxidants (vitamin C, E, omega-3); they are reasonable for general health but should not be relied on as treatment.  This information does not replace an in-person ophthalmology consultation; regular follow-up is important to monitor progression and discuss options. |
| **Response Readability** | FRES: 25.70; Category: Higher |
| **Factual Accuracy (LLM-as-a-Judge)** | 1 (Accurate) |

# OSS-20b

**Basic level**

| **Question** | What should I do if one of my own hairs has gotten into my eye? There is a hair in my right eye. A doctor said it was only a floater, but I know what my floaters look like, and this is obviously a hair in my line of vision. What are my options? |
| --- | --- |
| **Original Question Readability** | FRES: 89.78; Question category: Basic |
| **Generated Response** | If a hair is in your eye, it can feel itchy or make your vision look a bit fuzzy. First, do not rub your eye, because rubbing can push the hair deeper or scratch the eye. Try rinsing the eye with clean, lukewarm water or a sterile saline solution. Hold your eyelid open and let the water run over the eye for a few seconds. You can also use a gentle artificial‑tear drop to help wash the hair out. If the hair stays, gently look at a bright light and blink a few times; sometimes the blinking motion can help the hair fall out. If the hair does not come out or you feel pain, redness, or your vision changes, please see an eye doctor soon. This will keep your eye safe and clear. |
| **Response Readability** | FRES: 85.03; Category: Basic |
| **Factual Accuracy (LLM-as-a-Judge)** | 1 (Accurate) |

**Intermediate level**

| **Question** | I have very high myopia and cannot tolerate contact lenses. After an eye evaluation, clear lens exchange was suggested as an option. Is this considered an excessive procedure, and does replacing the natural lens affect the focusing muscles or create other long-term risks? |
| --- | --- |
| **Original Question Readability** | FRES: 51.18; Question category: Intermediate |
| **Generated Response** | I’m glad you’re asking about this. Refractive lens exchange (sometimes called CLE) is a well‑established surgery that replaces the eye’s natural lens with an artificial one to correct refractive errors like high myopia. It is not considered “extreme” for most patients, but it is a surgical procedure, so it carries the usual risks of any eye surgery—such as infection, inflammation, or changes in vision. The eye’s extra‑ocular muscles that move the eye are not removed or damaged, so eye‑movement control remains normal. Some people experience a slight change in how the eye focuses after the lens is replaced, but this is usually managed with glasses or contact lenses. Discuss the specific risks and benefits with your surgeon; they can tailor the plan to your eye health and lifestyle. |
| **Response Readability** | FRES: 49.69; Category: Intermediate |
| **Factual Accuracy (LLM-as-a-Judge)** | 0 (Inaccurate) |

**Higher level**

| **Question** | Are there any nonsurgical ways to manage keratoconus, such as vitamin supplements or other natural approaches? |
| --- | --- |
| **Original Question Readability** | FRES: 15.40; Question category: Higher |
| **Generated Response** | Keratoconus is a progressive corneal ectasia for which no natural therapy can reverse the structural changes. Supportive measures can help slow progression and improve comfort. Adequate hydration, avoidance of eye rubbing, and protection from UV light are foundational. Nutritional support—particularly antioxidants such as vitamin C, vitamin E, and omega‑3 fatty acids—may help maintain corneal collagen integrity, though evidence is limited. Some patients report benefit from collagen‑supplement formulations, but these are adjuncts, not replacements for clinical care. Orthokeratology lenses or rigid gas‑permeable contact lenses can temporarily reshape the cornea and reduce visual distortion. Regular monitoring by an ophthalmologist is essential to detect progression and determine when surgical intervention (e.g., corneal cross‑linking, grafting) becomes necessary. |
| **Response Readability** | FRES: 7.58; Category: Higher |
| **Factual Accuracy (LLM-as-a-Judge)** | 1 (Accurate) |

The same original user question was evaluated across Claude 4.5, Gemini 3, GPT 5.2, and OSS-20b within each readability level using the External Calculator–Guided Prompt (Textstat) strategy. Question and response readability were assessed using the Flesch Reading Ease Score (FRES) and categorized as Basic (FRES >69), Intermediate (FRES 30–69), or Higher (FRES <30). Within each readability level, the example was selected because the responses generated by all 4 models remained in the same readability category as the original question. Selection was based solely on category-level readability alignment. Factual accuracy is also reported as 1 (accurate) or 0 (inaccurate) according to the LLM-as-a-judge evaluation, whereas completeness and empathy were evaluated separately and were not used as selection criteria. User questions are presented in paraphrased form to reduce traceability while preserving their clinical meaning. Original Question Readability and all analyses refer to the original questions. Model-generated responses are presented without grammatical correction or post hoc clinical editing. The Higher category denotes greater linguistic complexity and lower readability.
