# Supplementary material for: Prompting Strategies for Readability Alignment Between User-Generated Ophthalmology Questions and Large Language Model Answers: Comparative Evaluation Study
Source: JMIR Form Res. 2026 Nov 5;10:e97408. doi: 10.2196/97408 (PMC13739205; doi:10.2196/97408)
Supplement: Supplementary file 7 [file formative-v10-e97408-s007.docx]

**Accuracy analyses: pairwise comparisons across model-strategy configurations and associations of readability and question length with accuracy.**

**Table S1. Pairwise comparisons of binary accuracy with Holm adjustment.**

| **Condition A** | **Condition B** | **Accuracy A, % (95% CI)** | **Accuracy B, % (95% CI)** | **Difference, pp (95% CI)** | **Discordant pairs, n** | ***P*raw** | ***P*Holm** | **Holm sig.** |
| --- | --- | --- | --- | --- | --- | --- | --- | --- |
| GPT 5.2 (Baseline) | OSS-20b (FRES Prompt) | 100 [99.05, 100] | 83.75 [79.82, 87.04] | +16.25 [+12.8, +20.0] | 65; 0 | <.001 | <.001 | Yes |
| GPT 5.2 (Semantic) | OSS-20b (FRES Prompt) | 99.75 [98.6, 99.96] | 83.75 [79.82, 87.04] | +16 [+12.5, +19.8] | 65; 1 | <.001 | <.001 | Yes |
| GPT 5.2 (Textstat) | OSS-20b (FRES Prompt) | 99.75 [98.6, 99.96] | 83.75 [79.82, 87.04] | +16 [+12.5, +19.8] | 65; 1 | <.001 | <.001 | Yes |
| GPT 5.2 (Baseline) | OSS-20b (Textstat) | 100 [99.05, 100] | 85.5 [81.71, 88.61] | +14.5 [+11.2, +18.0] | 58; 0 | <.001 | <.001 | Yes |
| GPT 5.2 (FRES Prompt) | OSS-20b (FRES Prompt) | 99.5 [98.2, 99.86] | 83.75 [79.82, 87.04] | +15.75 [+12.2, +19.5] | 65; 2 | <.001 | <.001 | Yes |
| GPT 5.2 (Baseline) | OSS-20b (Semantic) | 100 [99.05, 100] | 86.25 [82.53, 89.28] | +13.75 [+10.5, +17.2] | 55; 0 | <.001 | <.001 | Yes |
| GPT 5.2 (Semantic) | OSS-20b (Textstat) | 99.75 [98.6, 99.96] | 85.5 [81.71, 88.61] | +14.25 [+10.8, +17.8] | 58; 1 | <.001 | <.001 | Yes |
| GPT 5.2 (Textstat) | OSS-20b (Textstat) | 99.75 [98.6, 99.96] | 85.5 [81.71, 88.61] | +14.25 [+10.8, +17.8] | 58; 1 | <.001 | <.001 | Yes |
| GPT 5.2 (FRES Prompt) | OSS-20b (Textstat) | 99.5 [98.2, 99.86] | 85.5 [81.71, 88.61] | +14 [+10.5, +17.5] | 57; 1 | <.001 | <.001 | Yes |
| Gemini 3 (Semantic) | OSS-20b (FRES Prompt) | 99 [97.46, 99.61] | 83.75 [79.82, 87.04] | +15.25 [+11.5, +19.0] | 64; 3 | <.001 | <.001 | Yes |
| Gemini 3 (Baseline) | OSS-20b (FRES Prompt) | 98.75 [97.11, 99.46] | 83.75 [79.82, 87.04] | +15 [+11.5, +18.8] | 63; 3 | <.001 | <.001 | Yes |
| GPT 5.2 (Semantic) | OSS-20b (Semantic) | 99.75 [98.6, 99.96] | 86.25 [82.53, 89.28] | +13.5 [+10.2, +17.0] | 55; 1 | <.001 | <.001 | Yes |
| GPT 5.2 (Textstat) | OSS-20b (Semantic) | 99.75 [98.6, 99.96] | 86.25 [82.53, 89.28] | +13.5 [+10.2, +17.0] | 55; 1 | <.001 | <.001 | Yes |
| Gemini 3 (FRES Prompt) | OSS-20b (FRES Prompt) | 98.5 [96.77, 99.31] | 83.75 [79.82, 87.04] | +14.75 [+11.2, +18.5] | 62; 3 | <.001 | <.001 | Yes |
| GPT 5.2 (FRES Prompt) | OSS-20b (Semantic) | 99.5 [98.2, 99.86] | 86.25 [82.53, 89.28] | +13.25 [+10.0, +16.8] | 54; 1 | <.001 | <.001 | Yes |
| Gemini 3 (Textstat) | OSS-20b (FRES Prompt) | 98.25 [96.43, 99.15] | 83.75 [79.82, 87.04] | +14.5 [+11.0, +18.2] | 62; 4 | <.001 | <.001 | Yes |
| Gemini 3 (Textstat) | OSS-20b (Textstat) | 98.25 [96.43, 99.15] | 85.5 [81.71, 88.61] | +12.75 [+9.5, +16.2] | 53; 2 | <.001 | <.001 | Yes |
| Gemini 3 (Baseline) | OSS-20b (Textstat) | 98.75 [97.11, 99.46] | 85.5 [81.71, 88.61] | +13.25 [+9.8, +16.8] | 56; 3 | <.001 | <.001 | Yes |
| Gemini 3 (Semantic) | OSS-20b (Textstat) | 99 [97.46, 99.61] | 85.5 [81.71, 88.61] | +13.5 [+10.0, +17.2] | 58; 4 | <.001 | <.001 | Yes |
| Gemini 3 (Semantic) | OSS-20b (Semantic) | 99 [97.46, 99.61] | 86.25 [82.53, 89.28] | +12.75 [+9.2, +16.2] | 54; 3 | <.001 | <.001 | Yes |
| Claude 4.5 (FRES Prompt) | OSS-20b (FRES Prompt) | 97 [94.83, 98.28] | 83.75 [79.82, 87.04] | +13.25 [+9.5, +17.0] | 58; 5 | <.001 | <.001 | Yes |
| GPT 5.2 (Baseline) | OSS-20b (Baseline) | 100 [99.05, 100] | 90 [86.67, 92.57] | +10 [+7.2, +13.0] | 40; 0 | <.001 | <.001 | Yes |
| Claude 4.5 (Semantic) | OSS-20b (FRES Prompt) | 97.25 [95.14, 98.46] | 83.75 [79.82, 87.04] | +13.5 [+9.8, +17.2] | 60; 6 | <.001 | <.001 | Yes |
| Gemini 3 (FRES Prompt) | OSS-20b (Textstat) | 98.5 [96.77, 99.31] | 85.5 [81.71, 88.61] | +13 [+9.5, +16.8] | 57; 5 | <.001 | <.001 | Yes |
| Gemini 3 (Baseline) | OSS-20b (Semantic) | 98.75 [97.11, 99.46] | 86.25 [82.53, 89.28] | +12.5 [+9.0, +16.0] | 54; 4 | <.001 | <.001 | Yes |
| Claude 4.5 (Baseline) | OSS-20b (FRES Prompt) | 97 [94.83, 98.28] | 83.75 [79.82, 87.04] | +13.25 [+9.5, +17.0] | 59; 6 | <.001 | <.001 | Yes |
| Gemini 3 (FRES Prompt) | OSS-20b (Semantic) | 98.5 [96.77, 99.31] | 86.25 [82.53, 89.28] | +12.25 [+8.8, +16.0] | 54; 5 | <.001 | <.001 | Yes |
| Gemini 3 (Textstat) | OSS-20b (Semantic) | 98.25 [96.43, 99.15] | 86.25 [82.53, 89.28] | +12 [+8.5, +15.5] | 53; 5 | <.001 | <.001 | Yes |
| GPT 5.2 (Semantic) | OSS-20b (Baseline) | 99.75 [98.6, 99.96] | 90 [86.67, 92.57] | +9.75 [+6.8, +12.8] | 40; 1 | <.001 | <.001 | Yes |
| GPT 5.2 (Textstat) | OSS-20b (Baseline) | 99.75 [98.6, 99.96] | 90 [86.67, 92.57] | +9.75 [+6.8, +12.8] | 40; 1 | <.001 | <.001 | Yes |
| Claude 4.5 (FRES Prompt) | OSS-20b (Textstat) | 97 [94.83, 98.28] | 85.5 [81.71, 88.61] | +11.5 [+8.0, +15.0] | 51; 5 | <.001 | <.001 | Yes |
| Claude 4.5 (Semantic) | OSS-20b (Textstat) | 97.25 [95.14, 98.46] | 85.5 [81.71, 88.61] | +11.75 [+8.2, +15.2] | 53; 6 | <.001 | <.001 | Yes |
| GPT 5.2 (FRES Prompt) | OSS-20b (Baseline) | 99.5 [98.2, 99.86] | 90 [86.67, 92.57] | +9.5 [+6.5, +12.5] | 40; 2 | <.001 | <.001 | Yes |
| Claude 4.5 (Baseline) | OSS-20b (Textstat) | 97 [94.83, 98.28] | 85.5 [81.71, 88.61] | +11.5 [+8.0, +15.2] | 53; 7 | <.001 | <.001 | Yes |
| Claude 4.5 (Semantic) | OSS-20b (Semantic) | 97.25 [95.14, 98.46] | 86.25 [82.53, 89.28] | +11 [+7.5, +14.5] | 50; 6 | <.001 | <.001 | Yes |
| Claude 4.5 (Textstat) | OSS-20b (FRES Prompt) | 95.75 [93.3, 97.33] | 83.75 [79.82, 87.04] | +12 [+8.2, +16.0] | 58; 10 | <.001 | <.001 | Yes |
| Claude 4.5 (FRES Prompt) | OSS-20b (Semantic) | 97 [94.83, 98.28] | 86.25 [82.53, 89.28] | +10.75 [+7.2, +14.2] | 50; 7 | <.001 | <.001 | Yes |
| Gemini 3 (Semantic) | OSS-20b (Baseline) | 99 [97.46, 99.61] | 90 [86.67, 92.57] | +9 [+6.0, +12.2] | 40; 4 | <.001 | <.001 | Yes |
| Claude 4.5 (Baseline) | OSS-20b (Semantic) | 97 [94.83, 98.28] | 86.25 [82.53, 89.28] | +10.75 [+7.2, +14.5] | 52; 9 | <.001 | <.001 | Yes |
| Claude 4.5 (Textstat) | OSS-20b (Textstat) | 95.75 [93.3, 97.33] | 85.5 [81.71, 88.61] | +10.25 [+6.8, +13.8] | 49; 8 | <.001 | <.001 | Yes |
| Gemini 3 (Baseline) | OSS-20b (Baseline) | 98.75 [97.11, 99.46] | 90 [86.67, 92.57] | +8.75 [+5.8, +12.0] | 39; 4 | <.001 | <.001 | Yes |
| Gemini 3 (Textstat) | OSS-20b (Baseline) | 98.25 [96.43, 99.15] | 90 [86.67, 92.57] | +8.25 [+5.2, +11.2] | 37; 4 | <.001 | <.001 | Yes |
| Gemini 3 (FRES Prompt) | OSS-20b (Baseline) | 98.5 [96.77, 99.31] | 90 [86.67, 92.57] | +8.5 [+5.5, +11.8] | 39; 5 | <.001 | <.001 | Yes |
| Claude 4.5 (Textstat) | OSS-20b (Semantic) | 95.75 [93.3, 97.33] | 86.25 [82.53, 89.28] | +9.5 [+5.8, +13.2] | 50; 12 | <.001 | <.001 | Yes |
| Claude 4.5 (Baseline) | OSS-20b (Baseline) | 97 [94.83, 98.28] | 90 [86.67, 92.57] | +7 [+4.0, +10.2] | 35; 7 | <.001 | .001 | Yes |
| Claude 4.5 (FRES Prompt) | OSS-20b (Baseline) | 97 [94.83, 98.28] | 90 [86.67, 92.57] | +7 [+4.0, +10.2] | 35; 7 | <.001 | .001 | Yes |
| Claude 4.5 (Semantic) | OSS-20b (Baseline) | 97.25 [95.14, 98.46] | 90 [86.67, 92.57] | +7.25 [+4.0, +10.5] | 37; 8 | <.001 | .001 | Yes |
| Claude 4.5 (Textstat) | GPT 5.2 (Baseline) | 95.75 [93.3, 97.33] | 100 [99.05, 100] | -4.25 [-6.2, -2.5] | 0; 17 | <.001 | .001 | Yes |
| Claude 4.5 (Textstat) | GPT 5.2 (Textstat) | 95.75 [93.3, 97.33] | 99.75 [98.6, 99.96] | -4 [-6.0, -2.2] | 0; 16 | <.001 | .002 | Yes |
| Claude 4.5 (Textstat) | GPT 5.2 (Semantic) | 95.75 [93.3, 97.33] | 99.75 [98.6, 99.96] | -4 [-6.2, -2.0] | 1; 17 | <.001 | .01 | Yes |
| OSS-20b (FRES Prompt) | OSS-20b (Baseline) | 83.75 [79.82, 87.04] | 90 [86.67, 92.57] | -6.25 [-9.5, -3.0] | 11; 36 | <.001 | .02 | Yes |
| Claude 4.5 (Baseline) | GPT 5.2 (Baseline) | 97 [94.83, 98.28] | 100 [99.05, 100] | -3 [-4.8, -1.5] | 0; 12 | <.001 | .03 | Yes |
| Claude 4.5 (FRES Prompt) | GPT 5.2 (Baseline) | 97 [94.83, 98.28] | 100 [99.05, 100] | -3 [-4.8, -1.5] | 0; 12 | <.001 | .03 | Yes |
| Claude 4.5 (Textstat) | GPT 5.2 (FRES Prompt) | 95.75 [93.3, 97.33] | 99.5 [98.2, 99.86] | -3.75 [-6.0, -1.8] | 2; 17 | <.001 | .049 | Yes |
| Claude 4.5 (Textstat) | OSS-20b (Baseline) | 95.75 [93.3, 97.33] | 90 [86.67, 92.57] | +5.75 [+2.5, +9.0] | 34; 11 | <.001 | .054 | No |
| Claude 4.5 (FRES Prompt) | GPT 5.2 (Textstat) | 97 [94.83, 98.28] | 99.75 [98.6, 99.96] | -2.75 [-4.5, -1.2] | 0; 11 | <.001 | .06 | No |
| Claude 4.5 (Semantic) | GPT 5.2 (Baseline) | 97.25 [95.14, 98.46] | 100 [99.05, 100] | -2.75 [-4.5, -1.2] | 0; 11 | <.001 | .06 | No |
| Claude 4.5 (Baseline) | GPT 5.2 (Semantic) | 97 [94.83, 98.28] | 99.75 [98.6, 99.96] | -2.75 [-4.5, -1.0] | 1; 12 | .003 | .22 | No |
| Claude 4.5 (Baseline) | GPT 5.2 (Textstat) | 97 [94.83, 98.28] | 99.75 [98.6, 99.96] | -2.75 [-4.5, -1.0] | 1; 12 | .003 | .22 | No |
| Claude 4.5 (FRES Prompt) | GPT 5.2 (Semantic) | 97 [94.83, 98.28] | 99.75 [98.6, 99.96] | -2.75 [-4.5, -1.0] | 1; 12 | .003 | .22 | No |
| Claude 4.5 (Textstat) | Gemini 3 (Semantic) | 95.75 [93.3, 97.33] | 99 [97.46, 99.61] | -3.25 [-5.5, -1.2] | 3; 16 | .004 | .27 | No |
| Claude 4.5 (Semantic) | GPT 5.2 (Semantic) | 97.25 [95.14, 98.46] | 99.75 [98.6, 99.96] | -2.5 [-4.2, -1.0] | 1; 11 | .006 | .38 | No |
| Claude 4.5 (Semantic) | GPT 5.2 (Textstat) | 97.25 [95.14, 98.46] | 99.75 [98.6, 99.96] | -2.5 [-4.2, -1.0] | 1; 11 | .006 | .38 | No |
| Claude 4.5 (Baseline) | GPT 5.2 (FRES Prompt) | 97 [94.83, 98.28] | 99.5 [98.2, 99.86] | -2.5 [-4.5, -0.8] | 2; 12 | .01 | .74 | No |
| Claude 4.5 (FRES Prompt) | GPT 5.2 (FRES Prompt) | 97 [94.83, 98.28] | 99.5 [98.2, 99.86] | -2.5 [-4.5, -0.8] | 2; 12 | .01 | .74 | No |
| Gemini 3 (Textstat) | GPT 5.2 (Baseline) | 98.25 [96.43, 99.15] | 100 [99.05, 100] | -1.75 [-3.2, -0.5] | 0; 7 | .02 | .84 | No |
| OSS-20b (Textstat) | OSS-20b (Baseline) | 85.5 [81.71, 88.61] | 90 [86.67, 92.57] | -4.5 [-8.0, -1.0] | 16; 34 | .02 | .84 | No |
| Claude 4.5 (Textstat) | Gemini 3 (Baseline) | 95.75 [93.3, 97.33] | 98.75 [97.11, 99.46] | -3 [-5.2, -0.8] | 5; 17 | .02 | .90 | No |
| Claude 4.5 (Baseline) | Gemini 3 (FRES Prompt) | 97 [94.83, 98.28] | 98.5 [96.77, 99.31] | -1.5 [-3.5, +0.5] | 5; 11 | .21 | >.99 | No |
| Claude 4.5 (Baseline) | Gemini 3 (Semantic) | 97 [94.83, 98.28] | 99 [97.46, 99.61] | -2 [-3.8, -0.2] | 3; 11 | .06 | >.99 | No |
| Claude 4.5 (Baseline) | Gemini 3 (Textstat) | 97 [94.83, 98.28] | 98.25 [96.43, 99.15] | -1.25 [-3.5, +0.8] | 7; 12 | .36 | >.99 | No |
| Claude 4.5 (Baseline) | Gemini 3 (Baseline) | 97 [94.83, 98.28] | 98.75 [97.11, 99.46] | -1.75 [-3.8, +0.0] | 4; 11 | .12 | >.99 | No |
| Claude 4.5 (FRES Prompt) | Claude 4.5 (Semantic) | 97 [94.83, 98.28] | 97.25 [95.14, 98.46] | -0.25 [-2.0, +1.5] | 6; 7 | >.99 | >.99 | No |
| Claude 4.5 (FRES Prompt) | Claude 4.5 (Textstat) | 97 [94.83, 98.28] | 95.75 [93.3, 97.33] | +1.25 [-0.2, +3.0] | 8; 3 | .23 | >.99 | No |
| Claude 4.5 (FRES Prompt) | Claude 4.5 (Baseline) | 97 [94.83, 98.28] | 97 [94.83, 98.28] | 0 [-1.8, +1.8] | 6; 6 | >.99 | >.99 | No |
| Claude 4.5 (FRES Prompt) | Gemini 3 (FRES Prompt) | 97 [94.83, 98.28] | 98.5 [96.77, 99.31] | -1.5 [-3.5, +0.5] | 5; 11 | .21 | >.99 | No |
| Claude 4.5 (FRES Prompt) | Gemini 3 (Semantic) | 97 [94.83, 98.28] | 99 [97.46, 99.61] | -2 [-3.8, -0.2] | 3; 11 | .06 | >.99 | No |
| Claude 4.5 (FRES Prompt) | Gemini 3 (Textstat) | 97 [94.83, 98.28] | 98.25 [96.43, 99.15] | -1.25 [-3.2, +0.8] | 6; 11 | .33 | >.99 | No |
| Claude 4.5 (FRES Prompt) | Gemini 3 (Baseline) | 97 [94.83, 98.28] | 98.75 [97.11, 99.46] | -1.75 [-3.5, +0.0] | 3; 10 | .09 | >.99 | No |
| Claude 4.5 (Semantic) | Claude 4.5 (Textstat) | 97.25 [95.14, 98.46] | 95.75 [93.3, 97.33] | +1.5 [-0.5, +3.5] | 11; 5 | .21 | >.99 | No |
| Claude 4.5 (Semantic) | Claude 4.5 (Baseline) | 97.25 [95.14, 98.46] | 97 [94.83, 98.28] | +0.25 [-1.8, +2.2] | 8; 7 | >.99 | >.99 | No |
| Claude 4.5 (Semantic) | Gemini 3 (FRES Prompt) | 97.25 [95.14, 98.46] | 98.5 [96.77, 99.31] | -1.25 [-3.2, +0.5] | 5; 10 | .30 | >.99 | No |
| Claude 4.5 (Semantic) | Gemini 3 (Semantic) | 97.25 [95.14, 98.46] | 99 [97.46, 99.61] | -1.75 [-3.5, +0.0] | 3; 10 | .09 | >.99 | No |
| Claude 4.5 (Semantic) | Gemini 3 (Textstat) | 97.25 [95.14, 98.46] | 98.25 [96.43, 99.15] | -1 [-3.0, +1.0] | 7; 11 | .48 | >.99 | No |
| Claude 4.5 (Semantic) | Gemini 3 (Baseline) | 97.25 [95.14, 98.46] | 98.75 [97.11, 99.46] | -1.5 [-3.5, +0.5] | 5; 11 | .21 | >.99 | No |
| Claude 4.5 (Semantic) | GPT 5.2 (FRES Prompt) | 97.25 [95.14, 98.46] | 99.5 [98.2, 99.86] | -2.25 [-4.0, -0.5] | 2; 11 | .02 | >.99 | No |
| Claude 4.5 (Textstat) | Claude 4.5 (Baseline) | 95.75 [93.3, 97.33] | 97 [94.83, 98.28] | -1.25 [-3.2, +0.8] | 6; 11 | .33 | >.99 | No |
| Claude 4.5 (Textstat) | Gemini 3 (FRES Prompt) | 95.75 [93.3, 97.33] | 98.5 [96.77, 99.31] | -2.75 [-5.0, -0.5] | 5; 16 | .08 | >.99 | No |
| Claude 4.5 (Textstat) | Gemini 3 (Textstat) | 95.75 [93.3, 97.33] | 98.25 [96.43, 99.15] | -2.5 [-5.0, -0.2] | 7; 17 | .06 | >.99 | No |
| GPT 5.2 (FRES Prompt) | GPT 5.2 (Semantic) | 99.5 [98.2, 99.86] | 99.75 [98.6, 99.96] | -0.25 [-1.2, +0.5] | 1; 2 | >.99 | >.99 | No |
| GPT 5.2 (FRES Prompt) | GPT 5.2 (Textstat) | 99.5 [98.2, 99.86] | 99.75 [98.6, 99.96] | -0.25 [-1.0, +0.5] | 1; 2 | >.99 | >.99 | No |
| GPT 5.2 (FRES Prompt) | GPT 5.2 (Baseline) | 99.5 [98.2, 99.86] | 100 [99.05, 100] | -0.5 [-1.2, +0.0] | 0; 2 | .50 | >.99 | No |
| GPT 5.2 (Semantic) | GPT 5.2 (Textstat) | 99.75 [98.6, 99.96] | 99.75 [98.6, 99.96] | 0 [-0.8, +0.8] | 1; 1 | >.99 | >.99 | No |
| GPT 5.2 (Semantic) | GPT 5.2 (Baseline) | 99.75 [98.6, 99.96] | 100 [99.05, 100] | -0.25 [-0.8, +0.0] | 0; 1 | >.99 | >.99 | No |
| GPT 5.2 (Textstat) | GPT 5.2 (Baseline) | 99.75 [98.6, 99.96] | 100 [99.05, 100] | -0.25 [-0.8, +0.0] | 0; 1 | >.99 | >.99 | No |
| Gemini 3 (Baseline) | GPT 5.2 (FRES Prompt) | 98.75 [97.11, 99.46] | 99.5 [98.2, 99.86] | -0.75 [-2.0, +0.5] | 2; 5 | .45 | >.99 | No |
| Gemini 3 (Baseline) | GPT 5.2 (Semantic) | 98.75 [97.11, 99.46] | 99.75 [98.6, 99.96] | -1 [-2.2, +0.0] | 1; 5 | .22 | >.99 | No |
| Gemini 3 (Baseline) | GPT 5.2 (Textstat) | 98.75 [97.11, 99.46] | 99.75 [98.6, 99.96] | -1 [-2.2, +0.0] | 1; 5 | .22 | >.99 | No |
| Gemini 3 (Baseline) | GPT 5.2 (Baseline) | 98.75 [97.11, 99.46] | 100 [99.05, 100] | -1.25 [-2.5, -0.2] | 0; 5 | .06 | >.99 | No |
| Gemini 3 (FRES Prompt) | Gemini 3 (Semantic) | 98.5 [96.77, 99.31] | 99 [97.46, 99.61] | -0.5 [-2.0, +0.8] | 3; 5 | .73 | >.99 | No |
| Gemini 3 (FRES Prompt) | Gemini 3 (Textstat) | 98.5 [96.77, 99.31] | 98.25 [96.43, 99.15] | +0.25 [-1.2, +1.8] | 6; 5 | >.99 | >.99 | No |
| Gemini 3 (FRES Prompt) | Gemini 3 (Baseline) | 98.5 [96.77, 99.31] | 98.75 [97.11, 99.46] | -0.25 [-1.8, +1.2] | 4; 5 | >.99 | >.99 | No |
| Gemini 3 (FRES Prompt) | GPT 5.2 (FRES Prompt) | 98.5 [96.77, 99.31] | 99.5 [98.2, 99.86] | -1 [-2.5, +0.2] | 2; 6 | .29 | >.99 | No |
| Gemini 3 (FRES Prompt) | GPT 5.2 (Semantic) | 98.5 [96.77, 99.31] | 99.75 [98.6, 99.96] | -1.25 [-2.5, +0.0] | 1; 6 | .13 | >.99 | No |
| Gemini 3 (FRES Prompt) | GPT 5.2 (Textstat) | 98.5 [96.77, 99.31] | 99.75 [98.6, 99.96] | -1.25 [-2.5, +0.0] | 1; 6 | .13 | >.99 | No |
| Gemini 3 (FRES Prompt) | GPT 5.2 (Baseline) | 98.5 [96.77, 99.31] | 100 [99.05, 100] | -1.5 [-2.8, -0.5] | 0; 6 | .03 | >.99 | No |
| Gemini 3 (Semantic) | Gemini 3 (Textstat) | 99 [97.46, 99.61] | 98.25 [96.43, 99.15] | +0.75 [-0.8, +2.5] | 7; 4 | .55 | >.99 | No |
| Gemini 3 (Semantic) | Gemini 3 (Baseline) | 99 [97.46, 99.61] | 98.75 [97.11, 99.46] | +0.25 [-1.2, +1.8] | 5; 4 | >.99 | >.99 | No |
| Gemini 3 (Semantic) | GPT 5.2 (FRES Prompt) | 99 [97.46, 99.61] | 99.5 [98.2, 99.86] | -0.5 [-1.8, +0.8] | 2; 4 | .69 | >.99 | No |
| Gemini 3 (Semantic) | GPT 5.2 (Semantic) | 99 [97.46, 99.61] | 99.75 [98.6, 99.96] | -0.75 [-2.0, +0.2] | 1; 4 | .38 | >.99 | No |
| Gemini 3 (Semantic) | GPT 5.2 (Textstat) | 99 [97.46, 99.61] | 99.75 [98.6, 99.96] | -0.75 [-2.0, +0.2] | 1; 4 | .38 | >.99 | No |
| Gemini 3 (Semantic) | GPT 5.2 (Baseline) | 99 [97.46, 99.61] | 100 [99.05, 100] | -1 [-2.0, -0.2] | 0; 4 | .13 | >.99 | No |
| Gemini 3 (Textstat) | Gemini 3 (Baseline) | 98.25 [96.43, 99.15] | 98.75 [97.11, 99.46] | -0.5 [-2.0, +0.8] | 3; 5 | .73 | >.99 | No |
| Gemini 3 (Textstat) | GPT 5.2 (FRES Prompt) | 98.25 [96.43, 99.15] | 99.5 [98.2, 99.86] | -1.25 [-2.8, +0.2] | 2; 7 | .18 | >.99 | No |
| Gemini 3 (Textstat) | GPT 5.2 (Semantic) | 98.25 [96.43, 99.15] | 99.75 [98.6, 99.96] | -1.5 [-3.0, -0.2] | 1; 7 | .07 | >.99 | No |
| Gemini 3 (Textstat) | GPT 5.2 (Textstat) | 98.25 [96.43, 99.15] | 99.75 [98.6, 99.96] | -1.5 [-3.0, -0.2] | 1; 7 | .07 | >.99 | No |
| OSS-20b (FRES Prompt) | OSS-20b (Semantic) | 83.75 [79.82, 87.04] | 86.25 [82.53, 89.28] | -2.5 [-6.5, +1.5] | 28; 38 | .27 | >.99 | No |
| OSS-20b (FRES Prompt) | OSS-20b (Textstat) | 83.75 [79.82, 87.04] | 85.5 [81.71, 88.61] | -1.75 [-5.2, +1.8] | 24; 31 | .42 | >.99 | No |
| OSS-20b (Semantic) | OSS-20b (Textstat) | 86.25 [82.53, 89.28] | 85.5 [81.71, 88.61] | +0.75 [-3.2, +4.8] | 34; 31 | .80 | >.99 | No |
| OSS-20b (Semantic) | OSS-20b (Baseline) | 86.25 [82.53, 89.28] | 90 [86.67, 92.57] | -3.75 [-7.2, -0.5] | 17; 32 | .04 | >.99 | No |

Accuracy is the percentage of correct ratings (1=correct; 0=incorrect). Accuracy 95% CIs are Wilson intervals, and paired accuracy-difference 95% CIs are percentile bootstrap intervals based on 50,000 resamples. Discordant pairs are reported as A correct/B incorrect; A incorrect/B correct. Pairwise comparisons used 2-sided exact McNemar tests, with P values adjusted across all 120 comparisons using the Holm procedure. P values are reported to 2 decimal places, except when P<.01 or when rounding would alter the interpretation of statistical significance. Values below .001 are reported as P<.001, and values above .99 as P>.99. Statistical significance was defined as Holm-adjusted P<.05. Praw indicates the unadjusted P value; PHolm, the Holm-adjusted P value; and Holm sig., statistical significance after Holm correction. Abbreviations: CI, confidence interval; pp, percentage points.

**Table S2. Question readability (FRES) and accuracy.**

| **Model** | **Strategy** | **Accuracy, % (n/N)** | **OR per 10 FRES points**  **(95% CI)** | **Predicted difference P75−P25, pp** | ***P*raw** | ***P*Holm** | **Holm sig.** |
| --- | --- | --- | --- | --- | --- | --- | --- |
| GPT 5.2 | Baseline | 100.00 (400/400) | Not estimableᵃ | — | — | — | — |
|  | Semantic | 99.75 (399/400) | 0.04  [<0.01, 3.48] | −0.02ᵇ | .16 | >.99 | No |
|  | FRES Prompt | 99.50 (398/400) | 1.31  [0.79, 2.14] | +0.52ᵇ | .29 | >.99 | No |
|  | Textstat | 99.75 (399/400) | 0.63  [0.16, 2.42] | −0.34ᵇ | .50 | >.99 | No |
| Gemini 3 | Baseline | 98.75 (395/400) | 1.22  [0.90, 1.65] | +1.05 | .20 | >.99 | No |
|  | Semantic | 99.00 (396/400) | 1.02  [0.71, 1.45] | +0.08 | .92 | >.99 | No |
|  | FRES Prompt | 98.50 (394/400) | 0.95  [0.70, 1.29] | −0.34 | .75 | >.99 | No |
|  | Textstat | 98.25 (393/400) | 0.67  [0.42, 1.07] | −2.26 | .09 | .83 | No |
| Claude 4.5 | Baseline | 97.00 (388/400) | 1.07  [0.87, 1.30] | +0.88 | .53 | >.99 | No |
|  | Semantic | 97.25 (389/400) | 0.71  [0.50, 1.00] | −3.28 | .049 | .54 | No |
|  | FRES Prompt | 97.00 (388/400) | 0.77  [0.58, 1.03] | −2.96 | .08 | .80 | No |
|  | Textstat | 95.75 (383/400) | 0.62  [0.44, 0.87] | −5.90 | .005 | .06 | No |
| OSS-20b | Baseline | 90.00 (360/400) | 0.75  [0.64, 0.89] | −10.19 | <.001 | .01 | Yes |
|  | Semantic | 86.25 (345/400) | 0.96  [0.87, 1.07] | −2.12 | .48 | >.99 | No |
|  | FRES Prompt | 83.75 (335/400) | 0.74  [0.65, 0.85] | −16.45 | <.001 | <.001 | Yes |
|  | Textstat | 85.50 (342/400) | 0.74  [0.64, 0.85] | −14.83 | <.001 | <.001 | Yes |

Each row is a separate univariable logistic regression of binary accuracy (1=correct; 0=incorrect) on the Flesch Reading Ease Score (FRES) of the original user question (n=400 items per model-strategy configuration). Higher FRES indicates easier text; therefore, an OR<1 indicates lower accuracy for easier questions. ORs and Wald 95% CIs are expressed per 10-point increase in FRES. The predicted difference is the model-based accuracy at the 75th percentile of question FRES (75.26) minus that at the 25th percentile (28.37). *P* values are 2-sided Wald tests, adjusted across the 15 estimable configurations using the Holm procedure. ᵃNot estimable because all 400 responses were correct (no outcome variance). ᵇ2 or fewer incorrect responses; estimates are unstable and should be interpreted with caution. *P*raw indicates the unadjusted *P* value; *P*Holm, the Holm-adjusted *P* value; and Holm sig., statistical significance after Holm correction (*P*Holm<.05). Abbreviations: FRES, Flesch Reading Ease Score; OR, odds ratio; pp, percentage points.

**Table S3. Response readability (FRES) and accuracy.**

| **Model** | **Strategy** | **Accuracy, % (n/N)** | **Response FRES, mean (SD)** | **OR per 10 FRES points**  **(95% CI)** | **Predicted difference P75−P25, pp** | ***P*raw** | ***P*Holm** | **Holm sig.** |
| --- | --- | --- | --- | --- | --- | --- | --- | --- |
| GPT 5.2 | Baseline | 100.00 (400/400) | 36.43 (11.47) | Not estimableᵃ | — | — | — | — |
|  | Semantic | 99.75 (399/400) | 39.15 (15.64) | 0.77  [0.22, 2.62] | −0.12ᵇ | .67 | >.99 | No |
|  | FRES Prompt | 99.50 (398/400) | 54.03 (13.34) | 0.62  [0.21, 1.86] | −0.40ᵇ | .39 | >.99 | No |
|  | Textstat | 99.75 (399/400) | 52.63 (23.59) | 0.15  [0.01, 2.69] | −0.16ᵇ | .20 | >.99 | No |
| Gemini 3 | Baseline | 98.75 (395/400) | 37.18 (8.69) | 1.51  [0.54, 4.20] | +0.58 | .43 | >.99 | No |
|  | Semantic | 99.00 (396/400) | 42.34 (22.61) | 0.86  [0.55, 1.34] | −0.44 | .51 | >.99 | No |
|  | FRES Prompt | 98.50 (394/400) | 50.17 (25.80) | 0.86  [0.61, 1.23] | −0.81 | .42 | >.99 | No |
|  | Textstat | 98.25 (393/400) | 48.35 (26.71) | 0.69  [0.46, 1.03] | −2.63 | .07 | .65 | No |
| Claude 4.5 | Baseline | 97.00 (388/400) | 31.21 (11.35) | 1.61  [0.98, 2.63] | +1.86 | .06 | .58 | No |
|  | Semantic | 97.25 (389/400) | 30.09 (25.27) | 0.71  [0.56, 0.91] | −2.46 | .007 | .08 | No |
|  | FRES Prompt | 97.00 (388/400) | 44.50 (22.64) | 0.76  [0.56, 1.03] | −2.57 | .08 | .65 | No |
|  | Textstat | 95.75 (383/400) | 43.41 (21.33) | 0.55  [0.39, 0.77] | −5.97 | <.001 | .008 | Yes |
| OSS-20b | Baseline | 90.00 (360/400) | 38.33 (10.99) | 0.69  [0.51, 0.95] | −4.48 | .02 | .23 | No |
|  | Semantic | 86.25 (345/400) | 46.92 (13.90) | 1.03  [0.84, 1.27] | +0.68 | .75 | >.99 | No |
|  | FRES Prompt | 83.75 (335/400) | 42.57 (21.47) | 0.77  [0.67, 0.89] | −10.34 | <.001 | .004 | Yes |
|  | Textstat | 85.50 (342/400) | 49.56 (23.76) | 0.72  [0.62, 0.84] | −14.60 | <.001 | <.001 | Yes |

Each row is a separate univariable logistic regression of binary accuracy on the FRES of the response generated by that model-strategy configuration (n=400 responses per configuration). An OR<1 indicates lower accuracy for easier-to-read responses. ORs and Wald 95% CIs are expressed per 10-point increase in response FRES. The predicted difference compares model-based accuracy at the 75th and 25th percentiles of each configuration's own response FRES distribution. *P* values are 2-sided Wald tests, adjusted across the 15 estimable configurations using the Holm procedure. ᵃNot estimable because all 400 responses were correct. ᵇ2 or fewer incorrect responses; estimates are unstable. *P*raw indicates the unadjusted *P* value; *P*Holm, the Holm-adjusted *P* value; and Holm sig., statistical significance after Holm correction (*P*Holm<.05). Abbreviations: FRES, Flesch Reading Ease Score; OR, odds ratio; pp, percentage points.

**Table S4. Target response readability level and accuracy.**

| **Model** | **Strategy** | **n (B/I/H)** | **Accuracy, % (B / I / H)** | **Difference H−B, pp** | **χ² (df=2)** | **Cramér V** | ***P*raw**  **χ²** | ***P*Holm**  **χ²** | **Trend z** | ***P*raw**  **trend** | ***P*Holm**  **trend** | **Holm sig.** |
| --- | --- | --- | --- | --- | --- | --- | --- | --- | --- | --- | --- | --- |
| GPT 5.2 | Semantic | 49/206/145 | 100.0 / 99.5 / 100.0 | +0.00 | 0.94 | 0.05 | .62 | >.99 | 0.37 | .71 | >.99 | No |
|  | FRES Prompt | 111/286/3 | 99.1 / 99.7 / 100.0 | +0.90 | 0.50 | 0.04 | .78 | >.99 | 0.71 | .48 | >.99 | No |
|  | Textstat | 160/127/113 | 99.4 / 100.0 / 100.0 | +0.62 | 1.50 | 0.06 | .47 | >.99 | 1.08 | .28 | >.99 | No |
| Gemini 3 | Semantic | 66/208/126 | 100.0 / 98.1 / 100.0 | +0.00 | 3.73 | 0.10 | .15 | >.99 | 0.45 | .66 | >.99 | No |
|  | FRES Prompt | 138/186/76 | 98.6 / 97.8 / 100.0 | +1.45 | 1.69 | 0.07 | .43 | >.99 | 0.62 | .54 | >.99 | No |
|  | Textstat | 160/127/113 | 96.3 / 99.2 / 100.0 | +3.75 | 6.42 | 0.13 | .04 | .32 | 2.41 | .02 | .13 | No |
| Claude 4.5 | Semantic | 76/145/179 | 93.4 / 96.6 / 99.4 | +6.02 | 7.64 | 0.14 | .02 | .20 | 2.76 | .006 | .051 | No |
|  | FRES Prompt | 163/202/35 | 95.7 / 98.0 / 97.1 | +1.44 | 1.66 | 0.06 | .44 | >.99 | 1.01 | .31 | >.99 | No |
|  | Textstat | 160/127/113 | 91.3 / 97.6 / 100.0 | +8.75 | 14.09 | 0.19 | <.001 | .009 | 3.64 | <.001 | .003 | Yes |
| OSS-20b | Semantic | 38/349/13 | 92.1 / 85.4 / 92.3 | +0.20 | 1.72 | 0.07 | .42 | >.99 | −0.59 | .55 | >.99 | No |
|  | FRES Prompt | 80/214/106 | 72.5 / 81.8 / 96.2 | +23.73 | 20.18 | 0.22 | <.001 | <.001 | 4.44 | <.001 | <.001 | Yes |
|  | Textstat | 160/127/113 | 76.9 / 88.2 / 94.7 | +17.82 | 18.04 | 0.21 | <.001 | .001 | 4.20 | <.001 | <.001 | Yes |
| All models (pooled) | All 3 strategies | 1361/2304/1135 | 92.8 / 94.4 / 98.9 | +6.06 | 51.07 | 0.10 | <.001 | <.001 | 6.80 | <.001 | <.001 | Yes |
|  | Semantic | 229/908/463 | 96.5 / 93.3 / 99.6 | +3.06 | 29.14 | 0.13 | <.001 | <.001 | 3.10 | .002 | .004 | Yes |
|  | FRES Prompt | 492/888/220 | 93.5 / 94.6 / 97.7 | +4.23 | 5.45 | 0.06 | .07 | .07 | 2.17 | .03 | .03 | Yes |
|  | Textstat | 640/508/452 | 90.9 / 96.3 / 98.7 | +7.74 | 35.40 | 0.15 | <.001 | <.001 | 5.82 | <.001 | <.001 | Yes |

The target readability level is the level that the response was instructed or intended to match: for Textstat, the tier computed externally from the question FRES (identical for all models and equal to the question's own readability tier); for FRES Prompt, the tier the model inferred from the question using the FRES definition; and for Semantic, the level the model inferred from the question wording. Baseline was not included because it has no target level. Accuracy was compared across target levels using Pearson χ² tests of independence (3×2 tables) and Cochran-Armitage tests for linear trend (Basic=1, Intermediate=2, Higher=3); a positive z indicates higher accuracy with a more complex target level. Holm adjustment was applied separately to the 12 model-strategy configurations and to the 4 pooled analyses, and within each family separately for the χ² and trend tests. Holm sig. indicates *P*Holm<.05 for either test. Sparse cells (for example, 3 Higher-level targets for GPT 5.2 FRES Prompt) limit the χ² approximation in some configurations. B/I/H indicates Basic/Intermediate/Higher; *P*raw, the unadjusted *P* value; and *P*Holm, the Holm-adjusted *P* value. Abbreviations: FRES, Flesch Reading Ease Score; pp, percentage points.

**Table S5. Question length (number of words) and accuracy.**

| **Analysis level** | **Model** | **Strategy** | **Accuracy, % (n/N)** | **OR per 10 words**  **(95% CI)** | **Predicted difference P75−P25, pp** | ***P*raw** | ***P*Holm** | **Holm sig.** |
| --- | --- | --- | --- | --- | --- | --- | --- | --- |
| Pooled | All | All | 95.38 (6104/6400) | 0.95  [0.93, 0.97] | −1.82 | <.001 | <.001 | Yes |
| By model | GPT 5.2 | All | 99.75 (1596/1600) | 1.16  [0.88, 1.54] | +0.29 | .30 | .30 | No |
|  | Gemini 3 | All | 98.62 (1578/1600) | 0.93  [0.88, 0.97] | −0.76 | .003 | .006 | Yes |
|  | Claude 4.5 | All | 96.75 (1548/1600) | 0.94  [0.91, 0.98] | −1.49 | .001 | .004 | Yes |
|  | OSS-20b | All | 86.38 (1382/1600) | 0.95  [0.93, 0.97] | −5.24 | <.001 | <.001 | Yes |
| By strategy | All | Baseline | 96.44 (1543/1600) | 0.97  [0.93, 1.00] | −1.00 | .07 | .15 | No |
|  |  | Semantic | 95.56 (1529/1600) | 0.99  [0.95, 1.03] | −0.44 | .53 | .53 | No |
|  |  | FRES Prompt | 94.69 (1515/1600) | 0.94  [0.91, 0.97] | −2.48 | <.001 | <.001 | Yes |
|  |  | Textstat | 94.81 (1517/1600) | 0.92  [0.90, 0.95] | −2.90 | <.001 | <.001 | Yes |
| By model and strategy | GPT 5.2 | Baseline | 100.00 (400/400) | Not estimableᵃ | — | — | — | — |
|  |  | Semantic | 99.75 (399/400) | 21.67  [0.01, 37,931] | +0.18ᵇ | .42 | >.99 | No |
|  |  | FRES Prompt | 99.50 (398/400) | 1.14  [0.78, 1.67] | +0.54ᵇ | .49 | >.99 | No |
|  |  | Textstat | 99.75 (399/400) | 1.04  [0.71, 1.51] | +0.08ᵇ | .85 | >.99 | No |
|  | Gemini 3 | Baseline | 98.75 (395/400) | 0.96  [0.85, 1.08] | −0.45 | .47 | >.99 | No |
|  |  | Semantic | 99.00 (396/400) | 0.99  [0.85, 1.17] | −0.06 | .93 | >.99 | No |
|  |  | FRES Prompt | 98.50 (394/400) | 0.91  [0.83, 1.00] | −0.93 | .053 | .59 | No |
|  |  | Textstat | 98.25 (393/400) | 0.89  [0.82, 0.97] | −1.19 | .01 | .12 | No |
|  | Claude 4.5 | Baseline | 97.00 (388/400) | 1.00  [0.91, 1.10] | −0.07 | .95 | >.99 | No |
|  |  | Semantic | 97.25 (389/400) | 0.95  [0.87, 1.03] | −1.10 | .21 | >.99 | No |
|  |  | FRES Prompt | 97.00 (388/400) | 0.94  [0.87, 1.01] | −1.43 | .10 | .88 | No |
|  |  | Textstat | 95.75 (383/400) | 0.91  [0.85, 0.97] | −2.74 | .002 | .03 | Yes |
|  | OSS-20b | Baseline | 90.00 (360/400) | 0.95  [0.91, 1.00] | −3.41 | .06 | .59 | No |
|  |  | Semantic | 86.25 (345/400) | 0.99  [0.95, 1.04] | −0.65 | .79 | >.99 | No |
|  |  | FRES Prompt | 83.75 (335/400) | 0.93  [0.89, 0.96] | −8.24 | <.001 | .003 | Yes |
|  |  | Textstat | 85.50 (342/400) | 0.92  [0.88, 0.96] | −7.98 | <.001 | .002 | Yes |

Each row is a separate univariable logistic regression of binary accuracy on the number of words in the original user question (mean 63.97, SD 59.73; 25th and 75th percentiles 13 and 99 words). Pooled, by-model, and by-strategy analyses stack the corresponding model-strategy responses (N=6400, 1600, and 1600 responses, respectively) and do not account for the repeated use of the same 400 questions. An OR<1 indicates lower accuracy for longer questions. ORs and Wald 95% CIs are expressed per 10-word increase. The predicted difference is the model-based accuracy at 99 words minus that at 13 words. Holm adjustment was applied within each analysis level (4 models, 4 strategies, and 15 estimable model-strategy configurations). ᵃNot estimable because all 400 responses were correct. ᵇ2 or fewer incorrect responses; estimates are unstable. *P*raw indicates the unadjusted *P* value; *P*Holm, the Holm-adjusted *P* value; and Holm sig., statistical significance after Holm correction (*P*Holm<.05). Abbreviations: OR, odds ratio; pp, percentage points.

*P* values are reported to 2 decimal places, except when *P*<.01 or when rounding would alter the interpretation of statistical significance. Values below .001 are reported as *P*<.001, and values above .99 as *P*>.99.
