# Supplementary material for: Prompting Strategies for Readability Alignment Between User-Generated Ophthalmology Questions and Large Language Model Answers: Comparative Evaluation Study
Source: JMIR Form Res. 2026 Nov 5;10:e97408. doi: 10.2196/97408 (PMC13739205; doi:10.2196/97408)
Supplement: Supplementary file 8 [file formative-v10-e97408-s008.docx]

**Pairwise comparisons of completeness scores with Holm adjustment.**

| **Condition A** | **Condition B** | **Mean A (95% CI)** | **Mean B (95% CI)** | **Mean difference (95% CI)** | **Wilcoxon W** | ***P*raw** | ***P*Holm** | **Holm sig.** |
| --- | --- | --- | --- | --- | --- | --- | --- | --- |
| Claude 4.5 (FRES Prompt) | OSS-20b (FRES Prompt) | 2.960 [2.941, 2.979] | 2.623 [2.573, 2.672] | 0.338 [0.287, 0.388] | 990.0 | <.001 | <.001 | Yes |
| Claude 4.5 (FRES Prompt) | OSS-20b (Textstat) | 2.960 [2.941, 2.979] | 2.625 [2.577, 2.673] | 0.335 [0.286, 0.384] | 1642.5 | <.001 | <.001 | Yes |
| Claude 4.5 (Semantic) | OSS-20b (FRES Prompt) | 2.958 [2.936, 2.979] | 2.623 [2.573, 2.672] | 0.335 [0.285, 0.385] | 1318.0 | <.001 | <.001 | Yes |
| Claude 4.5 (Semantic) | OSS-20b (Textstat) | 2.958 [2.936, 2.979] | 2.625 [2.577, 2.673] | 0.333 [0.282, 0.383] | 2289.0 | <.001 | <.001 | Yes |
| Claude 4.5 (Baseline) | OSS-20b (FRES Prompt) | 2.950 [2.929, 2.971] | 2.623 [2.573, 2.672] | 0.328 [0.277, 0.378] | 1650.0 | <.001 | <.001 | Yes |
| Claude 4.5 (Baseline) | OSS-20b (Textstat) | 2.950 [2.929, 2.971] | 2.625 [2.577, 2.673] | 0.325 [0.275, 0.375] | 1977.0 | <.001 | <.001 | Yes |
| GPT 5.2 (Semantic) | OSS-20b (Textstat) | 2.942 [2.920, 2.965] | 2.625 [2.577, 2.673] | 0.318 [0.266, 0.369] | 3270.0 | <.001 | <.001 | Yes |
| Claude 4.5 (FRES Prompt) | OSS-20b (Semantic) | 2.960 [2.941, 2.979] | 2.670 [2.624, 2.716] | 0.290 [0.243, 0.337] | 1687.5 | <.001 | <.001 | Yes |
| Claude 4.5 (Semantic) | OSS-20b (Semantic) | 2.958 [2.936, 2.979] | 2.670 [2.624, 2.716] | 0.287 [0.240, 0.335] | 1690.0 | <.001 | <.001 | Yes |
| GPT 5.2 (Semantic) | OSS-20b (FRES Prompt) | 2.942 [2.920, 2.965] | 2.623 [2.573, 2.672] | 0.320 [0.267, 0.373] | 3580.5 | <.001 | <.001 | Yes |
| Claude 4.5 (Baseline) | OSS-20b (Semantic) | 2.950 [2.929, 2.971] | 2.670 [2.624, 2.716] | 0.280 [0.232, 0.328] | 2362.5 | <.001 | <.001 | Yes |
| Gemini 3 (Semantic) | OSS-20b (Textstat) | 2.917 [2.890, 2.945] | 2.625 [2.577, 2.673] | 0.292 [0.242, 0.343] | 3641.0 | <.001 | <.001 | Yes |
| GPT 5.2 (Baseline) | OSS-20b (Textstat) | 2.910 [2.882, 2.938] | 2.625 [2.577, 2.673] | 0.285 [0.234, 0.336] | 3978.0 | <.001 | <.001 | Yes |
| GPT 5.2 (Baseline) | OSS-20b (FRES Prompt) | 2.910 [2.882, 2.938] | 2.623 [2.573, 2.672] | 0.287 [0.235, 0.340] | 3652.0 | <.001 | <.001 | Yes |
| Claude 4.5 (Textstat) | OSS-20b (Textstat) | 2.897 [2.867, 2.928] | 2.625 [2.577, 2.673] | 0.273 [0.223, 0.322] | 3360.0 | <.001 | <.001 | Yes |
| GPT 5.2 (Semantic) | OSS-20b (Semantic) | 2.942 [2.920, 2.965] | 2.670 [2.624, 2.716] | 0.273 [0.223, 0.322] | 3685.0 | <.001 | <.001 | Yes |
| Gemini 3 (Baseline) | OSS-20b (Textstat) | 2.913 [2.885, 2.940] | 2.625 [2.577, 2.673] | 0.287 [0.235, 0.340] | 4920.0 | <.001 | <.001 | Yes |
| Claude 4.5 (Textstat) | OSS-20b (FRES Prompt) | 2.897 [2.867, 2.928] | 2.623 [2.573, 2.672] | 0.275 [0.224, 0.326] | 3355.0 | <.001 | <.001 | Yes |
| Gemini 3 (Semantic) | OSS-20b (FRES Prompt) | 2.917 [2.890, 2.945] | 2.623 [2.573, 2.672] | 0.295 [0.240, 0.350] | 5823.0 | <.001 | <.001 | Yes |
| Gemini 3 (Baseline) | OSS-20b (FRES Prompt) | 2.913 [2.885, 2.940] | 2.623 [2.573, 2.672] | 0.290 [0.235, 0.345] | 5841.0 | <.001 | <.001 | Yes |
| GPT 5.2 (Baseline) | OSS-20b (Semantic) | 2.910 [2.882, 2.938] | 2.670 [2.624, 2.716] | 0.240 [0.192, 0.288] | 3756.5 | <.001 | <.001 | Yes |
| Gemini 3 (Semantic) | OSS-20b (Semantic) | 2.917 [2.890, 2.945] | 2.670 [2.624, 2.716] | 0.247 [0.197, 0.298] | 5360.0 | <.001 | <.001 | Yes |
| Gemini 3 (Baseline) | OSS-20b (Semantic) | 2.913 [2.885, 2.940] | 2.670 [2.624, 2.716] | 0.242 [0.192, 0.293] | 5055.0 | <.001 | <.001 | Yes |
| GPT 5.2 (FRES Prompt) | OSS-20b (FRES Prompt) | 2.890 [2.859, 2.921] | 2.623 [2.573, 2.672] | 0.268 [0.212, 0.323] | 6846.0 | <.001 | <.001 | Yes |
| GPT 5.2 (FRES Prompt) | OSS-20b (Textstat) | 2.890 [2.859, 2.921] | 2.625 [2.577, 2.673] | 0.265 [0.209, 0.321] | 7764.0 | <.001 | <.001 | Yes |
| Claude 4.5 (FRES Prompt) | OSS-20b (Baseline) | 2.960 [2.941, 2.979] | 2.763 [2.721, 2.804] | 0.198 [0.155, 0.240] | 1780.0 | <.001 | <.001 | Yes |
| Claude 4.5 (Textstat) | OSS-20b (Semantic) | 2.897 [2.867, 2.928] | 2.670 [2.624, 2.716] | 0.228 [0.177, 0.278] | 6066.0 | <.001 | <.001 | Yes |
| Claude 4.5 (Baseline) | OSS-20b (Baseline) | 2.950 [2.929, 2.971] | 2.763 [2.721, 2.804] | 0.188 [0.145, 0.230] | 2142.0 | <.001 | <.001 | Yes |
| Claude 4.5 (Semantic) | OSS-20b (Baseline) | 2.958 [2.936, 2.979] | 2.763 [2.721, 2.804] | 0.195 [0.151, 0.239] | 3172.5 | <.001 | <.001 | Yes |
| GPT 5.2 (FRES Prompt) | OSS-20b (Semantic) | 2.890 [2.859, 2.921] | 2.670 [2.624, 2.716] | 0.220 [0.167, 0.273] | 7670.5 | <.001 | <.001 | Yes |
| GPT 5.2 (Semantic) | OSS-20b (Baseline) | 2.942 [2.920, 2.965] | 2.763 [2.721, 2.804] | 0.180 [0.135, 0.225] | 4569.5 | <.001 | <.001 | Yes |
| Claude 4.5 (Semantic) | Gemini 3 (Textstat) | 2.958 [2.936, 2.979] | 2.795 [2.755, 2.835] | 0.163 [0.120, 0.205] | 2913.0 | <.001 | <.001 | Yes |
| Claude 4.5 (FRES Prompt) | Gemini 3 (Textstat) | 2.960 [2.941, 2.979] | 2.795 [2.755, 2.835] | 0.165 [0.122, 0.208] | 3575.0 | <.001 | <.001 | Yes |
| Claude 4.5 (FRES Prompt) | GPT 5.2 (Textstat) | 2.960 [2.941, 2.979] | 2.800 [2.761, 2.839] | 0.160 [0.118, 0.202] | 3585.0 | <.001 | <.001 | Yes |
| Claude 4.5 (Semantic) | GPT 5.2 (Textstat) | 2.958 [2.936, 2.979] | 2.800 [2.761, 2.839] | 0.158 [0.115, 0.200] | 3590.0 | <.001 | <.001 | Yes |
| Claude 4.5 (Baseline) | Gemini 3 (Textstat) | 2.950 [2.929, 2.971] | 2.795 [2.755, 2.835] | 0.155 [0.113, 0.197] | 3943.5 | <.001 | <.001 | Yes |
| Gemini 3 (FRES Prompt) | OSS-20b (Textstat) | 2.815 [2.777, 2.853] | 2.625 [2.577, 2.673] | 0.190 [0.138, 0.242] | 8437.5 | <.001 | <.001 | Yes |
| Claude 4.5 (Baseline) | GPT 5.2 (Textstat) | 2.950 [2.929, 2.971] | 2.800 [2.761, 2.839] | 0.150 [0.109, 0.191] | 3605.0 | <.001 | <.001 | Yes |
| GPT 5.2 (Semantic) | GPT 5.2 (Textstat) | 2.942 [2.920, 2.965] | 2.800 [2.761, 2.839] | 0.142 [0.102, 0.183] | 3267.0 | <.001 | <.001 | Yes |
| Claude 4.5 (FRES Prompt) | Gemini 3 (FRES Prompt) | 2.960 [2.941, 2.979] | 2.815 [2.777, 2.853] | 0.145 [0.104, 0.186] | 3615.0 | <.001 | <.001 | Yes |
| Gemini 3 (Textstat) | GPT 5.2 (Semantic) | 2.795 [2.755, 2.835] | 2.942 [2.920, 2.965] | -0.147 [-0.189, -0.106] | 3960.0 | <.001 | <.001 | Yes |
| GPT 5.2 (Textstat) | OSS-20b (Textstat) | 2.800 [2.761, 2.839] | 2.625 [2.577, 2.673] | 0.175 [0.125, 0.225] | 7557.0 | <.001 | <.001 | Yes |
| Gemini 3 (Semantic) | OSS-20b (Baseline) | 2.917 [2.890, 2.945] | 2.763 [2.721, 2.804] | 0.155 [0.110, 0.200] | 5317.5 | <.001 | <.001 | Yes |
| Gemini 3 (Textstat) | OSS-20b (Textstat) | 2.795 [2.755, 2.835] | 2.625 [2.577, 2.673] | 0.170 [0.121, 0.219] | 7255.5 | <.001 | <.001 | Yes |
| Claude 4.5 (Semantic) | Gemini 3 (FRES Prompt) | 2.958 [2.936, 2.979] | 2.815 [2.777, 2.853] | 0.142 [0.099, 0.186] | 4349.0 | <.001 | <.001 | Yes |
| GPT 5.2 (Baseline) | OSS-20b (Baseline) | 2.910 [2.882, 2.938] | 2.763 [2.721, 2.804] | 0.147 [0.104, 0.191] | 4998.0 | <.001 | <.001 | Yes |
| Gemini 3 (FRES Prompt) | OSS-20b (FRES Prompt) | 2.815 [2.777, 2.853] | 2.623 [2.573, 2.672] | 0.193 [0.136, 0.249] | 10,560.0 | <.001 | <.001 | Yes |
| Claude 4.5 (Baseline) | Gemini 3 (FRES Prompt) | 2.950 [2.929, 2.971] | 2.815 [2.777, 2.853] | 0.135 [0.094, 0.176] | 3987.5 | <.001 | <.001 | Yes |
| Gemini 3 (Baseline) | OSS-20b (Baseline) | 2.913 [2.885, 2.940] | 2.763 [2.721, 2.804] | 0.150 [0.103, 0.197] | 7339.5 | <.001 | <.001 | Yes |
| Gemini 3 (FRES Prompt) | GPT 5.2 (Semantic) | 2.815 [2.777, 2.853] | 2.942 [2.920, 2.965] | -0.128 [-0.168, -0.087] | 4356.0 | <.001 | <.001 | Yes |
| GPT 5.2 (Textstat) | OSS-20b (FRES Prompt) | 2.800 [2.761, 2.839] | 2.623 [2.573, 2.672] | 0.177 [0.121, 0.234] | 10,656.0 | <.001 | <.001 | Yes |
| Gemini 3 (Textstat) | OSS-20b (FRES Prompt) | 2.795 [2.755, 2.835] | 2.623 [2.573, 2.672] | 0.172 [0.116, 0.229] | 11,880.0 | <.001 | <.001 | Yes |
| Claude 4.5 (Textstat) | OSS-20b (Baseline) | 2.897 [2.867, 2.928] | 2.763 [2.721, 2.804] | 0.135 [0.089, 0.181] | 6763.0 | <.001 | <.001 | Yes |
| Gemini 3 (Semantic) | Gemini 3 (Textstat) | 2.917 [2.890, 2.945] | 2.795 [2.755, 2.835] | 0.122 [0.080, 0.165] | 5415.0 | <.001 | <.001 | Yes |
| GPT 5.2 (Textstat) | GPT 5.2 (Baseline) | 2.800 [2.761, 2.839] | 2.910 [2.882, 2.938] | -0.110 [-0.148, -0.072] | 3685.0 | <.001 | <.001 | Yes |
| OSS-20b (FRES Prompt) | OSS-20b (Baseline) | 2.623 [2.573, 2.672] | 2.763 [2.721, 2.804] | -0.140 [-0.190, -0.090] | 9328.5 | <.001 | <.001 | Yes |
| Gemini 3 (Textstat) | Gemini 3 (Baseline) | 2.795 [2.755, 2.835] | 2.913 [2.885, 2.940] | -0.117 [-0.160, -0.075] | 6120.0 | <.001 | <.001 | Yes |
| GPT 5.2 (FRES Prompt) | OSS-20b (Baseline) | 2.890 [2.859, 2.921] | 2.763 [2.721, 2.804] | 0.128 [0.080, 0.175] | 8750.0 | <.001 | <.001 | Yes |
| Gemini 3 (Textstat) | GPT 5.2 (Baseline) | 2.795 [2.755, 2.835] | 2.910 [2.882, 2.938] | -0.115 [-0.159, -0.071] | 6811.5 | <.001 | <.001 | Yes |
| Gemini 3 (Baseline) | GPT 5.2 (Textstat) | 2.913 [2.885, 2.940] | 2.800 [2.761, 2.839] | 0.113 [0.070, 0.155] | 6480.0 | <.001 | <.001 | Yes |
| Gemini 3 (Semantic) | GPT 5.2 (Textstat) | 2.917 [2.890, 2.945] | 2.800 [2.761, 2.839] | 0.117 [0.073, 0.162] | 7476.0 | <.001 | <.001 | Yes |
| OSS-20b (Textstat) | OSS-20b (Baseline) | 2.625 [2.577, 2.673] | 2.763 [2.721, 2.804] | -0.138 [-0.191, -0.084] | 11,830.0 | <.001 | <.001 | Yes |
| Claude 4.5 (Textstat) | Gemini 3 (Textstat) | 2.897 [2.867, 2.928] | 2.795 [2.755, 2.835] | 0.102 [0.062, 0.143] | 5475.0 | <.001 | <.001 | Yes |
| Gemini 3 (FRES Prompt) | OSS-20b (Semantic) | 2.815 [2.777, 2.853] | 2.670 [2.624, 2.716] | 0.145 [0.088, 0.202] | 14,125.5 | <.001 | <.001 | Yes |
| Gemini 3 (FRES Prompt) | Gemini 3 (Semantic) | 2.815 [2.777, 2.853] | 2.917 [2.890, 2.945] | -0.102 [-0.143, -0.062] | 5824.0 | <.001 | <.001 | Yes |
| Gemini 3 (FRES Prompt) | Gemini 3 (Baseline) | 2.815 [2.777, 2.853] | 2.913 [2.885, 2.940] | -0.098 [-0.138, -0.057] | 5840.0 | <.001 | <.001 | Yes |
| GPT 5.2 (Textstat) | OSS-20b (Semantic) | 2.800 [2.761, 2.839] | 2.670 [2.624, 2.716] | 0.130 [0.074, 0.186] | 1,3673.5 | <.001 | <.001 | Yes |
| Claude 4.5 (Textstat) | GPT 5.2 (Textstat) | 2.897 [2.867, 2.928] | 2.800 [2.761, 2.839] | 0.098 [0.055, 0.140] | 6878.0 | <.001 | <.001 | Yes |
| Gemini 3 (Textstat) | OSS-20b (Semantic) | 2.795 [2.755, 2.835] | 2.670 [2.624, 2.716] | 0.125 [0.068, 0.182] | 15,157.0 | <.001 | .001 | Yes |
| Claude 4.5 (FRES Prompt) | Claude 4.5 (Textstat) | 2.960 [2.941, 2.979] | 2.897 [2.867, 2.928] | 0.062 [0.033, 0.092] | 2292.0 | <.001 | .002 | Yes |
| Gemini 3 (FRES Prompt) | GPT 5.2 (Baseline) | 2.815 [2.777, 2.853] | 2.910 [2.882, 2.938] | -0.095 [-0.140, -0.050] | 8580.0 | <.001 | .002 | Yes |
| Claude 4.5 (FRES Prompt) | GPT 5.2 (FRES Prompt) | 2.960 [2.941, 2.979] | 2.890 [2.859, 2.921] | 0.070 [0.037, 0.103] | 3765.0 | <.001 | .003 | Yes |
| Gemini 3 (Textstat) | GPT 5.2 (FRES Prompt) | 2.795 [2.755, 2.835] | 2.890 [2.859, 2.921] | -0.095 [-0.141, -0.049] | 9571.5 | <.001 | .004 | Yes |
| Claude 4.5 (Textstat) | Gemini 3 (FRES Prompt) | 2.897 [2.867, 2.928] | 2.815 [2.777, 2.853] | 0.083 [0.040, 0.125] | 7297.0 | <.001 | .004 | Yes |
| GPT 5.2 (FRES Prompt) | GPT 5.2 (Textstat) | 2.890 [2.859, 2.921] | 2.800 [2.761, 2.839] | 0.090 [0.046, 0.134] | 8604.0 | <.001 | .004 | Yes |
| Claude 4.5 (Semantic) | Claude 4.5 (Textstat) | 2.958 [2.936, 2.979] | 2.897 [2.867, 2.928] | 0.060 [0.030, 0.090] | 2670.5 | <.001 | .004 | Yes |
| Claude 4.5 (Semantic) | GPT 5.2 (FRES Prompt) | 2.958 [2.936, 2.979] | 2.890 [2.859, 2.921] | 0.068 [0.032, 0.103] | 4514.0 | <.001 | .005 | Yes |
| Claude 4.5 (Textstat) | Claude 4.5 (Baseline) | 2.897 [2.867, 2.928] | 2.950 [2.929, 2.971] | -0.052 [-0.080, -0.025] | 2304.0 | <.001 | .01 | Yes |
| OSS-20b (Semantic) | OSS-20b (Baseline) | 2.670 [2.624, 2.716] | 2.763 [2.721, 2.804] | -0.092 [-0.143, -0.042] | 12,145.0 | <.001 | .02 | Yes |
| Claude 4.5 (Baseline) | GPT 5.2 (FRES Prompt) | 2.950 [2.929, 2.971] | 2.890 [2.859, 2.921] | 0.060 [0.027, 0.093] | 4152.5 | <.001 | .02 | Yes |
| Claude 4.5 (FRES Prompt) | GPT 5.2 (Baseline) | 2.960 [2.941, 2.979] | 2.910 [2.882, 2.938] | 0.050 [0.019, 0.081] | 3805.0 | .002 | .06 | No |
| Gemini 3 (FRES Prompt) | GPT 5.2 (FRES Prompt) | 2.815 [2.777, 2.853] | 2.890 [2.859, 2.921] | -0.075 [-0.122, -0.028] | 11,312.0 | .002 | .08 | No |
| GPT 5.2 (FRES Prompt) | GPT 5.2 (Semantic) | 2.890 [2.859, 2.921] | 2.942 [2.920, 2.965] | -0.052 [-0.086, -0.019] | 4901.0 | .002 | .08 | No |
| Claude 4.5 (Semantic) | GPT 5.2 (Baseline) | 2.958 [2.936, 2.979] | 2.910 [2.882, 2.938] | 0.048 [0.016, 0.079] | 4180.0 | .003 | .11 | No |
| Claude 4.5 (FRES Prompt) | Gemini 3 (Baseline) | 2.960 [2.941, 2.979] | 2.913 [2.885, 2.940] | 0.048 [0.015, 0.080] | 4914.0 | .005 | .17 | No |
| Claude 4.5 (Semantic) | Gemini 3 (Baseline) | 2.958 [2.936, 2.979] | 2.913 [2.885, 2.940] | 0.045 [0.011, 0.079] | 4930.0 | .005 | .17 | No |
| Claude 4.5 (Semantic) | Gemini 3 (Semantic) | 2.958 [2.936, 2.979] | 2.917 [2.890, 2.945] | 0.040 [0.008, 0.072] | 4574.5 | .008 | .28 | No |
| Claude 4.5 (Baseline) | GPT 5.2 (Baseline) | 2.950 [2.929, 2.971] | 2.910 [2.882, 2.938] | 0.040 [0.010, 0.070] | 4196.5 | .009 | .31 | No |
| Claude 4.5 (FRES Prompt) | Gemini 3 (Semantic) | 2.960 [2.941, 2.979] | 2.917 [2.890, 2.945] | 0.043 [0.011, 0.074] | 4927.0 | .01 | .31 | No |
| Claude 4.5 (Textstat) | GPT 5.2 (Semantic) | 2.897 [2.867, 2.928] | 2.942 [2.920, 2.965] | -0.045 [-0.080, -0.010] | 5647.5 | .01 | .40 | No |
| Claude 4.5 (Baseline) | Gemini 3 (Baseline) | 2.950 [2.929, 2.971] | 2.913 [2.885, 2.940] | 0.037 [0.006, 0.069] | 4940.0 | .02 | .57 | No |
| Claude 4.5 (Baseline) | Gemini 3 (Semantic) | 2.950 [2.929, 2.971] | 2.917 [2.890, 2.945] | 0.033 [0.003, 0.062] | 4584.0 | .03 | .95 | No |
| Claude 4.5 (Baseline) | GPT 5.2 (Semantic) | 2.950 [2.929, 2.971] | 2.942 [2.920, 2.965] | 0.007 [-0.020, 0.035] | 5390.0 | .59 | >.99 | No |
| Claude 4.5 (FRES Prompt) | Claude 4.5 (Semantic) | 2.960 [2.941, 2.979] | 2.958 [2.936, 2.979] | 0.003 [-0.021, 0.026] | 4279.0 | .84 | >.99 | No |
| Claude 4.5 (FRES Prompt) | Claude 4.5 (Baseline) | 2.960 [2.941, 2.979] | 2.950 [2.929, 2.971] | 0.010 [-0.011, 0.031] | 2740.5 | .35 | >.99 | No |
| Claude 4.5 (FRES Prompt) | GPT 5.2 (Semantic) | 2.960 [2.941, 2.979] | 2.942 [2.920, 2.965] | 0.018 [-0.010, 0.045] | 4620.0 | .21 | >.99 | No |
| Claude 4.5 (Semantic) | Claude 4.5 (Baseline) | 2.958 [2.936, 2.979] | 2.950 [2.929, 2.971] | 0.007 [-0.016, 0.031] | 3890.0 | .53 | >.99 | No |
| Claude 4.5 (Semantic) | GPT 5.2 (Semantic) | 2.958 [2.936, 2.979] | 2.942 [2.920, 2.965] | 0.015 [-0.016, 0.046] | 5741.0 | .25 | >.99 | No |
| Claude 4.5 (Textstat) | Gemini 3 (Semantic) | 2.897 [2.867, 2.928] | 2.917 [2.890, 2.945] | -0.020 [-0.058, 0.018] | 9287.5 | .35 | >.99 | No |
| Claude 4.5 (Textstat) | Gemini 3 (Baseline) | 2.897 [2.867, 2.928] | 2.913 [2.885, 2.940] | -0.015 [-0.051, 0.021] | 8613.5 | .48 | >.99 | No |
| Claude 4.5 (Textstat) | GPT 5.2 (FRES Prompt) | 2.897 [2.867, 2.928] | 2.890 [2.859, 2.921] | 0.007 [-0.032, 0.047] | 11,070.0 | .70 | >.99 | No |
| Claude 4.5 (Textstat) | GPT 5.2 (Baseline) | 2.897 [2.867, 2.928] | 2.910 [2.882, 2.938] | -0.013 [-0.048, 0.023] | 8976.0 | .49 | >.99 | No |
| GPT 5.2 (FRES Prompt) | GPT 5.2 (Baseline) | 2.890 [2.859, 2.921] | 2.910 [2.882, 2.938] | -0.020 [-0.056, 0.016] | 8590.5 | .28 | >.99 | No |
| GPT 5.2 (Semantic) | GPT 5.2 (Baseline) | 2.942 [2.920, 2.965] | 2.910 [2.882, 2.938] | 0.033 [0.002, 0.063] | 4953.0 | .04 | >.99 | No |
| GPT 5.2 (Textstat) | OSS-20b (Baseline) | 2.800 [2.761, 2.839] | 2.763 [2.721, 2.804] | 0.037 [-0.012, 0.087] | 15,050.0 | .14 | >.99 | No |
| Gemini 3 (Baseline) | GPT 5.2 (FRES Prompt) | 2.913 [2.885, 2.940] | 2.890 [2.859, 2.921] | 0.022 [-0.015, 0.060] | 9275.0 | .24 | >.99 | No |
| Gemini 3 (Baseline) | GPT 5.2 (Semantic) | 2.913 [2.885, 2.940] | 2.942 [2.920, 2.965] | -0.030 [-0.063, 0.003] | 6056.0 | .07 | >.99 | No |
| Gemini 3 (Baseline) | GPT 5.2 (Baseline) | 2.913 [2.885, 2.940] | 2.910 [2.882, 2.938] | 0.003 [-0.033, 0.038] | 9724.0 | .89 | >.99 | No |
| Gemini 3 (FRES Prompt) | Gemini 3 (Textstat) | 2.815 [2.777, 2.853] | 2.795 [2.755, 2.835] | 0.020 [-0.020, 0.060] | 10,657.5 | .33 | >.99 | No |
| Gemini 3 (FRES Prompt) | GPT 5.2 (Textstat) | 2.815 [2.777, 2.853] | 2.800 [2.761, 2.839] | 0.015 [-0.030, 0.060] | 13,981.5 | .51 | >.99 | No |
| Gemini 3 (FRES Prompt) | OSS-20b (Baseline) | 2.815 [2.777, 2.853] | 2.763 [2.721, 2.804] | 0.052 [0.000, 0.105] | 15,824.0 | .048 | >.99 | No |
| Gemini 3 (Semantic) | Gemini 3 (Baseline) | 2.917 [2.890, 2.945] | 2.913 [2.885, 2.940] | 0.005 [-0.028, 0.038] | 8305.0 | .77 | >.99 | No |
| Gemini 3 (Semantic) | GPT 5.2 (FRES Prompt) | 2.917 [2.890, 2.945] | 2.890 [2.859, 2.921] | 0.028 [-0.008, 0.063] | 7854.0 | .13 | >.99 | No |
| Gemini 3 (Semantic) | GPT 5.2 (Semantic) | 2.917 [2.890, 2.945] | 2.942 [2.920, 2.965] | -0.025 [-0.060, 0.010] | 7510.0 | .16 | >.99 | No |
| Gemini 3 (Semantic) | GPT 5.2 (Baseline) | 2.917 [2.890, 2.945] | 2.910 [2.882, 2.938] | 0.007 [-0.029, 0.044] | 9698.0 | .69 | >.99 | No |
| Gemini 3 (Textstat) | GPT 5.2 (Textstat) | 2.795 [2.755, 2.835] | 2.800 [2.761, 2.839] | -0.005 [-0.047, 0.037] | 13,086.0 | .82 | >.99 | No |
| Gemini 3 (Textstat) | OSS-20b (Baseline) | 2.795 [2.755, 2.835] | 2.763 [2.721, 2.804] | 0.033 [-0.019, 0.084] | 16,905.0 | .22 | >.99 | No |
| OSS-20b (FRES Prompt) | OSS-20b (Semantic) | 2.623 [2.573, 2.672] | 2.670 [2.624, 2.716] | -0.048 [-0.104, 0.009] | 18,760.0 | .13 | >.99 | No |
| OSS-20b (FRES Prompt) | OSS-20b (Textstat) | 2.623 [2.573, 2.672] | 2.625 [2.577, 2.673] | -0.003 [-0.054, 0.049] | 17,553.0 | .88 | >.99 | No |
| OSS-20b (Semantic) | OSS-20b (Textstat) | 2.670 [2.624, 2.716] | 2.625 [2.577, 2.673] | 0.045 [-0.012, 0.102] | 19,343.0 | .12 | >.99 | No |

Completeness was rated on an ordinal scale from 1 to 3. Means and 95% CIs are presented as supplementary descriptive measures because medians showed a strong ceiling effect. Mean-score and paired mean-difference 95% CIs were calculated using the Student t distribution. Pairwise comparisons used 2-sided Wilcoxon signed-rank tests with the Pratt method for zero differences, with P values adjusted across all 120 comparisons using the Holm procedure. P values are reported to 2 decimal places, except when P<.01 or when rounding would alter the interpretation of statistical significance. Values below .001 are reported as P<.001, and values above .99 as P>.99. Statistical significance was defined as Holm-adjusted P<.05. Praw indicates the unadjusted P value; PHolm, the Holm-adjusted P value; and Holm sig., statistical significance after Holm correction. Abbreviation: CI, confidence interval.
