# Supplementary material for: Prompting Strategies for Readability Alignment Between User-Generated Ophthalmology Questions and Large Language Model Answers: Comparative Evaluation Study
Source: JMIR Form Res. 2026 Nov 5;10:e97408. doi: 10.2196/97408 (PMC13739205; doi:10.2196/97408)
Supplement: Supplementary file 9 [file formative-v10-e97408-s009.docx]

**Pairwise comparisons of empathy scores with Holm adjustment.**

| **Condition A** | **Condition B** | **Mean A (95% CI)** | **Mean B (95% CI)** | **Mean difference (95% CI)** | **Wilcoxon W** | ***P*raw** | ***P*Holm** | **Holm sig.** |
| --- | --- | --- | --- | --- | --- | --- | --- | --- |
| Claude 4.5 (Textstat) | GPT 5.2 (FRES Prompt) | 2.788 [2.747, 2.828] | 2.110 [2.079, 2.141] | 0.677 [0.631, 0.724] | 264.0 | <.001 | <.001 | Yes |
| Claude 4.5 (Textstat) | GPT 5.2 (Baseline) | 2.788 [2.747, 2.828] | 2.103 [2.073, 2.132] | 0.685 [0.638, 0.732] | 781.5 | <.001 | <.001 | Yes |
| Claude 4.5 (Textstat) | GPT 5.2 (Textstat) | 2.788 [2.747, 2.828] | 2.125 [2.092, 2.158] | 0.662 [0.613, 0.712] | 1315.0 | <.001 | <.001 | Yes |
| Claude 4.5 (Textstat) | GPT 5.2 (Semantic) | 2.788 [2.747, 2.828] | 2.132 [2.099, 2.166] | 0.655 [0.606, 0.704] | 1322.5 | <.001 | <.001 | Yes |
| Claude 4.5 (FRES Prompt) | GPT 5.2 (Baseline) | 2.735 [2.692, 2.778] | 2.103 [2.073, 2.132] | 0.632 [0.584, 0.681] | 544.0 | <.001 | <.001 | Yes |
| Claude 4.5 (FRES Prompt) | GPT 5.2 (FRES Prompt) | 2.735 [2.692, 2.778] | 2.110 [2.079, 2.141] | 0.625 [0.575, 0.675] | 1086.0 | <.001 | <.001 | Yes |
| Gemini 3 (Baseline) | GPT 5.2 (Baseline) | 2.723 [2.678, 2.767] | 2.103 [2.073, 2.132] | 0.620 [0.570, 0.670] | 1357.5 | <.001 | <.001 | Yes |
| Gemini 3 (Baseline) | GPT 5.2 (FRES Prompt) | 2.723 [2.678, 2.767] | 2.110 [2.079, 2.141] | 0.613 [0.563, 0.662] | 1096.0 | <.001 | <.001 | Yes |
| Claude 4.5 (Baseline) | GPT 5.2 (Baseline) | 2.717 [2.673, 2.762] | 2.103 [2.073, 2.132] | 0.615 [0.565, 0.665] | 1362.5 | <.001 | <.001 | Yes |
| Claude 4.5 (Baseline) | GPT 5.2 (FRES Prompt) | 2.717 [2.673, 2.762] | 2.110 [2.079, 2.141] | 0.608 [0.557, 0.658] | 1638.0 | <.001 | <.001 | Yes |
| Claude 4.5 (FRES Prompt) | GPT 5.2 (Textstat) | 2.735 [2.692, 2.778] | 2.125 [2.092, 2.158] | 0.610 [0.558, 0.662] | 2164.0 | <.001 | <.001 | Yes |
| Claude 4.5 (FRES Prompt) | GPT 5.2 (Semantic) | 2.735 [2.692, 2.778] | 2.132 [2.099, 2.166] | 0.603 [0.551, 0.654] | 1911.0 | <.001 | <.001 | Yes |
| Claude 4.5 (Baseline) | GPT 5.2 (Textstat) | 2.717 [2.673, 2.762] | 2.125 [2.092, 2.158] | 0.593 [0.541, 0.644] | 1656.0 | <.001 | <.001 | Yes |
| Gemini 3 (Baseline) | GPT 5.2 (Semantic) | 2.723 [2.678, 2.767] | 2.132 [2.099, 2.166] | 0.590 [0.538, 0.642] | 1928.5 | <.001 | <.001 | Yes |
| Claude 4.5 (Baseline) | GPT 5.2 (Semantic) | 2.717 [2.673, 2.762] | 2.132 [2.099, 2.166] | 0.585 [0.533, 0.637] | 2204.0 | <.001 | <.001 | Yes |
| Gemini 3 (Baseline) | GPT 5.2 (Textstat) | 2.723 [2.678, 2.767] | 2.125 [2.092, 2.158] | 0.598 [0.544, 0.651] | 3228.0 | <.001 | <.001 | Yes |
| Gemini 3 (FRES Prompt) | GPT 5.2 (Baseline) | 2.647 [2.600, 2.695] | 2.103 [2.073, 2.132] | 0.545 [0.495, 0.595] | 865.5 | <.001 | <.001 | Yes |
| Gemini 3 (FRES Prompt) | GPT 5.2 (FRES Prompt) | 2.647 [2.600, 2.695] | 2.110 [2.079, 2.141] | 0.537 [0.485, 0.590] | 2280.0 | <.001 | <.001 | Yes |
| Claude 4.5 (Textstat) | OSS-20b (Semantic) | 2.788 [2.747, 2.828] | 2.272 [2.229, 2.316] | 0.515 [0.464, 0.566] | 1174.0 | <.001 | <.001 | Yes |
| Gemini 3 (Textstat) | GPT 5.2 (Baseline) | 2.618 [2.570, 2.665] | 2.103 [2.073, 2.132] | 0.515 [0.462, 0.568] | 2596.5 | <.001 | <.001 | Yes |
| Gemini 3 (Semantic) | GPT 5.2 (Baseline) | 2.603 [2.554, 2.651] | 2.103 [2.073, 2.132] | 0.500 [0.447, 0.553] | 2054.5 | <.001 | <.001 | Yes |
| Claude 4.5 (Textstat) | OSS-20b (Textstat) | 2.788 [2.747, 2.828] | 2.297 [2.253, 2.342] | 0.490 [0.438, 0.542] | 1487.5 | <.001 | <.001 | Yes |
| Gemini 3 (FRES Prompt) | GPT 5.2 (Semantic) | 2.647 [2.600, 2.695] | 2.132 [2.099, 2.166] | 0.515 [0.461, 0.569] | 3151.5 | <.001 | <.001 | Yes |
| Gemini 3 (FRES Prompt) | GPT 5.2 (Textstat) | 2.647 [2.600, 2.695] | 2.125 [2.092, 2.158] | 0.522 [0.467, 0.578] | 3679.0 | <.001 | <.001 | Yes |
| Gemini 3 (Textstat) | GPT 5.2 (FRES Prompt) | 2.618 [2.570, 2.665] | 2.110 [2.079, 2.141] | 0.507 [0.454, 0.561] | 2890.0 | <.001 | <.001 | Yes |
| Claude 4.5 (Semantic) | GPT 5.2 (Baseline) | 2.600 [2.552, 2.648] | 2.103 [2.073, 2.132] | 0.497 [0.445, 0.550] | 2344.0 | <.001 | <.001 | Yes |
| Gemini 3 (Semantic) | GPT 5.2 (FRES Prompt) | 2.603 [2.554, 2.651] | 2.110 [2.079, 2.141] | 0.492 [0.440, 0.545] | 2065.0 | <.001 | <.001 | Yes |
| Claude 4.5 (Semantic) | GPT 5.2 (FRES Prompt) | 2.600 [2.552, 2.648] | 2.110 [2.079, 2.141] | 0.490 [0.437, 0.543] | 2068.5 | <.001 | <.001 | Yes |
| Gemini 3 (Textstat) | GPT 5.2 (Textstat) | 2.618 [2.570, 2.665] | 2.125 [2.092, 2.158] | 0.492 [0.437, 0.548] | 4032.0 | <.001 | <.001 | Yes |
| Gemini 3 (Textstat) | GPT 5.2 (Semantic) | 2.618 [2.570, 2.665] | 2.132 [2.099, 2.166] | 0.485 [0.430, 0.540] | 3776.5 | <.001 | <.001 | Yes |
| Claude 4.5 (Semantic) | GPT 5.2 (Textstat) | 2.600 [2.552, 2.648] | 2.125 [2.092, 2.158] | 0.475 [0.421, 0.529] | 3239.5 | <.001 | <.001 | Yes |
| Claude 4.5 (FRES Prompt) | OSS-20b (Semantic) | 2.735 [2.692, 2.778] | 2.272 [2.229, 2.316] | 0.463 [0.409, 0.516] | 2980.0 | <.001 | <.001 | Yes |
| Gemini 3 (Semantic) | GPT 5.2 (Textstat) | 2.603 [2.554, 2.651] | 2.125 [2.092, 2.158] | 0.477 [0.421, 0.534] | 4350.0 | <.001 | <.001 | Yes |
| Claude 4.5 (Semantic) | GPT 5.2 (Semantic) | 2.600 [2.552, 2.648] | 2.132 [2.099, 2.166] | 0.468 [0.412, 0.523] | 3822.0 | <.001 | <.001 | Yes |
| Claude 4.5 (FRES Prompt) | OSS-20b (Textstat) | 2.735 [2.692, 2.778] | 2.297 [2.253, 2.342] | 0.438 [0.385, 0.490] | 2142.0 | <.001 | <.001 | Yes |
| Gemini 3 (Semantic) | GPT 5.2 (Semantic) | 2.603 [2.554, 2.651] | 2.132 [2.099, 2.166] | 0.470 [0.414, 0.526] | 4648.0 | <.001 | <.001 | Yes |
| Claude 4.5 (Baseline) | OSS-20b (Semantic) | 2.717 [2.673, 2.762] | 2.272 [2.229, 2.316] | 0.445 [0.391, 0.499] | 3305.5 | <.001 | <.001 | Yes |
| Gemini 3 (Baseline) | OSS-20b (Textstat) | 2.723 [2.678, 2.767] | 2.297 [2.253, 2.342] | 0.425 [0.372, 0.478] | 2758.5 | <.001 | <.001 | Yes |
| Claude 4.5 (Baseline) | OSS-20b (Textstat) | 2.717 [2.673, 2.762] | 2.297 [2.253, 2.342] | 0.420 [0.367, 0.473] | 3065.0 | <.001 | <.001 | Yes |
| Gemini 3 (Baseline) | OSS-20b (Semantic) | 2.723 [2.678, 2.767] | 2.272 [2.229, 2.316] | 0.450 [0.392, 0.508] | 5538.5 | <.001 | <.001 | Yes |
| Claude 4.5 (Textstat) | OSS-20b (FRES Prompt) | 2.788 [2.747, 2.828] | 2.368 [2.320, 2.415] | 0.420 [0.365, 0.475] | 3945.5 | <.001 | <.001 | Yes |
| Gemini 3 (FRES Prompt) | OSS-20b (Semantic) | 2.647 [2.600, 2.695] | 2.272 [2.229, 2.316] | 0.375 [0.319, 0.431] | 5244.5 | <.001 | <.001 | Yes |
| Claude 4.5 (FRES Prompt) | OSS-20b (FRES Prompt) | 2.735 [2.692, 2.778] | 2.368 [2.320, 2.415] | 0.367 [0.312, 0.423] | 4976.0 | <.001 | <.001 | Yes |
| Gemini 3 (FRES Prompt) | OSS-20b (Textstat) | 2.647 [2.600, 2.695] | 2.297 [2.253, 2.342] | 0.350 [0.297, 0.403] | 4127.5 | <.001 | <.001 | Yes |
| Claude 4.5 (Textstat) | OSS-20b (Baseline) | 2.788 [2.747, 2.828] | 2.438 [2.389, 2.486] | 0.350 [0.295, 0.405] | 5032.0 | <.001 | <.001 | Yes |
| Claude 4.5 (Baseline) | OSS-20b (FRES Prompt) | 2.717 [2.673, 2.762] | 2.368 [2.320, 2.415] | 0.350 [0.294, 0.406] | 5918.5 | <.001 | <.001 | Yes |
| GPT 5.2 (Baseline) | OSS-20b (Baseline) | 2.103 [2.073, 2.132] | 2.438 [2.389, 2.486] | -0.335 [-0.389, -0.281] | 4777.5 | <.001 | <.001 | Yes |
| Gemini 3 (Baseline) | OSS-20b (FRES Prompt) | 2.723 [2.678, 2.767] | 2.368 [2.320, 2.415] | 0.355 [0.297, 0.413] | 7049.5 | <.001 | <.001 | Yes |
| GPT 5.2 (FRES Prompt) | OSS-20b (Baseline) | 2.110 [2.079, 2.141] | 2.438 [2.389, 2.486] | -0.328 [-0.381, -0.274] | 4800.0 | <.001 | <.001 | Yes |
| Claude 4.5 (Semantic) | OSS-20b (Semantic) | 2.600 [2.552, 2.648] | 2.272 [2.229, 2.316] | 0.328 [0.273, 0.382] | 5406.0 | <.001 | <.001 | Yes |
| Gemini 3 (Textstat) | OSS-20b (Semantic) | 2.618 [2.570, 2.665] | 2.272 [2.229, 2.316] | 0.345 [0.288, 0.402] | 7095.5 | <.001 | <.001 | Yes |
| Gemini 3 (Semantic) | OSS-20b (Semantic) | 2.603 [2.554, 2.651] | 2.272 [2.229, 2.316] | 0.330 [0.274, 0.386] | 6290.0 | <.001 | <.001 | Yes |
| Gemini 3 (Textstat) | OSS-20b (Textstat) | 2.618 [2.570, 2.665] | 2.297 [2.253, 2.342] | 0.320 [0.264, 0.376] | 6625.5 | <.001 | <.001 | Yes |
| Gemini 3 (Semantic) | OSS-20b (Textstat) | 2.603 [2.554, 2.651] | 2.297 [2.253, 2.342] | 0.305 [0.251, 0.359] | 5787.0 | <.001 | <.001 | Yes |
| Claude 4.5 (FRES Prompt) | OSS-20b (Baseline) | 2.735 [2.692, 2.778] | 2.438 [2.389, 2.486] | 0.297 [0.245, 0.350] | 5200.0 | <.001 | <.001 | Yes |
| Claude 4.5 (Semantic) | OSS-20b (Textstat) | 2.600 [2.552, 2.648] | 2.297 [2.253, 2.342] | 0.302 [0.249, 0.356] | 5796.0 | <.001 | <.001 | Yes |
| GPT 5.2 (Textstat) | OSS-20b (Baseline) | 2.125 [2.092, 2.158] | 2.438 [2.389, 2.486] | -0.312 [-0.369, -0.256] | 7245.0 | <.001 | <.001 | Yes |
| GPT 5.2 (Semantic) | OSS-20b (Baseline) | 2.132 [2.099, 2.166] | 2.438 [2.389, 2.486] | -0.305 [-0.361, -0.249] | 7279.5 | <.001 | <.001 | Yes |
| Gemini 3 (Baseline) | OSS-20b (Baseline) | 2.723 [2.678, 2.767] | 2.438 [2.389, 2.486] | 0.285 [0.229, 0.341] | 7371.5 | <.001 | <.001 | Yes |
| Claude 4.5 (Baseline) | OSS-20b (Baseline) | 2.717 [2.673, 2.762] | 2.438 [2.389, 2.486] | 0.280 [0.223, 0.337] | 8281.0 | <.001 | <.001 | Yes |
| Gemini 3 (FRES Prompt) | OSS-20b (FRES Prompt) | 2.647 [2.600, 2.695] | 2.368 [2.320, 2.415] | 0.280 [0.223, 0.337] | 8572.5 | <.001 | <.001 | Yes |
| GPT 5.2 (Baseline) | OSS-20b (FRES Prompt) | 2.103 [2.073, 2.132] | 2.368 [2.320, 2.415] | -0.265 [-0.320, -0.210] | 7463.5 | <.001 | <.001 | Yes |
| GPT 5.2 (FRES Prompt) | OSS-20b (FRES Prompt) | 2.110 [2.079, 2.141] | 2.368 [2.320, 2.415] | -0.258 [-0.313, -0.202] | 8100.0 | <.001 | <.001 | Yes |
| GPT 5.2 (Textstat) | OSS-20b (FRES Prompt) | 2.125 [2.092, 2.158] | 2.368 [2.320, 2.415] | -0.242 [-0.297, -0.188] | 8175.0 | <.001 | <.001 | Yes |
| Gemini 3 (Textstat) | OSS-20b (FRES Prompt) | 2.618 [2.570, 2.665] | 2.368 [2.320, 2.415] | 0.250 [0.192, 0.308] | 10,477.5 | <.001 | <.001 | Yes |
| Claude 4.5 (Semantic) | Claude 4.5 (Textstat) | 2.600 [2.552, 2.648] | 2.788 [2.747, 2.828] | -0.188 [-0.233, -0.142] | 4212.0 | <.001 | <.001 | Yes |
| GPT 5.2 (Semantic) | OSS-20b (FRES Prompt) | 2.132 [2.099, 2.166] | 2.368 [2.320, 2.415] | -0.235 [-0.292, -0.178] | 9997.5 | <.001 | <.001 | Yes |
| GPT 5.2 (Baseline) | OSS-20b (Textstat) | 2.103 [2.073, 2.132] | 2.297 [2.253, 2.342] | -0.195 [-0.244, -0.146] | 6183.0 | <.001 | <.001 | Yes |
| Gemini 3 (Semantic) | OSS-20b (FRES Prompt) | 2.603 [2.554, 2.651] | 2.368 [2.320, 2.415] | 0.235 [0.175, 0.295] | 11,989.0 | <.001 | <.001 | Yes |
| Claude 4.5 (Semantic) | OSS-20b (FRES Prompt) | 2.600 [2.552, 2.648] | 2.368 [2.320, 2.415] | 0.233 [0.173, 0.292] | 12,008.0 | <.001 | <.001 | Yes |
| GPT 5.2 (FRES Prompt) | OSS-20b (Textstat) | 2.110 [2.079, 2.141] | 2.297 [2.253, 2.342] | -0.188 [-0.238, -0.137] | 7502.0 | <.001 | <.001 | Yes |
| Gemini 3 (FRES Prompt) | OSS-20b (Baseline) | 2.647 [2.600, 2.695] | 2.438 [2.389, 2.486] | 0.210 [0.153, 0.267] | 10,741.5 | <.001 | <.001 | Yes |
| Claude 4.5 (Textstat) | Gemini 3 (Textstat) | 2.788 [2.747, 2.828] | 2.618 [2.570, 2.665] | 0.170 [0.124, 0.216] | 5608.0 | <.001 | <.001 | Yes |
| Claude 4.5 (Textstat) | Gemini 3 (Semantic) | 2.788 [2.747, 2.828] | 2.603 [2.554, 2.651] | 0.185 [0.134, 0.236] | 7831.5 | <.001 | <.001 | Yes |
| GPT 5.2 (Baseline) | OSS-20b (Semantic) | 2.103 [2.073, 2.132] | 2.272 [2.229, 2.316] | -0.170 [-0.219, -0.121] | 7255.5 | <.001 | <.001 | Yes |
| GPT 5.2 (FRES Prompt) | OSS-20b (Semantic) | 2.110 [2.079, 2.141] | 2.272 [2.229, 2.316] | -0.163 [-0.211, -0.114] | 7612.0 | <.001 | <.001 | Yes |
| GPT 5.2 (Textstat) | OSS-20b (Textstat) | 2.125 [2.092, 2.158] | 2.297 [2.253, 2.342] | -0.172 [-0.225, -0.120] | 9464.0 | <.001 | <.001 | Yes |
| Gemini 3 (Textstat) | OSS-20b (Baseline) | 2.618 [2.570, 2.665] | 2.438 [2.389, 2.486] | 0.180 [0.123, 0.237] | 12,117.5 | <.001 | <.001 | Yes |
| Claude 4.5 (Textstat) | Gemini 3 (FRES Prompt) | 2.788 [2.747, 2.828] | 2.647 [2.600, 2.695] | 0.140 [0.094, 0.186] | 6716.5 | <.001 | <.001 | Yes |
| GPT 5.2 (Semantic) | OSS-20b (Textstat) | 2.132 [2.099, 2.166] | 2.297 [2.253, 2.342] | -0.165 [-0.219, -0.111] | 11,038.5 | <.001 | <.001 | Yes |
| GPT 5.2 (Textstat) | OSS-20b (Semantic) | 2.125 [2.092, 2.158] | 2.272 [2.229, 2.316] | -0.147 [-0.197, -0.098] | 8650.0 | <.001 | <.001 | Yes |
| OSS-20b (Semantic) | OSS-20b (Baseline) | 2.272 [2.229, 2.316] | 2.438 [2.389, 2.486] | -0.165 [-0.221, -0.109] | 12,228.5 | <.001 | <.001 | Yes |
| Gemini 3 (Semantic) | OSS-20b (Baseline) | 2.603 [2.554, 2.651] | 2.438 [2.389, 2.486] | 0.165 [0.108, 0.222] | 13,100.0 | <.001 | <.001 | Yes |
| Claude 4.5 (FRES Prompt) | Claude 4.5 (Semantic) | 2.735 [2.692, 2.778] | 2.600 [2.552, 2.648] | 0.135 [0.088, 0.182] | 8061.5 | <.001 | <.001 | Yes |
| Claude 4.5 (Semantic) | OSS-20b (Baseline) | 2.600 [2.552, 2.648] | 2.438 [2.389, 2.486] | 0.163 [0.105, 0.220] | 13,407.0 | <.001 | <.001 | Yes |
| GPT 5.2 (Semantic) | OSS-20b (Semantic) | 2.132 [2.099, 2.166] | 2.272 [2.229, 2.316] | -0.140 [-0.193, -0.087] | 11,203.5 | <.001 | <.001 | Yes |
| OSS-20b (Textstat) | OSS-20b (Baseline) | 2.297 [2.253, 2.342] | 2.438 [2.389, 2.486] | -0.140 [-0.193, -0.087] | 11,203.5 | <.001 | <.001 | Yes |
| Claude 4.5 (FRES Prompt) | Gemini 3 (Semantic) | 2.735 [2.692, 2.778] | 2.603 [2.554, 2.651] | 0.133 [0.081, 0.184] | 10,633.0 | <.001 | <.001 | Yes |
| Gemini 3 (Semantic) | Gemini 3 (Baseline) | 2.603 [2.554, 2.651] | 2.723 [2.678, 2.767] | -0.120 [-0.169, -0.071] | 9758.0 | <.001 | <.001 | Yes |
| Claude 4.5 (Semantic) | Gemini 3 (Baseline) | 2.600 [2.552, 2.648] | 2.723 [2.678, 2.767] | -0.122 [-0.173, -0.072] | 11,008.0 | <.001 | <.001 | Yes |
| Claude 4.5 (Semantic) | Claude 4.5 (Baseline) | 2.600 [2.552, 2.648] | 2.717 [2.673, 2.762] | -0.117 [-0.167, -0.068] | 10,092.0 | <.001 | <.001 | Yes |
| Claude 4.5 (FRES Prompt) | Gemini 3 (Textstat) | 2.735 [2.692, 2.778] | 2.618 [2.570, 2.665] | 0.117 [0.067, 0.168] | 10,726.0 | <.001 | <.001 | Yes |
| Claude 4.5 (Baseline) | Gemini 3 (Semantic) | 2.717 [2.673, 2.762] | 2.603 [2.554, 2.651] | 0.115 [0.065, 0.165] | 10,741.5 | <.001 | <.001 | Yes |
| Gemini 3 (Textstat) | Gemini 3 (Baseline) | 2.618 [2.570, 2.665] | 2.723 [2.678, 2.767] | -0.105 [-0.153, -0.057] | 10,164.5 | <.001 | <.001 | Yes |
| Claude 4.5 (Baseline) | Gemini 3 (Textstat) | 2.717 [2.673, 2.762] | 2.618 [2.570, 2.665] | 0.100 [0.050, 0.150] | 11,781.0 | <.001 | .003 | Yes |
| Claude 4.5 (FRES Prompt) | Gemini 3 (FRES Prompt) | 2.735 [2.692, 2.778] | 2.647 [2.600, 2.695] | 0.087 [0.039, 0.136] | 11,550.0 | <.001 | .01 | Yes |
| Claude 4.5 (Textstat) | Claude 4.5 (Baseline) | 2.788 [2.747, 2.828] | 2.717 [2.673, 2.762] | 0.070 [0.028, 0.112] | 8700.0 | .001 | .03 | Yes |
| OSS-20b (FRES Prompt) | OSS-20b (Semantic) | 2.368 [2.320, 2.415] | 2.272 [2.229, 2.316] | 0.095 [0.038, 0.152] | 16,855.5 | .001 | .03 | Yes |
| Gemini 3 (FRES Prompt) | Gemini 3 (Baseline) | 2.647 [2.600, 2.695] | 2.723 [2.678, 2.767] | -0.075 [-0.126, -0.024] | 13,513.5 | .004 | .09 | No |
| Claude 4.5 (Textstat) | Gemini 3 (Baseline) | 2.788 [2.747, 2.828] | 2.723 [2.678, 2.767] | 0.065 [0.021, 0.109] | 10,066.0 | .004 | .09 | No |
| Claude 4.5 (Baseline) | Gemini 3 (FRES Prompt) | 2.717 [2.673, 2.762] | 2.647 [2.600, 2.695] | 0.070 [0.020, 0.120] | 13,552.5 | .007 | .13 | No |
| Claude 4.5 (FRES Prompt) | Claude 4.5 (Textstat) | 2.735 [2.692, 2.778] | 2.788 [2.747, 2.828] | -0.052 [-0.095, -0.010] | 9801.0 | .02 | .28 | No |
| OSS-20b (FRES Prompt) | OSS-20b (Textstat) | 2.368 [2.320, 2.415] | 2.297 [2.253, 2.342] | 0.070 [0.014, 0.126] | 17,394.0 | .02 | .28 | No |
| OSS-20b (FRES Prompt) | OSS-20b (Baseline) | 2.368 [2.320, 2.415] | 2.438 [2.389, 2.486] | -0.070 [-0.126, -0.014] | 17,394.0 | .02 | .28 | No |
| Claude 4.5 (Baseline) | Gemini 3 (Baseline) | 2.717 [2.673, 2.762] | 2.723 [2.678, 2.767] | -0.005 [-0.054, 0.044] | 16,872.0 | .84 | >.99 | No |
| Claude 4.5 (FRES Prompt) | Claude 4.5 (Baseline) | 2.735 [2.692, 2.778] | 2.717 [2.673, 2.762] | 0.018 [-0.029, 0.064] | 14,910.0 | .46 | >.99 | No |
| Claude 4.5 (FRES Prompt) | Gemini 3 (Baseline) | 2.735 [2.692, 2.778] | 2.723 [2.678, 2.767] | 0.013 [-0.034, 0.059] | 14,952.0 | .60 | >.99 | No |
| Claude 4.5 (Semantic) | Gemini 3 (FRES Prompt) | 2.600 [2.552, 2.648] | 2.647 [2.600, 2.695] | -0.048 [-0.101, 0.006] | 17,050.0 | .08 | >.99 | No |
| Claude 4.5 (Semantic) | Gemini 3 (Semantic) | 2.600 [2.552, 2.648] | 2.603 [2.554, 2.651] | -0.003 [-0.057, 0.052] | 20,679.0 | .93 | >.99 | No |
| Claude 4.5 (Semantic) | Gemini 3 (Textstat) | 2.600 [2.552, 2.648] | 2.618 [2.570, 2.665] | -0.018 [-0.072, 0.037] | 19,942.0 | .53 | >.99 | No |
| GPT 5.2 (FRES Prompt) | GPT 5.2 (Semantic) | 2.110 [2.079, 2.141] | 2.132 [2.099, 2.166] | -0.022 [-0.063, 0.018] | 10,643.0 | .28 | >.99 | No |
| GPT 5.2 (FRES Prompt) | GPT 5.2 (Textstat) | 2.110 [2.079, 2.141] | 2.125 [2.092, 2.158] | -0.015 [-0.057, 0.027] | 12,028.5 | .48 | >.99 | No |
| GPT 5.2 (FRES Prompt) | GPT 5.2 (Baseline) | 2.110 [2.079, 2.141] | 2.103 [2.073, 2.132] | 0.007 [-0.033, 0.048] | 11,744.0 | .71 | >.99 | No |
| GPT 5.2 (Semantic) | GPT 5.2 (Textstat) | 2.132 [2.099, 2.166] | 2.125 [2.092, 2.158] | 0.007 [-0.038, 0.053] | 14,678.0 | .75 | >.99 | No |
| GPT 5.2 (Semantic) | GPT 5.2 (Baseline) | 2.132 [2.099, 2.166] | 2.103 [2.073, 2.132] | 0.030 [-0.012, 0.072] | 11,268.5 | .16 | >.99 | No |
| GPT 5.2 (Textstat) | GPT 5.2 (Baseline) | 2.125 [2.092, 2.158] | 2.103 [2.073, 2.132] | 0.022 [-0.020, 0.065] | 11,979.0 | .30 | >.99 | No |
| Gemini 3 (FRES Prompt) | Gemini 3 (Semantic) | 2.647 [2.600, 2.695] | 2.603 [2.554, 2.651] | 0.045 [-0.007, 0.097] | 16,488.0 | .09 | >.99 | No |
| Gemini 3 (FRES Prompt) | Gemini 3 (Textstat) | 2.647 [2.600, 2.695] | 2.618 [2.570, 2.665] | 0.030 [-0.020, 0.080] | 16,031.0 | .24 | >.99 | No |
| Gemini 3 (Semantic) | Gemini 3 (Textstat) | 2.603 [2.554, 2.651] | 2.618 [2.570, 2.665] | -0.015 [-0.067, 0.037] | 18,258.5 | .57 | >.99 | No |
| OSS-20b (Semantic) | OSS-20b (Textstat) | 2.272 [2.229, 2.316] | 2.297 [2.253, 2.342] | -0.025 [-0.076, 0.026] | 16,680.0 | .33 | >.99 | No |

Empathy was rated on an ordinal scale from 1 to 3. Means and 95% CIs are presented as supplementary descriptive measures because medians showed a strong ceiling effect. Mean-score and paired mean-difference 95% CIs were calculated using the Student t distribution. Pairwise comparisons used 2-sided Wilcoxon signed-rank tests with the Pratt method for zero differences, with P values adjusted across all 120 comparisons using the Holm procedure. P values are reported to 2 decimal places, except when P<.01 or when rounding would alter the interpretation of statistical significance. Values below .001 are reported as P<.001, and values above .99 as P>.99. Statistical significance was defined as Holm-adjusted P<.05. Praw indicates the unadjusted P value; PHolm, the Holm-adjusted P value; and Holm sig., statistical significance after Holm correction. Abbreviation: CI, confidence interval.
