# Supplementary material for: Prompting Strategies for Readability Alignment Between User-Generated Ophthalmology Questions and Large Language Model Answers: Comparative Evaluation Study
Source: JMIR Form Res. 2026 Nov 5;10:e97408. doi: 10.2196/97408 (PMC13739205; doi:10.2196/97408)
Supplement: Supplementary file 10 [file formative-v10-e97408-s011.docx]

**Pairwise comparisons of accuracy, completeness, and empathy scores among evaluators with Holm adjustment.**

| Metric | Evaluator A | Evaluator B | Mean A (95% CI) | Mean B (95% CI) | Mean difference (95% CI) | P_raw_ | P_Holm_ | Holm sig. |
| --- | --- | --- | --- | --- | --- | --- | --- | --- |
| Accuracy | Senior ophthalmologist | Ophthalmology resident | 0.567 [0.528, 0.606] | 0.617 [0.580, 0.655] | -0.050 [-0.081, -0.019] | .002 | .008 | Yes |
| Accuracy | Senior ophthalmologist | LLM-as-a-judge | 0.567 [0.528, 0.606] | 0.539 [0.500, 0.578] | 0.028 [0.003, 0.053] | .039 | .08 | No |
| Accuracy | Ophthalmology resident | LLM-as-a-judge | 0.617 [0.580, 0.655] | 0.539 [0.500, 0.578] | 0.078 [0.052, 0.105] | <.001 | <.001 | Yes |
| Completeness | Senior ophthalmologist | Ophthalmology resident | 2.572 [2.531, 2.611] | 2.659 [2.622, 2.697] | -0.088 [-0.123, -0.052] | <.001 | <.001 | Yes |
| Completeness | Senior ophthalmologist | LLM-as-a-judge | 2.572 [2.531, 2.611] | 2.686 [2.648, 2.722] | -0.114 [-0.155, -0.073] | <.001 | <.001 | Yes |
| Completeness | Ophthalmology resident | LLM-as-a-judge | 2.659 [2.622, 2.697] | 2.686 [2.648, 2.722] | -0.027 [-0.064, 0.011] | .19 | .19 | No |
| Empathy | Senior ophthalmologist | Ophthalmology resident | 2.408 [2.369, 2.447] | 2.528 [2.489, 2.567] | -0.120 [-0.152, -0.089] | <.001 | <.001 | Yes |
| Empathy | Senior ophthalmologist | LLM-as-a-judge | 2.408 [2.369, 2.447] | 2.442 [2.403, 2.481] | -0.034 [-0.059, -0.009] | .009 | .03 | Yes |
| Empathy | Ophthalmology resident | LLM-as-a-judge | 2.528 [2.489, 2.567] | 2.442 [2.403, 2.481] | 0.086 [0.059, 0.114] | <.001 | <.001 | Yes |

Accuracy was rated from 0 to 1, whereas completeness and empathy were rated from 1 to 3. Values are means with 95% CIs calculated using 20,000 paired-bootstrap resamples. Mean differences were calculated as evaluator A minus evaluator B. Pairwise comparisons used 2-sided paired permutation tests with 100,000 permutations, with P values adjusted across all 9 comparisons using the Holm procedure. P values are shown exactly unless P<.001. Statistical significance was defined as Holm-adjusted P<.05. All comparisons used the same sample of 640 responses enriched for LLM-rated errors. Praw indicates the unadjusted P value; PHolm, the Holm-adjusted P value; and Holm sig., statistical significance after Holm correction. Abbreviations: CI, confidence interval; LLM, large language model.
