# Supplementary material for: Prompting Strategies for Readability Alignment Between User-Generated Ophthalmology Questions and Large Language Model Answers: Comparative Evaluation Study
Source: JMIR Form Res. 2026 Nov 5;10:e97408. doi: 10.2196/97408 (PMC13739205; doi:10.2196/97408)
Supplement: Supplementary file 11 [file formative-v10-e97408-s012.docx]

**Computational efficiency of the readability-alignment strategies: descriptive statistics and pairwise comparisons.**

**Table S1. Token usage, latency, and estimated cost across models and prompting strategies.**

| **Strategy** | **Model** | **Total tokens, median (IQR)** | **Max tokens** | **Latency, s, median (IQR)** | **Max, s** | **Cost per 400 questions, US $** | **Delta cost vs Baseline, %** |
| --- | --- | --- | --- | --- | --- | --- | --- |
| Textstat | GPT 5.2 | 520 (466-580) | 973 | 5.36 (4.72-5.98) | 95.07 | 1.15 | −3.5 |
|  | Gemini 3 | 1601 (1445-1751) | 3041 | 14.40 (13.16-15.53) | 27.29 | 8.01 | +7.5 |
|  | Claude 4.5 | 608 (550-670) | 1157 | 6.77 (6.31-7.31) | 10.64 | 3.21 | +2.4 |
|  | OSS-20b | 790 (719-901) | 5448 | 2.44 (0.77-4.96) | 88.49 | 0.03 | −28.6 |
| FRES Prompt | GPT 5.2 | 777 (726-842) | 1236 | 4.58 (4.22-5.06) | 9.02 | 1.32 | +10.6 |
|  | Gemini 3 | 2598 (2200-2990) | 6250 | 25.95 (20.51-32.82) | 249.98 | 12.93 | +73.7 |
|  | Claude 4.5 | 898 (851-960) | 1397 | 6.94 (6.43-7.39) | 11.27 | 4.03 | +28.5 |
|  | OSS-20b | 2296 (1728-3068) | 22,309 | 7.66 (5.22-15.41) | 461.83 | 0.14 | +186 |
| Baseline | GPT 5.2 | 487 (438-545) | 946 | 4.38 (3.97-4.80) | 189.24 | 1.19 | — |
|  | Gemini 3 | 1484 (1350-1654) | 2525 | 13.25 (12.16-14.65) | 24.03 | 7.45 | — |
|  | Claude 4.5 | 560 (508-627) | 1095 | 6.68 (6.16-7.19) | 9.55 | 3.13 | — |
|  | OSS-20b | 739 (653-916) | 8298 | 4.17 (2.32-9.44) | 138.43 | 0.05 | — |
| Semantic | GPT 5.2 | 793 (745-860) | 1289 | 5.60 (4.95-6.39) | 10.45 | 1.22 | +2.9 |
|  | Gemini 3 | 1900 (1740-2069) | 3599 | 14.08 (12.80-15.68) | 33.33 | 8.29 | +11.3 |
|  | Claude 4.5 | 932 (887-1004) | 1494 | 7.49 (6.91-8.13) | 32.16 | 4.11 | +31.1 |
|  | OSS-20b | 1107 (1020-1226) | 3069 | 0.91 (0.74-2.86) | 58.01 | 0.03 | −30.5 |

Values are medians with interquartile ranges (IQR) across 400 questions per configuration; maxima document dispersion. Cost is the total estimated application programming interface cost for the full 400-question set, computed by multiplying provider-reported per-million-token prices by observed input and output token counts. The delta column expresses cost relative to the same model’s Baseline condition. In OSS-20b, the Semantic and Textstat conditions cost less than Baseline despite longer prompts, because the Baseline condition produced a long right tail of output lengths (mean 789 vs median 405 output tokens); cost totals are computed from summed tokens rather than from medians. Latency reflects end-to-end response time and is influenced by provider-side load; it should be interpreted as indicative rather than as a benchmark of model speed. Figure 6 in the main manuscript applies a visual cap for display (5000 tokens, 50 seconds, US $0.05); untruncated values are reported here. Abbreviations: FRES, Flesch Reading Ease Score; IQR, interquartile range.

**Table S2. Pairwise comparisons of total token usage with Holm adjustment.**

| **Condition A** | **Condition B** | **Median A (IQR)** | **Median B (IQR)** | **Median difference (95% CI)** | **Wilcoxon W** | ***P*raw** | ***P*Holm** | **Holm sig.** |
| --- | --- | --- | --- | --- | --- | --- | --- | --- |
| Gemini 3 (FRES Prompt) | Claude 4.5 (Baseline) | 2598 (2200-2990) | 560 (508-627) | 2042 (1994-2092) | 0.0 | <.001 | <.001 | Yes |
| Gemini 3 (FRES Prompt) | Claude 4.5 (Textstat) | 2598 (2200-2990) | 608 (550-670) | 1999 (1951-2049) | 0.0 | <.001 | <.001 | Yes |
| Gemini 3 (FRES Prompt) | OSS-20b (Textstat) | 2598 (2200-2990) | 790 (719-901) | 1783 (1730-1836) | 1547.5 | <.001 | <.001 | Yes |
| Gemini 3 (FRES Prompt) | OSS-20b (Baseline) | 2598 (2200-2990) | 739 (653-916) | 1734 (1672-1796) | 6048.5 | <.001 | <.001 | Yes |
| Gemini 3 (FRES Prompt) | Claude 4.5 (FRES Prompt) | 2598 (2200-2990) | 898 (851-960) | 1708 (1659-1758) | 0.0 | <.001 | <.001 | Yes |
| Gemini 3 (FRES Prompt) | Claude 4.5 (Semantic) | 2598 (2200-2990) | 932 (887-1004) | 1667 (1619-1717) | 0.0 | <.001 | <.001 | Yes |
| OSS-20b (FRES Prompt) | OSS-20b (Textstat) | 2296 (1728-3068) | 790 (719-901) | 1538 (1434-1652) | 1785.5 | <.001 | <.001 | Yes |
| Gemini 3 (FRES Prompt) | OSS-20b (Semantic) | 2598 (2200-2990) | 1107 (1020-1226) | 1489 (1443-1537) | 0.0 | <.001 | <.001 | Yes |
| Gemini 3 (Semantic) | Claude 4.5 (Baseline) | 1900 (1740-2069) | 560 (508-627) | 1324 (1306-1344) | 0.0 | <.001 | <.001 | Yes |
| Gemini 3 (Semantic) | Claude 4.5 (Textstat) | 1900 (1740-2069) | 608 (550-670) | 1282 (1263-1300) | 0.0 | <.001 | <.001 | Yes |
| Gemini 3 (Semantic) | OSS-20b (Baseline) | 1900 (1740-2069) | 739 (653-916) | 1095 (1062-1126) | 8957.5 | <.001 | <.001 | Yes |
| Gemini 3 (Semantic) | OSS-20b (Textstat) | 1900 (1740-2069) | 790 (719-901) | 1086 (1064-1108) | 3699.0 | <.001 | <.001 | Yes |
| Gemini 3 (Textstat) | Claude 4.5 (Baseline) | 1601 (1445-1751) | 560 (508-627) | 1030 (1012-1048) | 0.0 | <.001 | <.001 | Yes |
| Gemini 3 (Semantic) | Claude 4.5 (FRES Prompt) | 1900 (1740-2069) | 898 (851-960) | 990 (970-1009) | 0.0 | <.001 | <.001 | Yes |
| Gemini 3 (FRES Prompt) | Gemini 3 (Textstat) | 2598 (2200-2990) | 1601 (1445-1751) | 989 (945-1033) | 14.0 | <.001 | <.001 | Yes |
| Gemini 3 (Textstat) | Claude 4.5 (Textstat) | 1601 (1445-1751) | 608 (550-670) | 987 (970-1005) | 0.0 | <.001 | <.001 | Yes |
| Gemini 3 (Semantic) | Claude 4.5 (Semantic) | 1900 (1740-2069) | 932 (887-1004) | 949 (931-968) | 0.0 | <.001 | <.001 | Yes |
| Gemini 3 (Baseline) | Claude 4.5 (Baseline) | 1484 (1350-1654) | 560 (508-627) | 922 (905-939) | 0.0 | <.001 | <.001 | Yes |
| Gemini 3 (Baseline) | Claude 4.5 (Textstat) | 1484 (1350-1654) | 608 (550-670) | 880 (862-897) | 0.0 | <.001 | <.001 | Yes |
| Gemini 3 (Textstat) | OSS-20b (Baseline) | 1601 (1445-1751) | 739 (653-916) | 808 (778-834) | 11,019.5 | <.001 | <.001 | Yes |
| Gemini 3 (Textstat) | OSS-20b (Textstat) | 1601 (1445-1751) | 790 (719-901) | 797 (776-818) | 4882.5 | <.001 | <.001 | Yes |
| Gemini 3 (Semantic) | OSS-20b (Semantic) | 1900 (1740-2069) | 1107 (1020-1226) | 778 (760-796) | 130.5 | <.001 | <.001 | Yes |
| Gemini 3 (Textstat) | Claude 4.5 (FRES Prompt) | 1601 (1445-1751) | 898 (851-960) | 696 (678-714) | 0.0 | <.001 | <.001 | Yes |
| Gemini 3 (Baseline) | OSS-20b (Baseline) | 1484 (1350-1654) | 739 (653-916) | 694 (663-722) | 12,825.0 | <.001 | <.001 | Yes |
| Gemini 3 (Baseline) | OSS-20b (Textstat) | 1484 (1350-1654) | 790 (719-901) | 684 (663-706) | 5264.5 | <.001 | <.001 | Yes |
| Gemini 3 (Textstat) | Claude 4.5 (Semantic) | 1601 (1445-1751) | 932 (887-1004) | 656 (638-674) | 0.0 | <.001 | <.001 | Yes |
| Gemini 3 (Baseline) | Claude 4.5 (FRES Prompt) | 1484 (1350-1654) | 898 (851-960) | 587 (570-605) | 0.0 | <.001 | <.001 | Yes |
| Gemini 3 (Baseline) | Claude 4.5 (Semantic) | 1484 (1350-1654) | 932 (887-1004) | 548 (530-566) | 0.0 | <.001 | <.001 | Yes |
| Gemini 3 (Textstat) | OSS-20b (Semantic) | 1601 (1445-1751) | 1107 (1020-1226) | 486 (468-505) | 735.0 | <.001 | <.001 | Yes |
| Gemini 3 (Baseline) | OSS-20b (Semantic) | 1484 (1350-1654) | 1107 (1020-1226) | 376 (358-394) | 819.0 | <.001 | <.001 | Yes |
| Claude 4.5 (Semantic) | Claude 4.5 (Textstat) | 932 (887-1004) | 608 (550-670) | 332 (330-334) | 0.0 | <.001 | <.001 | Yes |
| OSS-20b (Semantic) | OSS-20b (Textstat) | 1107 (1020-1226) | 790 (719-901) | 317 (304-330) | 6768.0 | <.001 | <.001 | Yes |
| Claude 4.5 (FRES Prompt) | Claude 4.5 (Textstat) | 898 (851-960) | 608 (550-670) | 292 (290-294) | 0.0 | <.001 | <.001 | Yes |
| Gemini 3 (Semantic) | Gemini 3 (Textstat) | 1900 (1740-2069) | 1601 (1445-1751) | 288 (268-308) | 3879.5 | <.001 | <.001 | Yes |
| GPT 5.2 (Semantic) | GPT 5.2 (Textstat) | 793 (745-860) | 520 (466-580) | 278 (276-279) | 0.0 | <.001 | <.001 | Yes |
| GPT 5.2 (FRES Prompt) | GPT 5.2 (Textstat) | 777 (726-842) | 520 (466-580) | 260 (258-262) | 0.0 | <.001 | <.001 | Yes |
| Gemini 3 (FRES Prompt) | OSS-20b (FRES Prompt) | 2598 (2200-2990) | 2296 (1728-3068) | 256 (142-355) | 30,704.5 | <.001 | <.001 | Yes |
| GPT 5.2 (Semantic) | Claude 4.5 (Baseline) | 793 (745-860) | 560 (508-627) | 233 (232-234) | 0.0 | <.001 | <.001 | Yes |
| GPT 5.2 (FRES Prompt) | Claude 4.5 (Baseline) | 777 (726-842) | 560 (508-627) | 216 (214-217) | 0.0 | <.001 | <.001 | Yes |
| Claude 4.5 (Semantic) | OSS-20b (Baseline) | 932 (887-1004) | 739 (653-916) | 191 (172-207) | 24,366.0 | <.001 | <.001 | Yes |
| GPT 5.2 (Semantic) | Claude 4.5 (Textstat) | 793 (745-860) | 608 (550-670) | 190 (188-192) | 0.0 | <.001 | <.001 | Yes |
| GPT 5.2 (FRES Prompt) | Claude 4.5 (Textstat) | 777 (726-842) | 608 (550-670) | 172 (171-174) | 0.0 | <.001 | <.001 | Yes |
| Claude 4.5 (FRES Prompt) | OSS-20b (Baseline) | 898 (851-960) | 739 (653-916) | 154 (134-169) | 25,211.0 | <.001 | <.001 | Yes |
| Claude 4.5 (Semantic) | OSS-20b (Textstat) | 932 (887-1004) | 790 (719-901) | 150 (138-160) | 8411.5 | <.001 | <.001 | Yes |
| Claude 4.5 (FRES Prompt) | OSS-20b (Textstat) | 898 (851-960) | 790 (719-901) | 110 (100-121) | 10,803.5 | <.001 | <.001 | Yes |
| GPT 5.2 (Semantic) | OSS-20b (Baseline) | 793 (745-860) | 739 (653-916) | 50 (32-66) | 30,771.0 | <.001 | <.001 | Yes |
| Claude 4.5 (Semantic) | Claude 4.5 (FRES Prompt) | 932 (887-1004) | 898 (851-960) | 39 (37-42) | 214.5 | <.001 | <.001 | Yes |
| GPT 5.2 (FRES Prompt) | OSS-20b (Baseline) | 777 (726-842) | 739 (653-916) | 33 (14-49) | 33,105.0 | .003 | .008 | Yes |
| GPT 5.2 (Semantic) | GPT 5.2 (FRES Prompt) | 793 (745-860) | 777 (726-842) | 18 (16-19) | 2553.0 | <.001 | <.001 | Yes |
| GPT 5.2 (Semantic) | OSS-20b (Textstat) | 793 (745-860) | 790 (719-901) | 10 (−1 to 20) | 36,030.0 | .08 | .16 | No |
| GPT 5.2 (FRES Prompt) | OSS-20b (Textstat) | 777 (726-842) | 790 (719-901) | −8 (−20 to 2) | 36,293.5 | .10 | .16 | No |
| GPT 5.2 (Baseline) | GPT 5.2 (Textstat) | 487 (438-545) | 520 (466-580) | −31 (−32 to −30) | 102.0 | <.001 | <.001 | Yes |
| OSS-20b (Baseline) | OSS-20b (Textstat) | 739 (653-916) | 790 (719-901) | −32 (−48 to −13) | 32,986.0 | .002 | .008 | Yes |
| Claude 4.5 (Baseline) | Claude 4.5 (Textstat) | 560 (508-627) | 608 (550-670) | −42 (−44 to −41) | 1.0 | <.001 | <.001 | Yes |
| GPT 5.2 (Textstat) | Claude 4.5 (Baseline) | 520 (466-580) | 560 (508-627) | −45 (−46 to −43) | 8.5 | <.001 | <.001 | Yes |
| GPT 5.2 (Baseline) | Claude 4.5 (Baseline) | 487 (438-545) | 560 (508-627) | −76 (−78 to −74) | 0.0 | <.001 | <.001 | Yes |
| GPT 5.2 (Textstat) | Claude 4.5 (Textstat) | 520 (466-580) | 608 (550-670) | −87 (−89 to −86) | 0.0 | <.001 | <.001 | Yes |
| GPT 5.2 (Semantic) | Claude 4.5 (FRES Prompt) | 793 (745-860) | 898 (851-960) | −102 (−104 to −100) | 0.0 | <.001 | <.001 | Yes |
| Gemini 3 (Baseline) | Gemini 3 (Textstat) | 1484 (1350-1654) | 1601 (1445-1751) | −117 (−132 to −102) | 11,844.5 | <.001 | <.001 | Yes |
| GPT 5.2 (Baseline) | Claude 4.5 (Textstat) | 487 (438-545) | 608 (550-670) | −118 (−120 to −116) | 0.0 | <.001 | <.001 | Yes |
| GPT 5.2 (FRES Prompt) | Claude 4.5 (FRES Prompt) | 777 (726-842) | 898 (851-960) | −120 (−122 to −118) | 0.0 | <.001 | <.001 | Yes |
| Claude 4.5 (Textstat) | OSS-20b (Baseline) | 608 (550-670) | 739 (653-916) | −139 (−159 to −123) | 1196.5 | <.001 | <.001 | Yes |
| GPT 5.2 (Semantic) | Claude 4.5 (Semantic) | 793 (745-860) | 932 (887-1004) | −142 (−144 to −139) | 0.0 | <.001 | <.001 | Yes |
| GPT 5.2 (FRES Prompt) | Claude 4.5 (Semantic) | 777 (726-842) | 932 (887-1004) | −159 (−162 to −156) | 0.0 | <.001 | <.001 | Yes |
| Claude 4.5 (Semantic) | OSS-20b (Semantic) | 932 (887-1004) | 1107 (1020-1226) | −168 (−178 to −158) | 57.5 | <.001 | <.001 | Yes |
| Claude 4.5 (Baseline) | OSS-20b (Baseline) | 560 (508-627) | 739 (653-916) | −182 (−202 to −166) | 293.0 | <.001 | <.001 | Yes |
| Claude 4.5 (Textstat) | OSS-20b (Textstat) | 608 (550-670) | 790 (719-901) | −182 (−192 to −171) | 0.0 | <.001 | <.001 | Yes |
| Claude 4.5 (FRES Prompt) | OSS-20b (Semantic) | 898 (851-960) | 1107 (1020-1226) | −207 (−216 to −197) | 0.0 | <.001 | <.001 | Yes |
| Claude 4.5 (Baseline) | OSS-20b (Textstat) | 560 (508-627) | 790 (719-901) | −224 (−235 to −214) | 0.0 | <.001 | <.001 | Yes |
| GPT 5.2 (Textstat) | OSS-20b (Baseline) | 520 (466-580) | 739 (653-916) | −227 (−246 to −211) | 106.5 | <.001 | <.001 | Yes |
| GPT 5.2 (Baseline) | OSS-20b (Baseline) | 487 (438-545) | 739 (653-916) | −258 (−278 to −242) | 11.5 | <.001 | <.001 | Yes |
| GPT 5.2 (Textstat) | OSS-20b (Textstat) | 520 (466-580) | 790 (719-901) | −269 (−280 to −258) | 0.0 | <.001 | <.001 | Yes |
| GPT 5.2 (Baseline) | GPT 5.2 (FRES Prompt) | 487 (438-545) | 777 (726-842) | −291 (−292 to −290) | 0.0 | <.001 | <.001 | Yes |
| GPT 5.2 (Baseline) | OSS-20b (Textstat) | 487 (438-545) | 790 (719-901) | −300 (−310 to −289) | 0.0 | <.001 | <.001 | Yes |
| GPT 5.2 (Baseline) | GPT 5.2 (Semantic) | 487 (438-545) | 793 (745-860) | −309 (−310 to −308) | 0.0 | <.001 | <.001 | Yes |
| GPT 5.2 (Semantic) | OSS-20b (Semantic) | 793 (745-860) | 1107 (1020-1226) | −309 (−319 to −299) | 0.0 | <.001 | <.001 | Yes |
| GPT 5.2 (FRES Prompt) | OSS-20b (Semantic) | 777 (726-842) | 1107 (1020-1226) | −326 (−336 to −317) | 0.0 | <.001 | <.001 | Yes |
| Claude 4.5 (Baseline) | Claude 4.5 (FRES Prompt) | 560 (508-627) | 898 (851-960) | −334 (−336 to −332) | 0.0 | <.001 | <.001 | Yes |
| OSS-20b (Baseline) | OSS-20b (Semantic) | 739 (653-916) | 1107 (1020-1226) | −346 (−363 to −324) | 19,930.0 | <.001 | <.001 | Yes |
| Claude 4.5 (Baseline) | Claude 4.5 (Semantic) | 560 (508-627) | 932 (887-1004) | −374 (−376 to −372) | 0.0 | <.001 | <.001 | Yes |
| GPT 5.2 (Textstat) | Claude 4.5 (FRES Prompt) | 520 (466-580) | 898 (851-960) | −379 (−382 to −377) | 0.0 | <.001 | <.001 | Yes |
| Gemini 3 (Baseline) | Gemini 3 (Semantic) | 1484 (1350-1654) | 1900 (1740-2069) | −404 (−422 to −386) | 63.5 | <.001 | <.001 | Yes |
| GPT 5.2 (Baseline) | Claude 4.5 (FRES Prompt) | 487 (438-545) | 898 (851-960) | −410 (−413 to −408) | 0.0 | <.001 | <.001 | Yes |
| GPT 5.2 (Textstat) | Claude 4.5 (Semantic) | 520 (466-580) | 932 (887-1004) | −420 (−422 to −417) | 0.0 | <.001 | <.001 | Yes |
| GPT 5.2 (Baseline) | Claude 4.5 (Semantic) | 487 (438-545) | 932 (887-1004) | −450 (−453 to −448) | 0.0 | <.001 | <.001 | Yes |
| Gemini 3 (Semantic) | OSS-20b (FRES Prompt) | 1900 (1740-2069) | 2296 (1728-3068) | −466 (−590 to −358) | 16,998.5 | <.001 | <.001 | Yes |
| Claude 4.5 (Textstat) | OSS-20b (Semantic) | 608 (550-670) | 1107 (1020-1226) | −500 (−510 to −490) | 0.0 | <.001 | <.001 | Yes |
| Claude 4.5 (Baseline) | OSS-20b (Semantic) | 560 (508-627) | 1107 (1020-1226) | −542 (−552 to −532) | 0.0 | <.001 | <.001 | Yes |
| GPT 5.2 (Textstat) | OSS-20b (Semantic) | 520 (466-580) | 1107 (1020-1226) | −586 (−597 to −577) | 0.0 | <.001 | <.001 | Yes |
| GPT 5.2 (Baseline) | OSS-20b (Semantic) | 487 (438-545) | 1107 (1020-1226) | −618 (−628 to −608) | 0.0 | <.001 | <.001 | Yes |
| GPT 5.2 (Semantic) | Gemini 3 (Baseline) | 793 (745-860) | 1484 (1350-1654) | −689 (−706 to −672) | 0.0 | <.001 | <.001 | Yes |
| Gemini 3 (Semantic) | Gemini 3 (FRES Prompt) | 1900 (1740-2069) | 2598 (2200-2990) | −700 (−744 to −657) | 911.0 | <.001 | <.001 | Yes |
| GPT 5.2 (FRES Prompt) | Gemini 3 (Baseline) | 777 (726-842) | 1484 (1350-1654) | −706 (−724 to −689) | 0.0 | <.001 | <.001 | Yes |
| Gemini 3 (Textstat) | OSS-20b (FRES Prompt) | 1601 (1445-1751) | 2296 (1728-3068) | −762 (−882 to −656) | 4230.5 | <.001 | <.001 | Yes |
| GPT 5.2 (Semantic) | Gemini 3 (Textstat) | 793 (745-860) | 1601 (1445-1751) | −798 (−816 to −780) | 0.0 | <.001 | <.001 | Yes |
| GPT 5.2 (FRES Prompt) | Gemini 3 (Textstat) | 777 (726-842) | 1601 (1445-1751) | −814 (−832 to −797) | 0.0 | <.001 | <.001 | Yes |
| Gemini 3 (Baseline) | OSS-20b (FRES Prompt) | 1484 (1350-1654) | 2296 (1728-3068) | −886 (−998 to −782) | 1100.5 | <.001 | <.001 | Yes |
| GPT 5.2 (Textstat) | Gemini 3 (Baseline) | 520 (466-580) | 1484 (1350-1654) | −966 (−984 to −949) | 0.0 | <.001 | <.001 | Yes |
| GPT 5.2 (Baseline) | Gemini 3 (Baseline) | 487 (438-545) | 1484 (1350-1654) | −998 (−1015 to −980) | 0.0 | <.001 | <.001 | Yes |
| GPT 5.2 (Textstat) | Gemini 3 (Textstat) | 520 (466-580) | 1601 (1445-1751) | −1075 (−1093 to −1058) | 0.0 | <.001 | <.001 | Yes |
| GPT 5.2 (Semantic) | Gemini 3 (Semantic) | 793 (745-860) | 1900 (1740-2069) | −1092 (−1110 to −1072) | 0.0 | <.001 | <.001 | Yes |
| GPT 5.2 (Baseline) | Gemini 3 (Textstat) | 487 (438-545) | 1601 (1445-1751) | −1106 (−1124 to −1088) | 0.0 | <.001 | <.001 | Yes |
| GPT 5.2 (FRES Prompt) | Gemini 3 (Semantic) | 777 (726-842) | 1900 (1740-2069) | −1108 (−1128 to −1090) | 0.0 | <.001 | <.001 | Yes |
| Gemini 3 (Baseline) | Gemini 3 (FRES Prompt) | 1484 (1350-1654) | 2598 (2200-2990) | −1112 (−1155 to −1071) | 0.0 | <.001 | <.001 | Yes |
| OSS-20b (Semantic) | OSS-20b (FRES Prompt) | 1107 (1020-1226) | 2296 (1728-3068) | −1268 (−1382 to −1164) | 47.0 | <.001 | <.001 | Yes |
| GPT 5.2 (Textstat) | Gemini 3 (Semantic) | 520 (466-580) | 1900 (1740-2069) | −1369 (−1388 to −1350) | 0.0 | <.001 | <.001 | Yes |
| GPT 5.2 (Baseline) | Gemini 3 (Semantic) | 487 (438-545) | 1900 (1740-2069) | −1400 (−1420 to −1382) | 0.0 | <.001 | <.001 | Yes |
| Claude 4.5 (Semantic) | OSS-20b (FRES Prompt) | 932 (887-1004) | 2296 (1728-3068) | −1445 (−1556 to −1344) | 0.0 | <.001 | <.001 | Yes |
| OSS-20b (Baseline) | OSS-20b (FRES Prompt) | 739 (653-916) | 2296 (1728-3068) | −1460 (−1571 to −1356) | 5582.0 | <.001 | <.001 | Yes |
| Claude 4.5 (FRES Prompt) | OSS-20b (FRES Prompt) | 898 (851-960) | 2296 (1728-3068) | −1485 (−1597 to −1384) | 0.0 | <.001 | <.001 | Yes |
| GPT 5.2 (Semantic) | OSS-20b (FRES Prompt) | 793 (745-860) | 2296 (1728-3068) | −1586 (−1698 to −1484) | 0.0 | <.001 | <.001 | Yes |
| GPT 5.2 (FRES Prompt) | OSS-20b (FRES Prompt) | 777 (726-842) | 2296 (1728-3068) | −1604 (−1716 to −1503) | 0.0 | <.001 | <.001 | Yes |
| Claude 4.5 (Textstat) | OSS-20b (FRES Prompt) | 608 (550-670) | 2296 (1728-3068) | −1776 (−1888 to −1675) | 0.0 | <.001 | <.001 | Yes |
| GPT 5.2 (Semantic) | Gemini 3 (FRES Prompt) | 793 (745-860) | 2598 (2200-2990) | −1809 (−1860 to −1761) | 0.0 | <.001 | <.001 | Yes |
| Claude 4.5 (Baseline) | OSS-20b (FRES Prompt) | 560 (508-627) | 2296 (1728-3068) | −1819 (−1931 to −1718) | 0.0 | <.001 | <.001 | Yes |
| GPT 5.2 (FRES Prompt) | Gemini 3 (FRES Prompt) | 777 (726-842) | 2598 (2200-2990) | −1827 (−1876 to −1778) | 0.0 | <.001 | <.001 | Yes |
| GPT 5.2 (Textstat) | OSS-20b (FRES Prompt) | 520 (466-580) | 2296 (1728-3068) | −1865 (−1976 to −1763) | 0.0 | <.001 | <.001 | Yes |
| GPT 5.2 (Baseline) | OSS-20b (FRES Prompt) | 487 (438-545) | 2296 (1728-3068) | −1896 (−2007 to −1794) | 0.0 | <.001 | <.001 | Yes |
| GPT 5.2 (Textstat) | Gemini 3 (FRES Prompt) | 520 (466-580) | 2598 (2200-2990) | −2087 (−2137 to −2038) | 0.0 | <.001 | <.001 | Yes |
| GPT 5.2 (Baseline) | Gemini 3 (FRES Prompt) | 487 (438-545) | 2598 (2200-2990) | −2118 (−2169 to −2070) | 0.0 | <.001 | <.001 | Yes |

Total tokens are the sum of input and output tokens per response (n=400 paired observations per comparison). Paired differences are Hodges-Lehmann estimates with exact distribution-free 95% CIs. Pairwise comparisons used 2-sided Wilcoxon signed-rank tests with the Pratt method for zero differences, with P values adjusted across all 120 comparisons using the Holm procedure. P values are reported to 2 decimal places, except when P<.01 or when rounding would alter the interpretation of statistical significance. Values below .001 are reported as P<.001, and values above .99 as P>.99. Statistical significance was defined as Holm-adjusted P<.05. Praw indicates the unadjusted P value; PHolm, the Holm-adjusted P value; and Holm sig., statistical significance after Holm correction. Rows are ordered by descending median difference. Abbreviations: CI, confidence interval; FRES, Flesch Reading Ease Score; IQR, interquartile range.

**Table S3. Pairwise comparisons of latency with Holm adjustment.**

| **Condition A** | **Condition B** | **Median A, s (IQR)** | **Median B, s (IQR)** | **Median difference, s (95% CI)** | **Wilcoxon W** | ***P*raw** | ***P*Holm** | **Holm sig.** |
| --- | --- | --- | --- | --- | --- | --- | --- | --- |
| Gemini 3 (FRES Prompt) | OSS-20b (Semantic) | 25.95 (20.51-32.82) | 0.91 (0.74-2.86) | 25.57 (24.45-26.80) | 339.0 | <.001 | <.001 | Yes |
| Gemini 3 (FRES Prompt) | OSS-20b (Textstat) | 25.95 (20.51-32.82) | 2.44 (0.77-4.96) | 24.18 (22.82-25.57) | 932.0 | <.001 | <.001 | Yes |
| Gemini 3 (FRES Prompt) | OSS-20b (Baseline) | 25.95 (20.51-32.82) | 4.17 (2.32-9.44) | 21.02 (19.55-22.48) | 4606.0 | <.001 | <.001 | Yes |
| Gemini 3 (FRES Prompt) | Claude 4.5 (Baseline) | 25.95 (20.51-32.82) | 6.68 (6.16-7.19) | 20.11 (18.99-21.31) | 0.0 | <.001 | <.001 | Yes |
| Gemini 3 (FRES Prompt) | Claude 4.5 (Textstat) | 25.95 (20.51-32.82) | 6.77 (6.31-7.31) | 20.00 (18.89-21.21) | 0.0 | <.001 | <.001 | Yes |
| Gemini 3 (FRES Prompt) | Claude 4.5 (FRES Prompt) | 25.95 (20.51-32.82) | 6.94 (6.43-7.39) | 19.94 (18.85-21.12) | 0.0 | <.001 | <.001 | Yes |
| Gemini 3 (FRES Prompt) | Claude 4.5 (Semantic) | 25.95 (20.51-32.82) | 7.49 (6.91-8.13) | 19.23 (18.14-20.46) | 1.0 | <.001 | <.001 | Yes |
| Gemini 3 (FRES Prompt) | OSS-20b (FRES Prompt) | 25.95 (20.51-32.82) | 7.66 (5.22-15.41) | 17.68 (15.86-19.27) | 14,524.0 | <.001 | <.001 | Yes |
| Gemini 3 (Textstat) | OSS-20b (Semantic) | 14.40 (13.16-15.53) | 0.91 (0.74-2.86) | 12.71 (12.38-13.04) | 403.0 | <.001 | <.001 | Yes |
| Gemini 3 (Semantic) | OSS-20b (Semantic) | 14.08 (12.80-15.68) | 0.91 (0.74-2.86) | 12.40 (12.06-12.73) | 402.0 | <.001 | <.001 | Yes |
| Gemini 3 (FRES Prompt) | Gemini 3 (Textstat) | 25.95 (20.51-32.82) | 14.40 (13.16-15.53) | 12.05 (10.99-13.24) | 327.5 | <.001 | <.001 | Yes |
| Gemini 3 (Baseline) | OSS-20b (Semantic) | 13.25 (12.16-14.65) | 0.91 (0.74-2.86) | 11.66 (11.36-11.94) | 409.0 | <.001 | <.001 | Yes |
| Gemini 3 (Textstat) | OSS-20b (Textstat) | 14.40 (13.16-15.53) | 2.44 (0.77-4.96) | 11.12 (10.57-11.62) | 2566.5 | <.001 | <.001 | Yes |
| Gemini 3 (Semantic) | OSS-20b (Textstat) | 14.08 (12.80-15.68) | 2.44 (0.77-4.96) | 10.82 (10.23-11.34) | 2843.0 | <.001 | <.001 | Yes |
| Gemini 3 (Baseline) | OSS-20b (Textstat) | 13.25 (12.16-14.65) | 2.44 (0.77-4.96) | 10.06 (9.50-10.55) | 3592.0 | <.001 | <.001 | Yes |
| Gemini 3 (Textstat) | OSS-20b (Baseline) | 14.40 (13.16-15.53) | 4.17 (2.32-9.44) | 8.78 (8.02-9.43) | 11,619.0 | <.001 | <.001 | Yes |
| Gemini 3 (Semantic) | OSS-20b (Baseline) | 14.08 (12.80-15.68) | 4.17 (2.32-9.44) | 8.37 (7.64-9.02) | 11,861.0 | <.001 | <.001 | Yes |
| Gemini 3 (Textstat) | Claude 4.5 (Baseline) | 14.40 (13.16-15.53) | 6.68 (6.16-7.19) | 7.69 (7.51-7.88) | 0.0 | <.001 | <.001 | Yes |
| Gemini 3 (Baseline) | OSS-20b (Baseline) | 13.25 (12.16-14.65) | 4.17 (2.32-9.44) | 7.66 (6.90-8.33) | 12,958.5 | <.001 | <.001 | Yes |
| Gemini 3 (Textstat) | Claude 4.5 (Textstat) | 14.40 (13.16-15.53) | 6.77 (6.31-7.31) | 7.61 (7.42-7.79) | 0.0 | <.001 | <.001 | Yes |
| Gemini 3 (Textstat) | Claude 4.5 (FRES Prompt) | 14.40 (13.16-15.53) | 6.94 (6.43-7.39) | 7.48 (7.29-7.67) | 0.0 | <.001 | <.001 | Yes |
| Gemini 3 (Semantic) | Claude 4.5 (Baseline) | 14.08 (12.80-15.68) | 6.68 (6.16-7.19) | 7.44 (7.24-7.65) | 0.0 | <.001 | <.001 | Yes |
| Gemini 3 (Semantic) | Claude 4.5 (Textstat) | 14.08 (12.80-15.68) | 6.77 (6.31-7.31) | 7.33 (7.12-7.54) | 0.0 | <.001 | <.001 | Yes |
| Gemini 3 (Semantic) | Claude 4.5 (FRES Prompt) | 14.08 (12.80-15.68) | 6.94 (6.43-7.39) | 7.19 (6.98-7.41) | 0.0 | <.001 | <.001 | Yes |
| Gemini 3 (Textstat) | Claude 4.5 (Semantic) | 14.40 (13.16-15.53) | 7.49 (6.91-8.13) | 6.89 (6.70-7.09) | 785.0 | <.001 | <.001 | Yes |
| Gemini 3 (Baseline) | Claude 4.5 (Baseline) | 13.25 (12.16-14.65) | 6.68 (6.16-7.19) | 6.66 (6.49-6.84) | 0.0 | <.001 | <.001 | Yes |
| Gemini 3 (Baseline) | Claude 4.5 (Textstat) | 13.25 (12.16-14.65) | 6.77 (6.31-7.31) | 6.58 (6.40-6.76) | 0.0 | <.001 | <.001 | Yes |
| Gemini 3 (Semantic) | Claude 4.5 (Semantic) | 14.08 (12.80-15.68) | 7.49 (6.91-8.13) | 6.57 (6.37-6.79) | 789.0 | <.001 | <.001 | Yes |
| Gemini 3 (Baseline) | Claude 4.5 (FRES Prompt) | 13.25 (12.16-14.65) | 6.94 (6.43-7.39) | 6.45 (6.27-6.63) | 0.0 | <.001 | <.001 | Yes |
| OSS-20b (FRES Prompt) | OSS-20b (Textstat) | 7.66 (5.22-15.41) | 2.44 (0.77-4.96) | 6.45 (5.72-7.32) | 5657.5 | <.001 | <.001 | Yes |
| Gemini 3 (Baseline) | Claude 4.5 (Semantic) | 13.25 (12.16-14.65) | 7.49 (6.91-8.13) | 5.88 (5.69-6.07) | 800.0 | <.001 | <.001 | Yes |
| Claude 4.5 (Semantic) | OSS-20b (Semantic) | 7.49 (6.91-8.13) | 0.91 (0.74-2.86) | 5.84 (5.60-6.07) | 1205.0 | <.001 | <.001 | Yes |
| Claude 4.5 (FRES Prompt) | OSS-20b (Semantic) | 6.94 (6.43-7.39) | 0.91 (0.74-2.86) | 5.27 (5.04-5.48) | 1499.0 | <.001 | <.001 | Yes |
| Claude 4.5 (Textstat) | OSS-20b (Semantic) | 6.77 (6.31-7.31) | 0.91 (0.74-2.86) | 5.11 (4.88-5.34) | 1568.0 | <.001 | <.001 | Yes |
| Claude 4.5 (Baseline) | OSS-20b (Semantic) | 6.68 (6.16-7.19) | 0.91 (0.74-2.86) | 5.02 (4.79-5.25) | 1653.0 | <.001 | <.001 | Yes |
| Gemini 3 (Textstat) | OSS-20b (FRES Prompt) | 14.40 (13.16-15.53) | 7.66 (5.22-15.41) | 4.66 (3.22-5.83) | 28,509.0 | <.001 | <.001 | Yes |
| Claude 4.5 (Semantic) | OSS-20b (Textstat) | 7.49 (6.91-8.13) | 2.44 (0.77-4.96) | 4.44 (3.90-4.86) | 15,856.5 | <.001 | <.001 | Yes |
| Gemini 3 (Semantic) | OSS-20b (FRES Prompt) | 14.08 (12.80-15.68) | 7.66 (5.22-15.41) | 4.43 (3.05-5.56) | 28,562.5 | <.001 | <.001 | Yes |
| GPT 5.2 (Semantic) | OSS-20b (Semantic) | 5.60 (4.95-6.39) | 0.91 (0.74-2.86) | 3.94 (3.69-4.21) | 4084.0 | <.001 | <.001 | Yes |
| Claude 4.5 (FRES Prompt) | OSS-20b (Textstat) | 6.94 (6.43-7.39) | 2.44 (0.77-4.96) | 3.84 (3.31-4.25) | 17,885.5 | <.001 | <.001 | Yes |
| GPT 5.2 (Textstat) | OSS-20b (Semantic) | 5.36 (4.72-5.98) | 0.91 (0.74-2.86) | 3.78 (3.51-4.03) | 4597.5 | <.001 | <.001 | Yes |
| Claude 4.5 (Textstat) | OSS-20b (Textstat) | 6.77 (6.31-7.31) | 2.44 (0.77-4.96) | 3.71 (3.15-4.12) | 19,106.0 | <.001 | <.001 | Yes |
| Gemini 3 (Baseline) | OSS-20b (FRES Prompt) | 13.25 (12.16-14.65) | 7.66 (5.22-15.41) | 3.64 (2.21-4.74) | 30,752.5 | <.001 | <.001 | Yes |
| Claude 4.5 (Baseline) | OSS-20b (Textstat) | 6.68 (6.16-7.19) | 2.44 (0.77-4.96) | 3.62 (3.07-4.05) | 19,828.5 | <.001 | <.001 | Yes |
| OSS-20b (Baseline) | OSS-20b (Semantic) | 4.17 (2.32-9.44) | 0.91 (0.74-2.86) | 3.52 (2.92-4.25) | 5580.0 | <.001 | <.001 | Yes |
| GPT 5.2 (FRES Prompt) | OSS-20b (Semantic) | 4.58 (4.22-5.06) | 0.91 (0.74-2.86) | 3.01 (2.78-3.25) | 5036.5 | <.001 | <.001 | Yes |
| GPT 5.2 (Baseline) | OSS-20b (Semantic) | 4.38 (3.97-4.80) | 0.91 (0.74-2.86) | 2.78 (2.55-3.00) | 5843.5 | <.001 | <.001 | Yes |
| GPT 5.2 (Semantic) | OSS-20b (Textstat) | 5.60 (4.95-6.39) | 2.44 (0.77-4.96) | 2.45 (1.84-2.95) | 26,126.5 | <.001 | <.001 | Yes |
| GPT 5.2 (Textstat) | OSS-20b (Textstat) | 5.36 (4.72-5.98) | 2.44 (0.77-4.96) | 2.42 (1.80-2.88) | 26,926.0 | <.001 | <.001 | Yes |
| Claude 4.5 (Semantic) | OSS-20b (Baseline) | 7.49 (6.91-8.13) | 4.17 (2.32-9.44) | 1.94 (1.10-2.65) | 29,799.5 | <.001 | <.001 | Yes |
| OSS-20b (Baseline) | OSS-20b (Textstat) | 4.17 (2.32-9.44) | 2.44 (0.77-4.96) | 1.92 (1.39-2.56) | 22,470.5 | <.001 | <.001 | Yes |
| GPT 5.2 (FRES Prompt) | OSS-20b (Textstat) | 4.58 (4.22-5.06) | 2.44 (0.77-4.96) | 1.60 (1.06-2.04) | 29,893.5 | <.001 | <.001 | Yes |
| GPT 5.2 (Baseline) | OSS-20b (Textstat) | 4.38 (3.97-4.80) | 2.44 (0.77-4.96) | 1.40 (0.89-1.77) | 30,047.5 | <.001 | <.001 | Yes |
| Claude 4.5 (FRES Prompt) | OSS-20b (Baseline) | 6.94 (6.43-7.39) | 4.17 (2.32-9.44) | 1.33 (0.48-2.07) | 33,095.5 | .002 | .02 | Yes |
| Claude 4.5 (Textstat) | OSS-20b (Baseline) | 6.77 (6.31-7.31) | 4.17 (2.32-9.44) | 1.17 (0.33-1.85) | 33,877.0 | .007 | .04 | Yes |
| Claude 4.5 (Baseline) | OSS-20b (Baseline) | 6.68 (6.16-7.19) | 4.17 (2.32-9.44) | 1.09 (0.26-1.77) | 34,152.0 | .01 | .04 | Yes |
| GPT 5.2 (Semantic) | GPT 5.2 (FRES Prompt) | 5.60 (4.95-6.39) | 4.58 (4.22-5.06) | 0.98 (0.87-1.08) | 7573.0 | <.001 | <.001 | Yes |
| Claude 4.5 (Semantic) | Claude 4.5 (Textstat) | 7.49 (6.91-8.13) | 6.77 (6.31-7.31) | 0.71 (0.62-0.80) | 9690.0 | <.001 | <.001 | Yes |
| Claude 4.5 (Semantic) | Claude 4.5 (FRES Prompt) | 7.49 (6.91-8.13) | 6.94 (6.43-7.39) | 0.56 (0.48-0.64) | 12,165.5 | <.001 | <.001 | Yes |
| GPT 5.2 (Semantic) | GPT 5.2 (Textstat) | 5.60 (4.95-6.39) | 5.36 (4.72-5.98) | 0.29 (0.19-0.40) | 27,911.5 | <.001 | <.001 | Yes |
| Claude 4.5 (FRES Prompt) | Claude 4.5 (Textstat) | 6.94 (6.43-7.39) | 6.77 (6.31-7.31) | 0.14 (0.06-0.22) | 31,599.5 | <.001 | .002 | Yes |
| GPT 5.2 (Semantic) | OSS-20b (Baseline) | 5.60 (4.95-6.39) | 4.17 (2.32-9.44) | −0.05 (−0.87 to 0.62) | 39,752.5 | .88 | >.99 | No |
| Claude 4.5 (Baseline) | Claude 4.5 (Textstat) | 6.68 (6.16-7.19) | 6.77 (6.31-7.31) | −0.08 (−0.15 to −0.01) | 34,935.5 | .03 | .08 | No |
| GPT 5.2 (Textstat) | OSS-20b (Baseline) | 5.36 (4.72-5.98) | 4.17 (2.32-9.44) | −0.09 (−0.92 to 0.58) | 39,536.0 | .81 | >.99 | No |
| Claude 4.5 (Baseline) | Claude 4.5 (FRES Prompt) | 6.68 (6.16-7.19) | 6.94 (6.43-7.39) | −0.23 (−0.30 to −0.15) | 27,289.0 | <.001 | <.001 | Yes |
| GPT 5.2 (Baseline) | GPT 5.2 (FRES Prompt) | 4.38 (3.97-4.80) | 4.58 (4.22-5.06) | −0.24 (−0.32 to −0.17) | 26,702.0 | <.001 | <.001 | Yes |
| Gemini 3 (Semantic) | Gemini 3 (Textstat) | 14.08 (12.80-15.68) | 14.40 (13.16-15.53) | −0.32 (−0.53 to −0.09) | 33,678.0 | .006 | .04 | Yes |
| GPT 5.2 (FRES Prompt) | GPT 5.2 (Textstat) | 4.58 (4.22-5.06) | 5.36 (4.72-5.98) | −0.66 (−0.76 to −0.56) | 13,390.5 | <.001 | <.001 | Yes |
| Gemini 3 (Baseline) | Gemini 3 (Semantic) | 13.25 (12.16-14.65) | 14.08 (12.80-15.68) | −0.79 (−1.00 to −0.58) | 23,702.5 | <.001 | <.001 | Yes |
| Claude 4.5 (Baseline) | Claude 4.5 (Semantic) | 6.68 (6.16-7.19) | 7.49 (6.91-8.13) | −0.79 (−0.88 to −0.71) | 6812.0 | <.001 | <.001 | Yes |
| GPT 5.2 (Baseline) | GPT 5.2 (Textstat) | 4.38 (3.97-4.80) | 5.36 (4.72-5.98) | −0.94 (−1.05 to −0.83) | 10,610.0 | <.001 | <.001 | Yes |
| GPT 5.2 (FRES Prompt) | OSS-20b (Baseline) | 4.58 (4.22-5.06) | 4.17 (2.32-9.44) | −0.94 (−1.77 to −0.25) | 33,750.5 | .006 | .04 | Yes |
| GPT 5.2 (Semantic) | Claude 4.5 (Baseline) | 5.60 (4.95-6.39) | 6.68 (6.16-7.19) | −1.08 (−1.18 to −0.99) | 5171.0 | <.001 | <.001 | Yes |
| Gemini 3 (Baseline) | Gemini 3 (Textstat) | 13.25 (12.16-14.65) | 14.40 (13.16-15.53) | −1.09 (−1.26 to −0.92) | 14,338.5 | <.001 | <.001 | Yes |
| GPT 5.2 (Baseline) | OSS-20b (Baseline) | 4.38 (3.97-4.80) | 4.17 (2.32-9.44) | −1.12 (−1.94 to −0.44) | 32,261.5 | <.001 | .006 | Yes |
| GPT 5.2 (Semantic) | Claude 4.5 (Textstat) | 5.60 (4.95-6.39) | 6.77 (6.31-7.31) | −1.18 (−1.28 to −1.08) | 4981.5 | <.001 | <.001 | Yes |
| GPT 5.2 (Baseline) | GPT 5.2 (Semantic) | 4.38 (3.97-4.80) | 5.60 (4.95-6.39) | −1.22 (−1.33 to −1.10) | 6044.0 | <.001 | <.001 | Yes |
| OSS-20b (Semantic) | OSS-20b (Textstat) | 0.91 (0.74-2.86) | 2.44 (0.77-4.96) | −1.23 (−1.62 to −0.92) | 19,245.0 | <.001 | <.001 | Yes |
| GPT 5.2 (Semantic) | Claude 4.5 (FRES Prompt) | 5.60 (4.95-6.39) | 6.94 (6.43-7.39) | −1.32 (−1.42 to −1.21) | 4122.5 | <.001 | <.001 | Yes |
| GPT 5.2 (Textstat) | Claude 4.5 (Baseline) | 5.36 (4.72-5.98) | 6.68 (6.16-7.19) | −1.42 (−1.52 to −1.31) | 5704.0 | <.001 | <.001 | Yes |
| GPT 5.2 (Textstat) | Claude 4.5 (Textstat) | 5.36 (4.72-5.98) | 6.77 (6.31-7.31) | −1.49 (−1.60 to −1.39) | 4796.5 | <.001 | <.001 | Yes |
| GPT 5.2 (Textstat) | Claude 4.5 (FRES Prompt) | 5.36 (4.72-5.98) | 6.94 (6.43-7.39) | −1.64 (−1.75 to −1.54) | 4548.5 | <.001 | <.001 | Yes |
| GPT 5.2 (Semantic) | Claude 4.5 (Semantic) | 5.60 (4.95-6.39) | 7.49 (6.91-8.13) | −1.90 (−2.00 to −1.78) | 1035.5 | <.001 | <.001 | Yes |
| GPT 5.2 (FRES Prompt) | Claude 4.5 (Baseline) | 4.58 (4.22-5.06) | 6.68 (6.16-7.19) | −2.06 (−2.15 to −1.98) | 734.5 | <.001 | <.001 | Yes |
| Claude 4.5 (Semantic) | OSS-20b (FRES Prompt) | 7.49 (6.91-8.13) | 7.66 (5.22-15.41) | −2.12 (−3.52 to −0.98) | 30,359.0 | <.001 | <.001 | Yes |
| GPT 5.2 (FRES Prompt) | Claude 4.5 (Textstat) | 4.58 (4.22-5.06) | 6.77 (6.31-7.31) | −2.16 (−2.25 to −2.07) | 760.0 | <.001 | <.001 | Yes |
| GPT 5.2 (Textstat) | Claude 4.5 (Semantic) | 5.36 (4.72-5.98) | 7.49 (6.91-8.13) | −2.20 (−2.32 to −2.08) | 3143.0 | <.001 | <.001 | Yes |
| GPT 5.2 (FRES Prompt) | Claude 4.5 (FRES Prompt) | 4.58 (4.22-5.06) | 6.94 (6.43-7.39) | −2.29 (−2.38 to −2.21) | 373.5 | <.001 | <.001 | Yes |
| GPT 5.2 (Baseline) | Claude 4.5 (Baseline) | 4.38 (3.97-4.80) | 6.68 (6.16-7.19) | −2.31 (−2.40 to −2.23) | 948.0 | <.001 | <.001 | Yes |
| GPT 5.2 (Baseline) | Claude 4.5 (Textstat) | 4.38 (3.97-4.80) | 6.77 (6.31-7.31) | −2.40 (−2.49 to −2.31) | 935.0 | <.001 | <.001 | Yes |
| GPT 5.2 (Baseline) | Claude 4.5 (FRES Prompt) | 4.38 (3.97-4.80) | 6.94 (6.43-7.39) | −2.54 (−2.63 to −2.46) | 845.0 | <.001 | <.001 | Yes |
| Claude 4.5 (FRES Prompt) | OSS-20b (FRES Prompt) | 6.94 (6.43-7.39) | 7.66 (5.22-15.41) | −2.79 (−4.13 to −1.69) | 25,079.5 | <.001 | <.001 | Yes |
| GPT 5.2 (FRES Prompt) | Claude 4.5 (Semantic) | 4.58 (4.22-5.06) | 7.49 (6.91-8.13) | −2.86 (−2.96 to −2.76) | 188.5 | <.001 | <.001 | Yes |
| Claude 4.5 (Textstat) | OSS-20b (FRES Prompt) | 6.77 (6.31-7.31) | 7.66 (5.22-15.41) | −2.88 (−4.25 to −1.75) | 24,940.0 | <.001 | <.001 | Yes |
| Claude 4.5 (Baseline) | OSS-20b (FRES Prompt) | 6.68 (6.16-7.19) | 7.66 (5.22-15.41) | −3.02 (−4.39 to −1.83) | 24,704.5 | <.001 | <.001 | Yes |
| GPT 5.2 (Baseline) | Claude 4.5 (Semantic) | 4.38 (3.97-4.80) | 7.49 (6.91-8.13) | −3.10 (−3.21 to −3.00) | 811.0 | <.001 | <.001 | Yes |
| GPT 5.2 (Semantic) | OSS-20b (FRES Prompt) | 5.60 (4.95-6.39) | 7.66 (5.22-15.41) | −3.96 (−5.48 to −2.67) | 16,806.5 | <.001 | <.001 | Yes |
| GPT 5.2 (Textstat) | OSS-20b (FRES Prompt) | 5.36 (4.72-5.98) | 7.66 (5.22-15.41) | −4.00 (−5.53 to −2.86) | 15,042.5 | <.001 | <.001 | Yes |
| OSS-20b (Baseline) | OSS-20b (FRES Prompt) | 4.17 (2.32-9.44) | 7.66 (5.22-15.41) | −4.22 (−5.01 to −3.47) | 18,140.0 | <.001 | <.001 | Yes |
| GPT 5.2 (FRES Prompt) | OSS-20b (FRES Prompt) | 4.58 (4.22-5.06) | 7.66 (5.22-15.41) | −5.00 (−6.38 to −3.88) | 6549.5 | <.001 | <.001 | Yes |
| GPT 5.2 (Baseline) | OSS-20b (FRES Prompt) | 4.38 (3.97-4.80) | 7.66 (5.22-15.41) | −5.18 (−6.56 to −4.08) | 5048.0 | <.001 | <.001 | Yes |
| GPT 5.2 (Semantic) | Gemini 3 (Baseline) | 5.60 (4.95-6.39) | 13.25 (12.16-14.65) | −7.73 (−7.91 to −7.56) | 0.0 | <.001 | <.001 | Yes |
| OSS-20b (Semantic) | OSS-20b (FRES Prompt) | 0.91 (0.74-2.86) | 7.66 (5.22-15.41) | −7.89 (−9.02 to −7.04) | 785.0 | <.001 | <.001 | Yes |
| GPT 5.2 (Textstat) | Gemini 3 (Baseline) | 5.36 (4.72-5.98) | 13.25 (12.16-14.65) | −8.03 (−8.23 to −7.83) | 1221.0 | <.001 | <.001 | Yes |
| GPT 5.2 (Semantic) | Gemini 3 (Semantic) | 5.60 (4.95-6.39) | 14.08 (12.80-15.68) | −8.50 (−8.70 to −8.30) | 0.0 | <.001 | <.001 | Yes |
| GPT 5.2 (FRES Prompt) | Gemini 3 (Baseline) | 4.58 (4.22-5.06) | 13.25 (12.16-14.65) | −8.71 (−8.91 to −8.52) | 0.0 | <.001 | <.001 | Yes |
| GPT 5.2 (Semantic) | Gemini 3 (Textstat) | 5.60 (4.95-6.39) | 14.40 (13.16-15.53) | −8.79 (−8.98 to −8.61) | 0.0 | <.001 | <.001 | Yes |
| GPT 5.2 (Textstat) | Gemini 3 (Semantic) | 5.36 (4.72-5.98) | 14.08 (12.80-15.68) | −8.84 (−9.07 to −8.61) | 1196.0 | <.001 | <.001 | Yes |
| GPT 5.2 (Baseline) | Gemini 3 (Baseline) | 4.38 (3.97-4.80) | 13.25 (12.16-14.65) | −8.95 (−9.14 to −8.76) | 598.0 | <.001 | <.001 | Yes |
| GPT 5.2 (Textstat) | Gemini 3 (Textstat) | 5.36 (4.72-5.98) | 14.40 (13.16-15.53) | −9.09 (−9.31 to −8.87) | 1123.0 | <.001 | <.001 | Yes |
| GPT 5.2 (FRES Prompt) | Gemini 3 (Semantic) | 4.58 (4.22-5.06) | 14.08 (12.80-15.68) | −9.49 (−9.71 to −9.28) | 0.0 | <.001 | <.001 | Yes |
| GPT 5.2 (Baseline) | Gemini 3 (Semantic) | 4.38 (3.97-4.80) | 14.08 (12.80-15.68) | −9.70 (−9.93 to −9.48) | 403.0 | <.001 | <.001 | Yes |
| GPT 5.2 (FRES Prompt) | Gemini 3 (Textstat) | 4.58 (4.22-5.06) | 14.40 (13.16-15.53) | −9.74 (−9.95 to −9.55) | 0.0 | <.001 | <.001 | Yes |
| GPT 5.2 (Baseline) | Gemini 3 (Textstat) | 4.38 (3.97-4.80) | 14.40 (13.16-15.53) | −9.99 (−10.20 to −9.79) | 488.0 | <.001 | <.001 | Yes |
| Gemini 3 (Semantic) | Gemini 3 (FRES Prompt) | 14.08 (12.80-15.68) | 25.95 (20.51-32.82) | −12.16 (−13.45 to −11.07) | 653.5 | <.001 | <.001 | Yes |
| Gemini 3 (Baseline) | Gemini 3 (FRES Prompt) | 13.25 (12.16-14.65) | 25.95 (20.51-32.82) | −13.21 (−14.40 to −12.18) | 76.0 | <.001 | <.001 | Yes |
| GPT 5.2 (Semantic) | Gemini 3 (FRES Prompt) | 5.60 (4.95-6.39) | 25.95 (20.51-32.82) | −20.98 (−22.20 to −19.94) | 0.0 | <.001 | <.001 | Yes |
| GPT 5.2 (Textstat) | Gemini 3 (FRES Prompt) | 5.36 (4.72-5.98) | 25.95 (20.51-32.82) | −21.36 (−22.54 to −20.30) | 517.0 | <.001 | <.001 | Yes |
| GPT 5.2 (FRES Prompt) | Gemini 3 (FRES Prompt) | 4.58 (4.22-5.06) | 25.95 (20.51-32.82) | −22.25 (−23.44 to −21.18) | 0.0 | <.001 | <.001 | Yes |
| GPT 5.2 (Baseline) | Gemini 3 (FRES Prompt) | 4.38 (3.97-4.80) | 25.95 (20.51-32.82) | −22.46 (−23.66 to −21.37) | 399.0 | <.001 | <.001 | Yes |

Latency is the end-to-end response time in seconds per item (n=400 paired observations per comparison). Paired differences are Hodges-Lehmann estimates with exact distribution-free 95% CIs. Pairwise comparisons used 2-sided Wilcoxon signed-rank tests with the Pratt method for zero differences, with P values adjusted across all 120 comparisons using the Holm procedure. P values are reported to 2 decimal places, except when P<.01 or when rounding would alter the interpretation of statistical significance. Values below .001 are reported as P<.001, and values above .99 as P>.99. Statistical significance was defined as Holm-adjusted P<.05. Praw indicates the unadjusted P value; PHolm, the Holm-adjusted P value; and Holm sig., statistical significance after Holm correction. Rows are ordered by descending median difference. Abbreviations: CI, confidence interval; FRES, Flesch Reading Ease Score; IQR, interquartile range.

**Table S4. Pairwise comparisons of estimated cost with Holm adjustment.**

| **Condition A** | **Condition B** | **Median A, US $ (IQR)** | **Median B, US $ (IQR)** | **Median difference, US $ (95% CI)** | **Wilcoxon W** | ***P*raw** | ***P*Holm** | **Holm sig.** |
| --- | --- | --- | --- | --- | --- | --- | --- | --- |
| Gemini 3 (FRES Prompt) | OSS-20b (Semantic) | 0.031 (0.026-0.037) | 0.00008 (0.00007-0.00009) | 0.032 (0.031-0.032) | 0.0 | <.001 | <.001 | Yes |
| Gemini 3 (FRES Prompt) | OSS-20b (Textstat) | 0.031 (0.026-0.037) | 0.00007 (0.00006-0.00008) | 0.032 (0.031-0.032) | 0.0 | <.001 | <.001 | Yes |
| Gemini 3 (FRES Prompt) | OSS-20b (Baseline) | 0.031 (0.026-0.037) | 0.00007 (0.00006-0.00008) | 0.032 (0.031-0.032) | 0.0 | <.001 | <.001 | Yes |
| Gemini 3 (FRES Prompt) | OSS-20b (FRES Prompt) | 0.031 (0.026-0.037) | 0.00025 (0.00017-0.00036) | 0.031 (0.031-0.032) | 0.0 | <.001 | <.001 | Yes |
| Gemini 3 (FRES Prompt) | Claude 4.5 (Baseline) | 0.031 (0.026-0.037) | 0.0077 (0.0073-0.0083) | 0.024 (0.023-0.025) | 0.0 | <.001 | <.001 | Yes |
| Gemini 3 (FRES Prompt) | Claude 4.5 (Textstat) | 0.031 (0.026-0.037) | 0.0079 (0.0075-0.0085) | 0.024 (0.023-0.024) | 0.0 | <.001 | <.001 | Yes |
| Gemini 3 (FRES Prompt) | Claude 4.5 (FRES Prompt) | 0.031 (0.026-0.037) | 0.010 (0.0095-0.011) | 0.022 (0.021-0.022) | 0.0 | <.001 | <.001 | Yes |
| Gemini 3 (FRES Prompt) | Claude 4.5 (Semantic) | 0.031 (0.026-0.037) | 0.010 (0.0096-0.011) | 0.021 (0.021-0.022) | 0.0 | <.001 | <.001 | Yes |
| Gemini 3 (Semantic) | OSS-20b (Semantic) | 0.020 (0.019-0.022) | 0.00008 (0.00007-0.00009) | 0.020 (0.020-0.021) | 0.0 | <.001 | <.001 | Yes |
| Gemini 3 (Semantic) | OSS-20b (Textstat) | 0.020 (0.019-0.022) | 0.00007 (0.00006-0.00008) | 0.020 (0.020-0.021) | 0.0 | <.001 | <.001 | Yes |
| Gemini 3 (Semantic) | OSS-20b (Baseline) | 0.020 (0.019-0.022) | 0.00007 (0.00006-0.00008) | 0.020 (0.020-0.021) | 0.0 | <.001 | <.001 | Yes |
| Gemini 3 (Semantic) | OSS-20b (FRES Prompt) | 0.020 (0.019-0.022) | 0.00025 (0.00017-0.00036) | 0.020 (0.020-0.020) | 0.0 | <.001 | <.001 | Yes |
| Gemini 3 (Textstat) | OSS-20b (Semantic) | 0.019 (0.018-0.021) | 0.00008 (0.00007-0.00009) | 0.020 (0.019-0.020) | 0.0 | <.001 | <.001 | Yes |
| Gemini 3 (Textstat) | OSS-20b (Textstat) | 0.019 (0.018-0.021) | 0.00007 (0.00006-0.00008) | 0.020 (0.019-0.020) | 0.0 | <.001 | <.001 | Yes |
| Gemini 3 (Textstat) | OSS-20b (Baseline) | 0.019 (0.018-0.021) | 0.00007 (0.00006-0.00008) | 0.020 (0.019-0.020) | 0.0 | <.001 | <.001 | Yes |
| Gemini 3 (Textstat) | OSS-20b (FRES Prompt) | 0.019 (0.018-0.021) | 0.00025 (0.00017-0.00036) | 0.019 (0.019-0.020) | 0.0 | <.001 | <.001 | Yes |
| Gemini 3 (Baseline) | OSS-20b (Semantic) | 0.018 (0.017-0.021) | 0.00008 (0.00007-0.00009) | 0.018 (0.018-0.019) | 0.0 | <.001 | <.001 | Yes |
| Gemini 3 (Baseline) | OSS-20b (Textstat) | 0.018 (0.017-0.021) | 0.00007 (0.00006-0.00008) | 0.018 (0.018-0.019) | 0.0 | <.001 | <.001 | Yes |
| Gemini 3 (Baseline) | OSS-20b (Baseline) | 0.018 (0.017-0.021) | 0.00007 (0.00006-0.00008) | 0.018 (0.018-0.019) | 0.0 | <.001 | <.001 | Yes |
| Gemini 3 (Baseline) | OSS-20b (FRES Prompt) | 0.018 (0.017-0.021) | 0.00025 (0.00017-0.00036) | 0.018 (0.018-0.018) | 0.0 | <.001 | <.001 | Yes |
| Gemini 3 (Semantic) | Claude 4.5 (Baseline) | 0.020 (0.019-0.022) | 0.0077 (0.0073-0.0083) | 0.013 (0.012-0.013) | 0.0 | <.001 | <.001 | Yes |
| Gemini 3 (Semantic) | Claude 4.5 (Textstat) | 0.020 (0.019-0.022) | 0.0079 (0.0075-0.0085) | 0.012 (0.012-0.013) | 0.0 | <.001 | <.001 | Yes |
| Gemini 3 (Textstat) | Claude 4.5 (Baseline) | 0.019 (0.018-0.021) | 0.0077 (0.0073-0.0083) | 0.012 (0.012-0.012) | 0.0 | <.001 | <.001 | Yes |
| Gemini 3 (FRES Prompt) | Gemini 3 (Textstat) | 0.031 (0.026-0.037) | 0.019 (0.018-0.021) | 0.012 (0.011-0.012) | 227.0 | <.001 | <.001 | Yes |
| Gemini 3 (Textstat) | Claude 4.5 (Textstat) | 0.019 (0.018-0.021) | 0.0079 (0.0075-0.0085) | 0.012 (0.011-0.012) | 0.0 | <.001 | <.001 | Yes |
| Gemini 3 (Baseline) | Claude 4.5 (Baseline) | 0.018 (0.017-0.021) | 0.0077 (0.0073-0.0083) | 0.011 (0.010-0.011) | 0.0 | <.001 | <.001 | Yes |
| Gemini 3 (Baseline) | Claude 4.5 (Textstat) | 0.018 (0.017-0.021) | 0.0079 (0.0075-0.0085) | 0.011 (0.010-0.011) | 0.0 | <.001 | <.001 | Yes |
| Gemini 3 (Semantic) | Claude 4.5 (FRES Prompt) | 0.020 (0.019-0.022) | 0.010 (0.0095-0.011) | 0.010 (0.010-0.011) | 0.0 | <.001 | <.001 | Yes |
| Claude 4.5 (Semantic) | OSS-20b (Semantic) | 0.010 (0.0096-0.011) | 0.00008 (0.00007-0.00009) | 0.010 (0.010-0.010) | 0.0 | <.001 | <.001 | Yes |
| Claude 4.5 (Semantic) | OSS-20b (Textstat) | 0.010 (0.0096-0.011) | 0.00007 (0.00006-0.00008) | 0.010 (0.010-0.010) | 0.0 | <.001 | <.001 | Yes |
| Gemini 3 (Semantic) | Claude 4.5 (Semantic) | 0.020 (0.019-0.022) | 0.010 (0.0096-0.011) | 0.010 (0.0099-0.010) | 0.0 | <.001 | <.001 | Yes |
| Claude 4.5 (Semantic) | OSS-20b (Baseline) | 0.010 (0.0096-0.011) | 0.00007 (0.00006-0.00008) | 0.010 (0.010-0.010) | 0.0 | <.001 | <.001 | Yes |
| Claude 4.5 (FRES Prompt) | OSS-20b (Semantic) | 0.010 (0.0095-0.011) | 0.00008 (0.00007-0.00009) | 0.0099 (0.0099-0.010) | 0.0 | <.001 | <.001 | Yes |
| Claude 4.5 (FRES Prompt) | OSS-20b (Textstat) | 0.010 (0.0095-0.011) | 0.00007 (0.00006-0.00008) | 0.0099 (0.0099-0.010) | 0.0 | <.001 | <.001 | Yes |
| Claude 4.5 (Semantic) | OSS-20b (FRES Prompt) | 0.010 (0.0096-0.011) | 0.00025 (0.00017-0.00036) | 0.0099 (0.0098-0.010) | 0.0 | <.001 | <.001 | Yes |
| Claude 4.5 (FRES Prompt) | OSS-20b (Baseline) | 0.010 (0.0095-0.011) | 0.00007 (0.00006-0.00008) | 0.0099 (0.0098-0.0100) | 0.0 | <.001 | <.001 | Yes |
| Claude 4.5 (FRES Prompt) | OSS-20b (FRES Prompt) | 0.010 (0.0095-0.011) | 0.00025 (0.00017-0.00036) | 0.0097 (0.0096-0.0098) | 0.0 | <.001 | <.001 | Yes |
| Gemini 3 (Textstat) | Claude 4.5 (FRES Prompt) | 0.019 (0.018-0.021) | 0.010 (0.0095-0.011) | 0.0096 (0.0093-0.0098) | 0.0 | <.001 | <.001 | Yes |
| Gemini 3 (Textstat) | Claude 4.5 (Semantic) | 0.019 (0.018-0.021) | 0.010 (0.0096-0.011) | 0.0094 (0.0091-0.0096) | 0.0 | <.001 | <.001 | Yes |
| Gemini 3 (Baseline) | Claude 4.5 (FRES Prompt) | 0.018 (0.017-0.021) | 0.010 (0.0095-0.011) | 0.0085 (0.0082-0.0087) | 0.0 | <.001 | <.001 | Yes |
| Gemini 3 (Baseline) | Claude 4.5 (Semantic) | 0.018 (0.017-0.021) | 0.010 (0.0096-0.011) | 0.0083 (0.0080-0.0085) | 0.0 | <.001 | <.001 | Yes |
| Claude 4.5 (Textstat) | OSS-20b (Semantic) | 0.0079 (0.0075-0.0085) | 0.00008 (0.00007-0.00009) | 0.0079 (0.0078-0.0080) | 0.0 | <.001 | <.001 | Yes |
| Claude 4.5 (Textstat) | OSS-20b (Textstat) | 0.0079 (0.0075-0.0085) | 0.00007 (0.00006-0.00008) | 0.0079 (0.0078-0.0080) | 0.0 | <.001 | <.001 | Yes |
| Claude 4.5 (Textstat) | OSS-20b (Baseline) | 0.0079 (0.0075-0.0085) | 0.00007 (0.00006-0.00008) | 0.0078 (0.0078-0.0079) | 0.0 | <.001 | <.001 | Yes |
| Claude 4.5 (Baseline) | OSS-20b (Semantic) | 0.0077 (0.0073-0.0083) | 0.00008 (0.00007-0.00009) | 0.0077 (0.0076-0.0078) | 0.0 | <.001 | <.001 | Yes |
| Claude 4.5 (Baseline) | OSS-20b (Textstat) | 0.0077 (0.0073-0.0083) | 0.00007 (0.00006-0.00008) | 0.0077 (0.0076-0.0078) | 0.0 | <.001 | <.001 | Yes |
| Claude 4.5 (Baseline) | OSS-20b (Baseline) | 0.0077 (0.0073-0.0083) | 0.00007 (0.00006-0.00008) | 0.0077 (0.0076-0.0077) | 0.0 | <.001 | <.001 | Yes |
| Claude 4.5 (Textstat) | OSS-20b (FRES Prompt) | 0.0079 (0.0075-0.0085) | 0.00025 (0.00017-0.00036) | 0.0076 (0.0076-0.0077) | 0.0 | <.001 | <.001 | Yes |
| Claude 4.5 (Baseline) | OSS-20b (FRES Prompt) | 0.0077 (0.0073-0.0083) | 0.00025 (0.00017-0.00036) | 0.0075 (0.0074-0.0075) | 0.0 | <.001 | <.001 | Yes |
| GPT 5.2 (FRES Prompt) | OSS-20b (Semantic) | 0.0032 (0.0031-0.0035) | 0.00008 (0.00007-0.00009) | 0.0032 (0.0032-0.0032) | 0.0 | <.001 | <.001 | Yes |
| GPT 5.2 (FRES Prompt) | OSS-20b (Textstat) | 0.0032 (0.0031-0.0035) | 0.00007 (0.00006-0.00008) | 0.0032 (0.0031-0.0032) | 0.0 | <.001 | <.001 | Yes |
| GPT 5.2 (FRES Prompt) | OSS-20b (Baseline) | 0.0032 (0.0031-0.0035) | 0.00007 (0.00006-0.00008) | 0.0032 (0.0031-0.0032) | 0.0 | <.001 | <.001 | Yes |
| GPT 5.2 (FRES Prompt) | OSS-20b (FRES Prompt) | 0.0032 (0.0031-0.0035) | 0.00025 (0.00017-0.00036) | 0.0030 (0.0029-0.0030) | 0.0 | <.001 | <.001 | Yes |
| GPT 5.2 (Semantic) | OSS-20b (Semantic) | 0.0030 (0.0028-0.0032) | 0.00008 (0.00007-0.00009) | 0.0030 (0.0029-0.0030) | 0.0 | <.001 | <.001 | Yes |
| GPT 5.2 (Semantic) | OSS-20b (Textstat) | 0.0030 (0.0028-0.0032) | 0.00007 (0.00006-0.00008) | 0.0030 (0.0029-0.0030) | 0.0 | <.001 | <.001 | Yes |
| GPT 5.2 (Semantic) | OSS-20b (Baseline) | 0.0030 (0.0028-0.0032) | 0.00007 (0.00006-0.00008) | 0.0029 (0.0029-0.0030) | 0.0 | <.001 | <.001 | Yes |
| GPT 5.2 (Baseline) | OSS-20b (Semantic) | 0.0029 (0.0028-0.0031) | 0.00008 (0.00007-0.00009) | 0.0029 (0.0028-0.0029) | 0.0 | <.001 | <.001 | Yes |
| GPT 5.2 (Baseline) | OSS-20b (Textstat) | 0.0029 (0.0028-0.0031) | 0.00007 (0.00006-0.00008) | 0.0029 (0.0028-0.0029) | 0.0 | <.001 | <.001 | Yes |
| GPT 5.2 (Baseline) | OSS-20b (Baseline) | 0.0029 (0.0028-0.0031) | 0.00007 (0.00006-0.00008) | 0.0028 (0.0028-0.0029) | 0.0 | <.001 | <.001 | Yes |
| GPT 5.2 (Textstat) | OSS-20b (Semantic) | 0.0028 (0.0027-0.0030) | 0.00008 (0.00007-0.00009) | 0.0028 (0.0027-0.0028) | 0.0 | <.001 | <.001 | Yes |
| GPT 5.2 (Textstat) | OSS-20b (Textstat) | 0.0028 (0.0027-0.0030) | 0.00007 (0.00006-0.00008) | 0.0028 (0.0027-0.0028) | 0.0 | <.001 | <.001 | Yes |
| GPT 5.2 (Textstat) | OSS-20b (Baseline) | 0.0028 (0.0027-0.0030) | 0.00007 (0.00006-0.00008) | 0.0027 (0.0027-0.0028) | 0.0 | <.001 | <.001 | Yes |
| GPT 5.2 (Semantic) | OSS-20b (FRES Prompt) | 0.0030 (0.0028-0.0032) | 0.00025 (0.00017-0.00036) | 0.0027 (0.0027-0.0028) | 1.0 | <.001 | <.001 | Yes |
| GPT 5.2 (Baseline) | OSS-20b (FRES Prompt) | 0.0029 (0.0028-0.0031) | 0.00025 (0.00017-0.00036) | 0.0027 (0.0026-0.0027) | 5.0 | <.001 | <.001 | Yes |
| GPT 5.2 (Textstat) | OSS-20b (FRES Prompt) | 0.0028 (0.0027-0.0030) | 0.00025 (0.00017-0.00036) | 0.0026 (0.0025-0.0026) | 15.0 | <.001 | <.001 | Yes |
| Claude 4.5 (Semantic) | Claude 4.5 (Textstat) | 0.010 (0.0096-0.011) | 0.0079 (0.0075-0.0085) | 0.0023 (0.0022-0.0023) | 0.0 | <.001 | <.001 | Yes |
| Claude 4.5 (FRES Prompt) | Claude 4.5 (Textstat) | 0.010 (0.0095-0.011) | 0.0079 (0.0075-0.0085) | 0.0020 (0.0020-0.0021) | 0.0 | <.001 | <.001 | Yes |
| Gemini 3 (Semantic) | Gemini 3 (Textstat) | 0.020 (0.019-0.022) | 0.019 (0.018-0.021) | 0.00069 (0.00039-0.00099) | 29,744.0 | <.001 | <.001 | Yes |
| GPT 5.2 (FRES Prompt) | GPT 5.2 (Textstat) | 0.0032 (0.0031-0.0035) | 0.0028 (0.0027-0.0030) | 0.00042 (0.00040-0.00044) | 26.0 | <.001 | <.001 | Yes |
| Claude 4.5 (Semantic) | Claude 4.5 (FRES Prompt) | 0.010 (0.0096-0.011) | 0.010 (0.0095-0.011) | 0.00020 (0.00015-0.00026) | 24,426.0 | <.001 | <.001 | Yes |
| OSS-20b (FRES Prompt) | OSS-20b (Textstat) | 0.00025 (0.00017-0.00036) | 0.00007 (0.00006-0.00008) | 0.00019 (0.00017-0.00020) | 2222.0 | <.001 | <.001 | Yes |
| GPT 5.2 (Semantic) | GPT 5.2 (Textstat) | 0.0030 (0.0028-0.0032) | 0.0028 (0.0027-0.0030) | 0.00019 (0.00016-0.00021) | 6988.0 | <.001 | <.001 | Yes |
| GPT 5.2 (Baseline) | GPT 5.2 (Textstat) | 0.0029 (0.0028-0.0031) | 0.0028 (0.0027-0.0030) | 0.00010 (0.00008-0.00013) | 18,448.0 | <.001 | <.001 | Yes |
| OSS-20b (Semantic) | OSS-20b (Textstat) | 0.00008 (0.00007-0.00009) | 0.00007 (0.00006-0.00008) | 0.00001 (0.00001-0.00001) | 17,988.0 | <.001 | <.001 | Yes |
| OSS-20b (Baseline) | OSS-20b (Textstat) | 0.00007 (0.00006-0.00008) | 0.00007 (0.00006-0.00008) | 0.0000010 (−0.0000010 to 0.0000012) | 39,223.0 | .70 | .70 | No |
| OSS-20b (Baseline) | OSS-20b (Semantic) | 0.00007 (0.00006-0.00008) | 0.00008 (0.00007-0.00009) | −0.00001 (−0.00001 to −0.00001) | 28,518.0 | <.001 | <.001 | Yes |
| GPT 5.2 (Baseline) | GPT 5.2 (Semantic) | 0.0029 (0.0028-0.0031) | 0.0030 (0.0028-0.0032) | −0.00009 (−0.00010 to −0.00007) | 21,663.0 | <.001 | <.001 | Yes |
| OSS-20b (Baseline) | OSS-20b (FRES Prompt) | 0.00007 (0.00006-0.00008) | 0.00025 (0.00017-0.00036) | −0.00017 (−0.00019 to −0.00016) | 6723.0 | <.001 | <.001 | Yes |
| OSS-20b (Semantic) | OSS-20b (FRES Prompt) | 0.00008 (0.00007-0.00009) | 0.00025 (0.00017-0.00036) | −0.00018 (−0.00020 to −0.00017) | 28.0 | <.001 | <.001 | Yes |
| Claude 4.5 (Baseline) | Claude 4.5 (Textstat) | 0.0077 (0.0073-0.0083) | 0.0079 (0.0075-0.0085) | −0.00018 (−0.00022 to −0.00014) | 18,910.0 | <.001 | <.001 | Yes |
| GPT 5.2 (Semantic) | GPT 5.2 (FRES Prompt) | 0.0030 (0.0028-0.0032) | 0.0032 (0.0031-0.0035) | −0.00023 (−0.00025 to −0.00021) | 3076.0 | <.001 | <.001 | Yes |
| GPT 5.2 (Baseline) | GPT 5.2 (FRES Prompt) | 0.0029 (0.0028-0.0031) | 0.0032 (0.0031-0.0035) | −0.00031 (−0.00033 to −0.00029) | 1105.0 | <.001 | <.001 | Yes |
| Gemini 3 (Baseline) | Gemini 3 (Textstat) | 0.018 (0.017-0.021) | 0.019 (0.018-0.021) | −0.0012 (−0.0015 to −0.00099) | 18,374.0 | <.001 | <.001 | Yes |
| Gemini 3 (Baseline) | Gemini 3 (Semantic) | 0.018 (0.017-0.021) | 0.020 (0.019-0.022) | −0.0019 (−0.0022 to −0.0016) | 12,821.0 | <.001 | <.001 | Yes |
| Claude 4.5 (Baseline) | Claude 4.5 (FRES Prompt) | 0.0077 (0.0073-0.0083) | 0.010 (0.0095-0.011) | −0.0022 (−0.0023 to −0.0022) | 0.0 | <.001 | <.001 | Yes |
| Claude 4.5 (Baseline) | Claude 4.5 (Semantic) | 0.0077 (0.0073-0.0083) | 0.010 (0.0096-0.011) | −0.0024 (−0.0025 to −0.0024) | 0.0 | <.001 | <.001 | Yes |
| GPT 5.2 (FRES Prompt) | Claude 4.5 (Baseline) | 0.0032 (0.0031-0.0035) | 0.0077 (0.0073-0.0083) | −0.0045 (−0.0046 to −0.0045) | 0.0 | <.001 | <.001 | Yes |
| GPT 5.2 (FRES Prompt) | Claude 4.5 (Textstat) | 0.0032 (0.0031-0.0035) | 0.0079 (0.0075-0.0085) | −0.0047 (−0.0047 to −0.0046) | 0.0 | <.001 | <.001 | Yes |
| GPT 5.2 (Semantic) | Claude 4.5 (Baseline) | 0.0030 (0.0028-0.0032) | 0.0077 (0.0073-0.0083) | −0.0047 (−0.0048 to −0.0047) | 0.0 | <.001 | <.001 | Yes |
| GPT 5.2 (Baseline) | Claude 4.5 (Baseline) | 0.0029 (0.0028-0.0031) | 0.0077 (0.0073-0.0083) | −0.0048 (−0.0049 to −0.0048) | 0.0 | <.001 | <.001 | Yes |
| GPT 5.2 (Textstat) | Claude 4.5 (Baseline) | 0.0028 (0.0027-0.0030) | 0.0077 (0.0073-0.0083) | −0.0049 (−0.0050 to −0.0049) | 0.0 | <.001 | <.001 | Yes |
| GPT 5.2 (Semantic) | Claude 4.5 (Textstat) | 0.0030 (0.0028-0.0032) | 0.0079 (0.0075-0.0085) | −0.0049 (−0.0050 to −0.0049) | 0.0 | <.001 | <.001 | Yes |
| GPT 5.2 (Baseline) | Claude 4.5 (Textstat) | 0.0029 (0.0028-0.0031) | 0.0079 (0.0075-0.0085) | −0.0050 (−0.0051 to −0.0050) | 0.0 | <.001 | <.001 | Yes |
| GPT 5.2 (Textstat) | Claude 4.5 (Textstat) | 0.0028 (0.0027-0.0030) | 0.0079 (0.0075-0.0085) | −0.0051 (−0.0052 to −0.0051) | 0.0 | <.001 | <.001 | Yes |
| GPT 5.2 (FRES Prompt) | Claude 4.5 (FRES Prompt) | 0.0032 (0.0031-0.0035) | 0.010 (0.0095-0.011) | −0.0067 (−0.0068 to −0.0067) | 0.0 | <.001 | <.001 | Yes |
| GPT 5.2 (FRES Prompt) | Claude 4.5 (Semantic) | 0.0032 (0.0031-0.0035) | 0.010 (0.0096-0.011) | −0.0070 (−0.0070 to −0.0069) | 0.0 | <.001 | <.001 | Yes |
| GPT 5.2 (Semantic) | Claude 4.5 (FRES Prompt) | 0.0030 (0.0028-0.0032) | 0.010 (0.0095-0.011) | −0.0070 (−0.0070 to −0.0069) | 0.0 | <.001 | <.001 | Yes |
| GPT 5.2 (Baseline) | Claude 4.5 (FRES Prompt) | 0.0029 (0.0028-0.0031) | 0.010 (0.0095-0.011) | −0.0071 (−0.0071 to −0.0070) | 0.0 | <.001 | <.001 | Yes |
| GPT 5.2 (Textstat) | Claude 4.5 (FRES Prompt) | 0.0028 (0.0027-0.0030) | 0.010 (0.0095-0.011) | −0.0072 (−0.0072 to −0.0071) | 0.0 | <.001 | <.001 | Yes |
| GPT 5.2 (Semantic) | Claude 4.5 (Semantic) | 0.0030 (0.0028-0.0032) | 0.010 (0.0096-0.011) | −0.0072 (−0.0073 to −0.0071) | 0.0 | <.001 | <.001 | Yes |
| GPT 5.2 (Baseline) | Claude 4.5 (Semantic) | 0.0029 (0.0028-0.0031) | 0.010 (0.0096-0.011) | −0.0073 (−0.0074 to −0.0072) | 0.0 | <.001 | <.001 | Yes |
| GPT 5.2 (Textstat) | Claude 4.5 (Semantic) | 0.0028 (0.0027-0.0030) | 0.010 (0.0096-0.011) | −0.0074 (−0.0075 to −0.0073) | 0.0 | <.001 | <.001 | Yes |
| Gemini 3 (Semantic) | Gemini 3 (FRES Prompt) | 0.020 (0.019-0.022) | 0.031 (0.026-0.037) | −0.011 (−0.012 to −0.010) | 838.0 | <.001 | <.001 | Yes |
| Gemini 3 (Baseline) | Gemini 3 (FRES Prompt) | 0.018 (0.017-0.021) | 0.031 (0.026-0.037) | −0.013 (−0.014 to −0.012) | 0.0 | <.001 | <.001 | Yes |
| GPT 5.2 (FRES Prompt) | Gemini 3 (Baseline) | 0.0032 (0.0031-0.0035) | 0.018 (0.017-0.021) | −0.015 (−0.016 to −0.015) | 0.0 | <.001 | <.001 | Yes |
| GPT 5.2 (Semantic) | Gemini 3 (Baseline) | 0.0030 (0.0028-0.0032) | 0.018 (0.017-0.021) | −0.015 (−0.016 to −0.015) | 0.0 | <.001 | <.001 | Yes |
| GPT 5.2 (Baseline) | Gemini 3 (Baseline) | 0.0029 (0.0028-0.0031) | 0.018 (0.017-0.021) | −0.016 (−0.016 to −0.015) | 0.0 | <.001 | <.001 | Yes |
| GPT 5.2 (Textstat) | Gemini 3 (Baseline) | 0.0028 (0.0027-0.0030) | 0.018 (0.017-0.021) | −0.016 (−0.016 to −0.015) | 0.0 | <.001 | <.001 | Yes |
| GPT 5.2 (FRES Prompt) | Gemini 3 (Textstat) | 0.0032 (0.0031-0.0035) | 0.019 (0.018-0.021) | −0.016 (−0.017 to −0.016) | 0.0 | <.001 | <.001 | Yes |
| GPT 5.2 (Semantic) | Gemini 3 (Textstat) | 0.0030 (0.0028-0.0032) | 0.019 (0.018-0.021) | −0.017 (−0.017 to −0.016) | 0.0 | <.001 | <.001 | Yes |
| GPT 5.2 (Baseline) | Gemini 3 (Textstat) | 0.0029 (0.0028-0.0031) | 0.019 (0.018-0.021) | −0.017 (−0.017 to −0.016) | 0.0 | <.001 | <.001 | Yes |
| GPT 5.2 (Textstat) | Gemini 3 (Textstat) | 0.0028 (0.0027-0.0030) | 0.019 (0.018-0.021) | −0.017 (−0.017 to −0.016) | 0.0 | <.001 | <.001 | Yes |
| GPT 5.2 (FRES Prompt) | Gemini 3 (Semantic) | 0.0032 (0.0031-0.0035) | 0.020 (0.019-0.022) | −0.017 (−0.017 to −0.017) | 0.0 | <.001 | <.001 | Yes |
| GPT 5.2 (Semantic) | Gemini 3 (Semantic) | 0.0030 (0.0028-0.0032) | 0.020 (0.019-0.022) | −0.017 (−0.018 to −0.017) | 0.0 | <.001 | <.001 | Yes |
| GPT 5.2 (Baseline) | Gemini 3 (Semantic) | 0.0029 (0.0028-0.0031) | 0.020 (0.019-0.022) | −0.017 (−0.018 to −0.017) | 0.0 | <.001 | <.001 | Yes |
| GPT 5.2 (Textstat) | Gemini 3 (Semantic) | 0.0028 (0.0027-0.0030) | 0.020 (0.019-0.022) | −0.018 (−0.018 to −0.017) | 0.0 | <.001 | <.001 | Yes |
| GPT 5.2 (FRES Prompt) | Gemini 3 (FRES Prompt) | 0.0032 (0.0031-0.0035) | 0.031 (0.026-0.037) | −0.028 (−0.029 to −0.028) | 0.0 | <.001 | <.001 | Yes |
| GPT 5.2 (Semantic) | Gemini 3 (FRES Prompt) | 0.0030 (0.0028-0.0032) | 0.031 (0.026-0.037) | −0.029 (−0.029 to −0.028) | 0.0 | <.001 | <.001 | Yes |
| GPT 5.2 (Baseline) | Gemini 3 (FRES Prompt) | 0.0029 (0.0028-0.0031) | 0.031 (0.026-0.037) | −0.029 (−0.029 to −0.028) | 0.0 | <.001 | <.001 | Yes |
| GPT 5.2 (Textstat) | Gemini 3 (FRES Prompt) | 0.0028 (0.0027-0.0030) | 0.031 (0.026-0.037) | −0.029 (−0.030 to −0.028) | 0.0 | <.001 | <.001 | Yes |

Cost is the estimated application programming interface cost per response in US dollars (n=400 paired observations per comparison), computed by multiplying provider-reported per-million-token prices by the input and output token counts of each response; input and output are priced separately. Paired differences are Hodges-Lehmann estimates with exact distribution-free 95% CIs. Pairwise comparisons used 2-sided Wilcoxon signed-rank tests with the Pratt method for zero differences, with P values adjusted across all 120 comparisons using the Holm procedure. P values are reported to 2 decimal places, except when P<.01 or when rounding would alter the interpretation of statistical significance. Values below .001 are reported as P<.001, and values above .99 as P>.99. Statistical significance was defined as Holm-adjusted P<.05. Praw indicates the unadjusted P value; PHolm, the Holm-adjusted P value; and Holm sig., statistical significance after Holm correction. Rows are ordered by descending median difference. Abbreviations: CI, confidence interval; FRES, Flesch Reading Ease Score; IQR, interquartile range. Values for OSS-20b (Baseline) vs OSS-20b (Textstat) are shown with additional decimal places because the paired difference was below US $0.00001.
