# Supplementary material for: Prompting Strategies for Readability Alignment Between User-Generated Ophthalmology Questions and Large Language Model Answers: Comparative Evaluation Study
Source: JMIR Form Res. 2026 Nov 5;10:e97408. doi: 10.2196/97408 (PMC13739205; doi:10.2196/97408)
Supplement: Supplementary file 12 [file formative-v10-e97408-s013.docx]

**TRIPOD-LLM Reporting Checklist**

***Supplementary file for editors and peer reviewers only — not intended for publication***

**Prompting Strategies for Readability Alignment Between User-Generated Ophthalmology Questions and Large Language Model Answers: Comparative Evaluation Study**

Applicable study categories: evaluation of existing LLMs (E), health care setting (H), question answering (QA), and readability adaptation/simplification (SS).

This checklist documents reporting in the revised manuscript. Items limited to de novo model development, fine-tuning, EHR-based prediction, outcome forecasting, or model updating are marked not applicable. Locations are identified by manuscript section because pagination may change during production.

# Completed checklist

| **Item / section** | **TRIPOD-LLM recommendation** | **Applicability** | **Location in revised manuscript** | **How addressed** |
| --- | --- | --- | --- | --- |
| 1 — Title | Identify the study as developing, fine-tuning, and/or evaluating an LLM, specifying the task, target population, and outcome. | Applicable | Title; Abstract | The title identifies a comparative evaluation of prompting strategies for readability alignment between user-generated ophthalmology questions and LLM answers. |
| 2 — Abstract | See TRIPOD-LLM for abstracts. | Applicable | Abstract | The structured abstract reports the health communication context, objective, dataset, models, prompting conditions, outcomes, principal results, and conclusions. |
| 3a — Introduction: Background | Explain the health care context/use case and rationale for developing or evaluating the LLM, including existing approaches and models. | Applicable | Introduction, paragraphs 1-4 | The manuscript describes readability barriers in patient education, prior LLM simplification approaches, and the rationale for question-anchored readability adaptation. |
| 3b — Introduction: Background | Describe the target population and intended use of the LLM, including intended users and current practice. | Applicable | Introduction; Discussion—Comparison With Prior Work | The target population is represented by users asking ophthalmology questions online. The intended application is clinician-facing tools that generate readability-adapted responses for digital patient communication, with clinician review before delivery. |
| 4 — Introduction: Objectives | Specify the study objectives and whether the study concerns initial development, fine-tuning, validation, or evaluation. | Applicable | End of Introduction; Methods—Overview of Study Design | The study is described as a controlled comparative, formative predeployment evaluation of existing LLMs. No model development or fine-tuning was performed. |
| 5a — Methods: Data | Describe data sources separately for training, tuning, and evaluation datasets and the rationale for their use. | Applicable | Methods—Question Collection and Selection | Evaluation questions were collected from public ophthalmology-related YouTube comments. No training or tuning dataset was used because existing LLMs were evaluated without fine-tuning. |
| 5b — Methods: Data | Describe relevant data points and provide quantitative and qualitative information about their distribution. | Applicable | Methods—Question Collection and Selection; Table 1 | The manuscript reports the source pool, final sample, readability tiers, question-length strata, thematic domains, and question types. |
| 5c — Methods: Data | State the dates of the oldest and newest evaluation text and, for existing LLMs, report developer-provided training or knowledge cutoffs when available. | Partially reported | Methods—Question Collection and Selection; Methods—Large Language Models | The manuscript reports the comment-collection period (December 1-10, 2025), the inference period (December 23, 2025-January 15, 2026), and the developer-reported knowledge cutoffs of the evaluated models. The original posting dates of the oldest and newest comments are not reported. |
| 5d — Methods: Data | Describe data preprocessing and quality checking. | Applicable | Methods—Question Collection and Selection | Screening, exclusion criteria, English-language restriction, preservation of original wording, stratified sampling, and ophthalmologist classification are reported. |
| 5e — Methods: Data | Describe handling of missing and imbalanced data and reasons for omitting data. | Partially reported | Methods—Question Collection and Selection | Stratified random sampling with predefined quotas was used to ensure representation across readability and question-length strata. Missing-data handling and imputation are not explicitly reported. |
| 6a — Methods: Analytical methods | Report the LLM name, version, and last date of training or knowledge cutoff. | Applicable | Methods—Large Language Models | Model names, versions, inference period, providers, and knowledge cutoffs are reported. |
| 6b — Methods: Analytical methods | Report LLM architecture, training, fine-tuning procedures, alignment strategy, and alignment goals. | Not applicable to study development | Methods—Large Language Models | No LLM was developed or fine-tuned. The study used existing proprietary and open-source models; proprietary architecture and full training details were not publicly available. |
| 6c — Methods: Analytical methods | Report how text was generated, including prompt engineering, consistency procedures, and inference settings. | Applicable | Methods—Large Language Models; Methods—Readability Alignment Conditions; Multimedia Appendix 1 | Generation was isolated per question. APIs, inference dates, temperature, top-k, logging, and complete prompt templates are reported. |
| 6d — Methods: Analytical methods | Specify the initial and postprocessed LLM output. | Applicable | Multimedia Appendix 5 | Initial outputs from the FRES Prompt and Semantic conditions contained a model-generated readability metadata line followed by the substantive response. This metadata line was parsed separately and removed from the final user-facing response; readability analyses were performed on the substantive response only. |
| 6e — Methods: Analytical methods | Provide details and rationale for classification, probability determination, and thresholds, when applicable. | Applicable | Methods—Readability Assessment and Categorization | FRES thresholds for Basic, Intermediate, and Higher categories and their rationale are reported. No probability threshold was used. |
| 7a — Methods: LLM output | Include metrics that capture generative-output quality, such as consistency, relevance, accuracy, and error type. | Applicable | Methods---Readability Assessment and Categorization; Response Quality Evaluation; Computational Cost Analysis; Results | Readability alignment, factual accuracy, completeness, empathy, BERTScore, latency, token use, and estimated cost are reported. |
| 7b — Methods: LLM output | Explain the relevance of outcome metrics to downstream use and, where applicable, their relationship to human evaluation. | Applicable | Introduction; Methods—Human Validation; Discussion | Readability alignment is linked to audience-adapted communication, and LLM-as-a-judge outcomes were compared with ratings from two human evaluators. |
| 7c — Methods: LLM output | Clearly define outcomes, calculations, code/API, inference dates, and evaluation metrics. | Applicable | Methods—Readability Assessment and Categorization; Response Quality Evaluation; Statistical Analysis | Outcomes, calculations, inference dates, API providers, and evaluation metrics are reported. The analysis code is available from the corresponding author upon reasonable request. |
| 7d — Methods: LLM output | For subjective outcome assessment, report assessor qualifications, instructions, relevant characteristics, and interassessor agreement. | Applicable | Methods—Response Quality Evaluation; Human Validation; Results—Agreement Between LLM-as-a-Judge and Human Evaluation | A senior ophthalmologist and an ophthalmology resident used identical prespecified criteria. Agreement among evaluators is reported. |
| 7e — Methods: LLM output | Specify how performance was compared with other LLMs, humans, benchmarks, or standards. | Applicable | Methods—Overview of Study Design; Human Validation; Statistical Analysis | Four LLMs and four prompting conditions were compared. LLM judgments were validated against two human evaluators. |
| 8a — Methods: Annotation | If annotation was performed, describe labeling procedures and provide annotation guidance with examples. | Applicable | Methods—Question Collection and Selection; Ground Truth Generation; Response Quality Evaluation; Multimedia Appendices 5 and 6 | Question labeling and quality-rating criteria are explicitly defined, and examples are provided. |
| 8b — Methods: Annotation | Report the number of annotators, multiply annotated proportion, and interannotator agreement. | Applicable | Methods---Human Validation; Results---Agreement Between LLM-as-a-Judge and Human Evaluation | Two human evaluators independently rated the same stratified 10% sample: 40 questions and 640 question-response pairs. Agreement statistics are reported. |
| 8c — Methods: Annotation | Provide information on annotator background and experience or characteristics of models used for labeling. | Applicable | Methods—Ground Truth Generation; Human Validation | The human evaluators are identified as a senior ophthalmologist and an ophthalmology resident. Reference answers were supervised and approved by a board-certified ophthalmologist. |
| 9a — Methods: Prompting | If prompting was used, describe prompt design, curation, and selection. | Applicable | Methods—Readability Alignment Conditions | Prompt templates were defined a priori using established best practices and experience from a previous study, while maintaining consistent safety, length, and formatting instructions across conditions. |
| 9b — Methods: Prompting | Report what data were used to develop the prompts. | Applicable | Methods—Readability Alignment Conditions | The evaluation questions and responses were not used to optimize or revise the prompt templates. |
| 10 — Methods: Summarization | Describe preprocessing before summarization. | Not applicable | Not applicable | The study did not evaluate summarization. |
| 11 — Methods: Instruction tuning/alignment | If instruction tuning or alignment strategies were used, report the instructions, data, evaluation interface, and evaluator characteristics. | Not applicable to model tuning | Methods—Readability Alignment Conditions; Multimedia Appendix 1 | No parameter-level instruction tuning or model alignment was performed. Prompt-based readability control was evaluated, and full instructions are provided. |
| 12 — Methods: Compute | Report compute or proxies such as hardware, cost, inference time, or machine count. | Applicable | Methods—Computational Cost Analysis; Results—Computational Efficiency | Token use, latency, and estimated API cost are reported. Provider hardware was not controlled by the investigators. |
| 13 — Methods: Ethical approval | Name the ethics board and report consent or waiver. | Applicable | Methods—Ethical Considerations | The manuscript states that ethics committee approval was not required under Brazilian National Health Council Resolution No. 510/2016 because the study analyzed publicly available information without identifiable personal information. Informed consent or a formal consent waiver is not separately reported. |
| 14a — Open science | Give the source of funding and the role of funders. | Applicable | Funding | The study received no external funding. |
| 14b — Open science | Declare conflicts of interest and financial disclosures. | Applicable | Conflicts of Interest | None declared. |
| 14c — Open science | Indicate where the study protocol can be accessed or state that no protocol was prepared. | Applicable | Methods-Overview of Study Design | A study protocol was prepared but not publicly registered; the section relevant to this study is available from the corresponding author upon reasonable request. |
| 14d — Open science | Provide registration information or state that the study was not registered. | Applicable | Methods-Overview of Study Design | The study was not registered in a public study registry. |
| 14e — Open science | Provide details of study-data availability. | Applicable | Data Availability | Restrictions on redistributing original user-generated comments and access to derived data, model responses, ratings, and scripts are described. |
| 14f — Open science | Provide details of code availability. | Applicable | Data Availability | Analysis scripts are available from the corresponding author upon reasonable request. |
| 15 — Public involvement | Describe patient and public involvement or state that there was none. | Applicable | Not reported in the manuscript; stated in this supplementary checklist | Patients and members of the public were not involved in the design, conduct, interpretation, or reporting of the study. |
| 16a — Results: Participants/data | For patient/EHR data, describe data flow and numbers with and without outcomes or labels. | Not applicable; analogous data flow reported | Methods—Question Collection and Selection; Results—Question Characteristics | No patient or EHR records were used. The flow from the source pool of 3011 questions to the stratified sample of 400 is reported. |
| 16b — Results: Participants/data | For patient/EHR data, report characteristics by source or setting and development/evaluation split. | Not applicable; analogous details reported | Methods—Question Collection and Selection; Table 1 | No patient/EHR data were used. Source, dates, sample size, topic distribution, readability, length, and question type are reported. |
| 16c — Results: Participants/data | For LLM evaluations with clinical outcomes, compare important clinical-variable distributions between development and evaluation datasets. | Not applicable | Not applicable | The study did not predict clinical outcomes and had no development/evaluation patient cohorts. |
| 16d — Results: Participants/data | For patient/EHR data, report participants and outcome events in each analysis. | Not applicable; denominators reported | Methods and Results | There were no patient-level outcomes. Denominators for questions, generated responses, and human-validation ratings are reported. |
| 17 — Results: Performance | Report LLM performance according to prespecified metrics and/or human evaluation. | Applicable | Results; Tables 2 and 3Figures 1-6; Multimedia Appendices 2-11 | Prespecified readability, quality, agreement, efficiency, and human-validation outcomes are reported with uncertainty and multiplicity-adjusted comparisons. |
| 18 — Results: LLM updating | If applicable, report results from LLM updating and subsequent performance. | Not applicable | Not applicable | No model was updated or fine-tuned. |
| 19a — Discussion: Interpretation | Interpret the main results, including fairness in relation to objectives and previous studies. | Applicable | Discussion—Principal Results; Comparison With Prior Work | The manuscript interprets model- and strategy-specific performance. Demographic fairness could not be assessed because user demographic information was unavailable. |
| 19b — Discussion: Limitations | Discuss limitations and effects on bias, statistical uncertainty, and generalizability. | Applicable | Discussion—Strengths and Limitations | Limitations include indirect readability proxies, absence of comprehension outcomes, English-only data, topic distribution, single-specialty scope and online-question sampling. |
| 19c — Discussion: Usability | Describe challenges in using data for the task, including representation, missingness, harmonization, and bias. | Applicable | Discussion—Strengths and Limitations | The discussion addresses unedited real-world questions, English-language restriction, uneven topic representation, and limitations of formula-based readability. |
| 19d — Discussion: Usability | Define intended implementation, input, end user, autonomy, and human oversight. | Applicable | Discussion—Comparison With Prior Work | The approach is intended to support clinician-facing tools that generate readability-adapted responses for digital patient communication, with mandatory clinician review before delivery. |
| 19e — Discussion: Usability | If applicable, explain how poor-quality or unavailable input should be assessed and handled. | Applicable | Methods—Question Collection and Selection; Discussion—Strengths and Limitations | Ambiguous, incoherent, nonmedical, or otherwise unsuitable questions were excluded. Such inputs should be flagged for clinician review in future implementations. |
| 19f — Discussion: Usability | If applicable, specify user interaction and required expertise. | Applicable | Discussion—Comparison With Prior Work | The intended user is a clinician. The system is a communication-assistance tool rather than an autonomous medical adviser, and professional review is required. |
| 19g — Discussion: Future research | Discuss future research, applicability, and generalizability. | Applicable | Discussion—Strengths and Limitations; Conclusions | Future work is proposed to assess comprehension, recall, actionability, decision outcomes, other languages, broader clinical domains, and prospective implementation. |
